# Harnessing Random Polymers as Chemoselective Catalysts With Structural Adaptability: Epoxidation of Olefinic Quaternary Ammonium Salts via Charge Recognition

**DOI:** 10.1002/chem.71082

**Published:** 2026-05-01

**Authors:** Yusei Fujii, Kiyosei Takasu, Yusuke Kuroda

**Affiliations:** ^1^ Graduate School of Pharmaceutical Sciences Kyoto University Kyoto Japan; ^2^ The HAKUBI Center for Advanced Research Kyoto University Kyoto Japan

**Keywords:** chemoselectivity, copolymerization, epoxidation, hydrogen bonding, polymer catalyst

## Abstract

The design of artificial chemoselective catalysts that function effectively across structurally diverse substrates remains a long‐standing challenge. In this study, we demonstrate that a random heteropolymer, synthesized via radical polymerization, can function as a versatile catalyst for the chemoselective epoxidation of structurally diverse olefinic quaternary ammonium salts. This three‐monomer‐based polymer catalyst exhibits exceptional selectivity and remarkable structural adaptability that cannot be achieved by conventional small‐molecule catalysts. Beyond reusability, our approach highlights the often‐overlooked advantages of polymer catalysis, paving the way for its application in a broad spectrum of chemical transformations.

## Graphical abstract

The development of chemoselective catalysts that operate across structurally diverse substrates remains a long‐standing challenge. Here, we demonstrate that a random heteropolymer bearing carboxylic acid and urea residues functions as a general epoxidation catalyst for olefinic quaternary ammonium salts. Its adaptive environment accommodates diverse substrates beyond conventional small‐molecule catalysts.

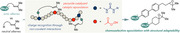

## 1 Introduction

**FIGURE 1:**
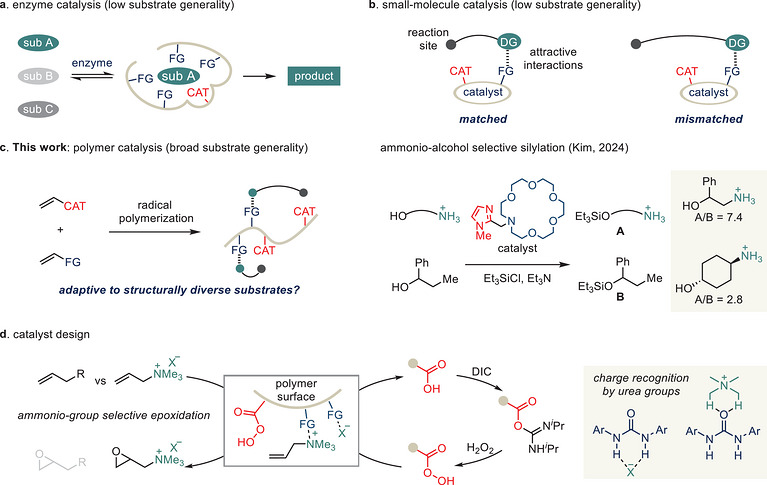
General‐oriented catalyst development for the chemoselective transformations with broad structural tolerance. (a) Conceptual depiction of enzymatic catalysis. (b) Conceptual depiction of small‐molecule catalysis. (c) Conceptual depiction of polymer catalysis (this work). (d) Design of the polymer catalyst for the selective epoxidation of olefinic quaternary ammonium salts.

Enzymes display an extraordinary capacity to convert one specific substrate with high chemoselectivity, even when a variety of potential reactants are present. This selectivity arises from the precise recognition of the 3D structure of the substrate by substrate‐recognition groups (FG) within the active site, which engage in noncovalent interactions to enable transformations that surpass the substrates’ inherent reactivity (Figure 1a) [1, 2]. In efforts to emulate the enzymatic function, artificial catalysts with minimal functional complexity have traditionally been designed by covalently linking the FG to a catalytic unit (CAT) [3, 4]. In such designs, attractive noncovalent interactions between the FG and a directing group (DG) embedded within the substrate serve to position the CAT in close proximity to the target reactive site, enabling the chemoselective transformation of DG‐containing substrates (Figure 1b, top). While these artificial catalysts often exhibited high selectivity for their model substrates, they are typically sensitive to variations in the spatial relationship between the DG and the reactive functionality. As a result, they often fail to maintain high selectivity when evaluated across a broader range of substrates [4, 5, 6, 7], as prominently evidenced by a recent work from Kim and coworkers on the selective silylation of alcohols bearing an ammonium group (Figure 1b, bottom) [5]. To avoid the necessity of designing bespoke catalysts for each substrate, the development of a universal catalyst that can chemoselectively transform a class of substrates while accommodating broad structural diversity would constitute a valuable advance in organic synthesis, where substrate generality remains a long‐standing objective [8, 9, 10, 11]. To overcome the fundamental trade‐off between selectivity and generality that constrains previous artificial catalysts, we sought to establish a fundamentally new design platform.

Compared to small‐molecule catalysts, the application of synthetic polymers as chemoselective catalysts remains relatively underexplored [12]. Early pioneering studies by Overberger [13, 14, 15, 16, 17, 18] and Kunitake [19, 20, 21, 22, 23, 24, 25, 26, 27, 28] independently demonstrated that imidazole–containing polymers can function as catalysts for the chemoselective solvolysis of *O*‐aryl esters. Around the same time, Grubbs and co‐workers observed molecular sieving behavior with a polystyrene‐bound rhodium complex in a competitive alkene reduction [29, 30, 31]. More recently, molecular imprinting has enabled substrate‐specific catalysis through the generation of complementary binding cavities [32, 33, 34, 35, 36, 37]. Despite these advances, the unique potential of polymer‐based catalysts has largely remained untapped, particularly beyond their well‐recognized advantages in recyclability and reusability [38, 39, 40, 41]. We hypothesized that a random heteropolymer comprising monomers bearing the CAT and the FG functionalities could serve as a versatile platform for chemoselective catalysts targeting ionic substrates. More specifically, owing to the statistical distribution of these moieties throughout the polymer surface [42], we envisioned that the resulting polymer could accommodate diverse substrate geometries (Figure 1c). Herein, we introduce a new strategy for chemoselective catalysis that is broadly applicable to diverse chemical transformations. In this context, we demonstrate the successful implementation of this concept through the chemoselective epoxidation of ionic alkene substrates.

The epoxidation of olefinic quaternary ammonium salts was selected as a testing ground for our hypothesis, given their synthetic accessibility, derivatization potential [43], and relevance to bioactive molecules such as phospholipids (Figure 1d) [44]. The success of our envisioned transformation hinged on identifying an optimal set of monomers that, upon polymerization, would work in synergy [45, 46, 47], to enable both selective recognition of the quaternary ammonium moiety and efficient epoxidation of the olefinic substrate. Moreover, effective catalytic turnover required reversible substrate binding to avoid product inhibition. Toward this end, we designed a random heteropolymer guided by two key criteria. First, we selected a carboxylic acid‐containing monomer to serve as the catalytic unit (CAT) for epoxidation [48, 49]. Inspired by Miller's precedent in aspartyl peptide‐catalyzed epoxidation [50, 51, 52, 53, 54, 55], we reasoned that activation of hydrogen peroxide by a pendant carboxylic acid in the presence of a carbodiimide would generate a percarboxylic acid residue that is sufficiently electrophilic to engage in the olefin epoxidation. Second, for functional group recognition, we incorporated a urea‐bearing monomer, hypothesizing that the carbonyl oxygen of the urea could engage in weak C–H···O hydrogen‐bonding or electrostatic interactions with the relatively acidic α‐protons adjacent to the quaternary ammonium center [56, 57, 58]. In the field of polymer chemistry, urea‐based architectures have been widely utilized as effective platforms for molecular recognition [59, 60]. Additionally, we anticipated that the urea moiety could serve as an anion receptor, forming complementary hydrogen‐bonding interactions with the counteranion of the quaternary ammonium salt [61, 62], thereby reinforcing substrate recognition. With these design elements in mind, we sought to identify optimal monomer structures and compositional ratios that would serve as a foundation for developing catalysts with structural adaptability.

**FIGURE 2:**
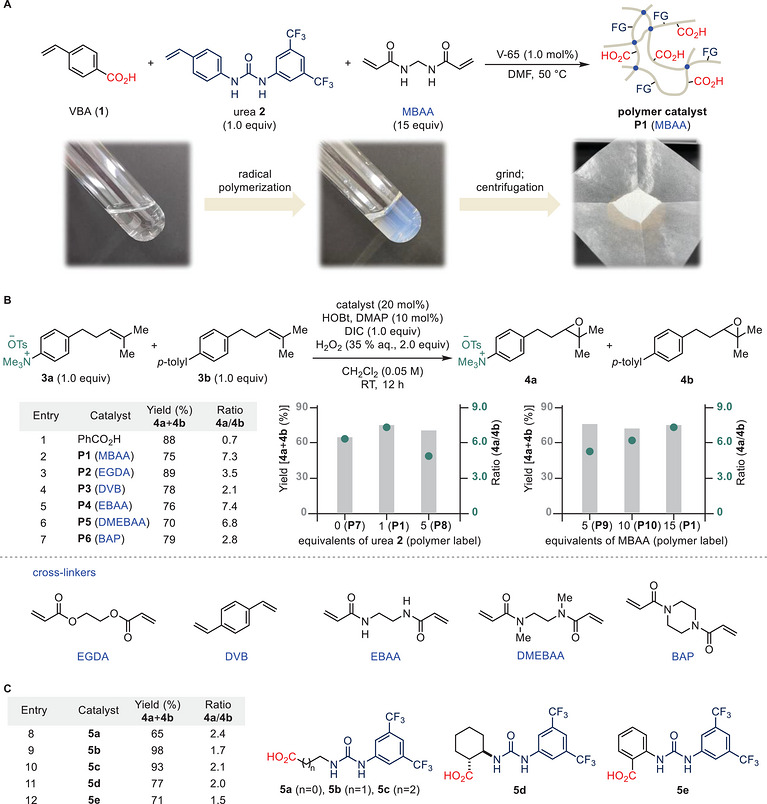
Development of a polymer catalyst for the chemoselective epoxidation of olefinic quaternary ammonium salts. (a) Preparation of polymer catalyst P1. (b) Evaluation of polymer catalysts. Gray bars indicate the combined yield of 4a and 4b, while green dots represent the 4a/4b ratio. (c) Comparison to small‐molecule catalysts.

Our investigation commenced with the preparation of statistical heteropolymers via free‐radical polymerization of 4‐vinylbenzoic acid (VBA, **1**), 1,3‐diarylurea monomer **2,** and a cross‐linking monomer (Figure 2a). As a representative procedure, a solution of **1**, **2,** and *N,N'*‐methylenebisacrylamide (MBAA) in *N*,*N*‐dimethylformamide (DMF) was heated at 50 °C in the presence of 2,2'‐azobis(2,4‐dimethylvaleronitrile) (V‐65) (1.0 mol% relative to total polymerizable units) as a radical initiator. Following the polymerization, the resulting solid material was ground into a powder, and unreacted monomers were removed by centrifugal filtration with MeOH, providing polymer catalyst **P1** as an insoluble white powder. The content of carboxy moiety (CO_2_H) on the polymer surface was determined by an acid‐base back titration [63]. Following the same procedure, a small library of polymer catalysts with various compositions of monomers was prepared (Figure S1).

We next evaluated the performance of polymer catalysts in the competitive epoxidation between olefinic trimethylammonium **3a** possessing a tosylate counterion and its neutral surrogate **3b** (Figure 2b). Using an adaptation of the reported protocol by Miller and coworkers [50, 51, 52, 53, 54, 55], a 1:1 mixture of **3a** and **3b** in CH_2_Cl_2_ was treated with *N,N'*‐diisopropylcarbodiimide (DIC) (1.0 equiv) and aqueous solution of H_2_O_2_ (2.0 equiv) in the presence of carboxylic acid catalyst (20 mol%), 1‐hydroxybenzotriazole (HOBt) (10 mol%), and 4‐dimethylaminopyridine (DMAP) (10 mol%) at room temperature. An initial evaluation of the intrinsic reactivities of **3a** and **3b** toward epoxidation using benzoic acid as a catalyst revealed inferior reactivity of the ionic alkene **3a** (**4a**/**4b** = 0.7), likely due to the electron‐withdrawing nature of the trimethylammonio group (entry 1) [64]. We were pleased to observe that polymer catalyst **P1**, synthesized with *N,N'*‐methylenebisacrylamide (MBAA) as a cross‐linking monomer, overrode the intrinsic reactivity of alkenes, providing **4a**/**4b** = 7.3 selectivity, favoring the ionic alkene **3a** (entry 2). It is worth noting that polymer catalyst **P2**, prepared with ethylene glycol diacrylate (EGDA) resulted in reduced selectivity (**4a**/**4b** = 3.5, entry 3), suggesting that the cross‐linking monomer had a substantial impact on the selectivity. Indeed, the use of divinylbenzene (DVB)‐based polymer catalyst **P3** further decreased the selectivity (**4a**/**4b** = 2.1, entry 4), hinting that amide groups on the polymer surface played a pivotal role in the recognition of quaternary ammonium substrates. Further investigation of the relationship between monomer structure and chemoselectivity revealed that polymer **P4**, derived from *N,N'*‐ethylenebisacrylamide (EBAA), maintained consistent selectivity (**4a**/**4b** = 7.4, entry 5). In contrast, its *N,N'*‐dimethylated analog **P5** exhibited reduced selectivity (**4a**/**4b** = 6.8, entry 6), which was further diminished with polymer **P6** cross‐linked with a more rigid 1,4‐bis(acryloyl)piperazine (BAP) (**4a**/**4b** = 2.8, entry 7). While catalysts **P1** and **P4** showed essentially identical catalytic performance in terms of both reactivity and selectivity, **P1** was selected as the representative catalyst for further studies owing to its substantially lower cost and greater practicality. With the optimal combination of monomers in hand, we next examined the impact of the monomer ratio on the catalytic performance. The contribution of the urea monomer **2** was validated by the reduced selectivity observed with catalyst **P7**, which lacks the urea functionality (**4a**/**4b** = 6.2, Figure 2b, left graph). However, increasing the equivalents of the urea monomer (5 equiv) beyond the standard composition (1 equiv) did not lead to further improvements in selectivity. Similarly, decreasing the equivalents of MBAA cross‐linker in the polymer formulation resulted in a marked reduction in selectivity (Figure 2b, right graph).

The significance of the polymeric structure in achieving chemoselectivity was evaluated through comparison with conventional small‐molecule catalysts. To this end, a series of bifunctional carboxylic acid–urea catalysts **5a–5e** bearing varied spatial arrangements was synthesized and subjected to the competitive epoxidation of **3a** and **3b**. As shown in Figure 2c, all small‐molecule catalysts exhibited reduced selectivity, albeit with a consistent preference for the ionic alkene **3a** (entries 8–12). Although selectivity might be improved through further structural tuning, such conventional strategies rely heavily on exhaustive catalyst screening, and the resulting catalysts often lack generality across a broad range of substrates.

**FIGURE 3:**
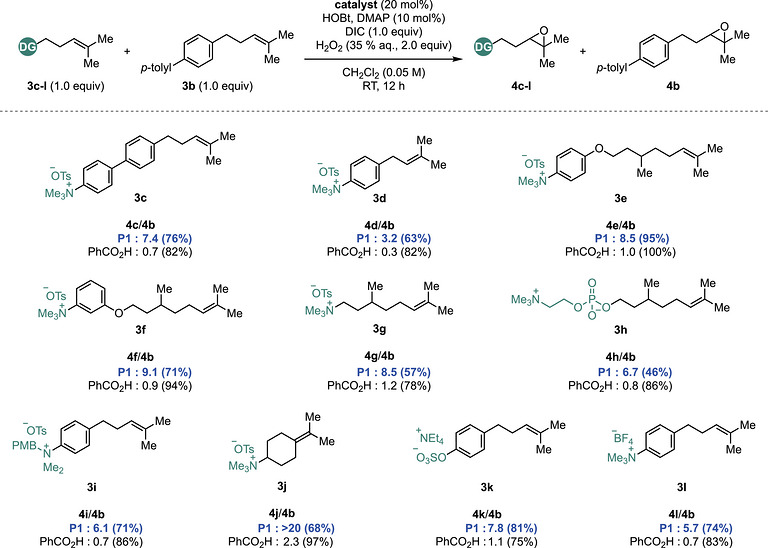
Substrate generality. Reaction conditions: 3c‐l (0.050 mmol), 3b (0.050 mmol), P1 or PhCO_2_H (20 mol%), HOBt (10 mol%), DMAP (10 mol%), DIC (0.050 mmol), H_2_O_2_ (35% aq., 0.10 mmol) in CH_2_Cl_2_ (1.0 mL) at room temperature (18–23 °C) for 12 h.

With the optimal catalyst in hand, we next sought to examine the substrate generality of this selective epoxidation under competitive conditions with **3b** (Figure 3). For comparison, the same competitive reactions were also conducted using benzoic acid as a catalyst. Consistent with our hypothesis, a range of substrates with varying spatial arrangements between the trimethylammonio group and alkene were selectively epoxidized, overriding intrinsic differences in olefin reactivity (**3c**‐**3f**). Likewise, epoxidation occurred preferentially for tetraalkylammonium salt **3g**. Notably, excellent chemoselectivity was also retained for phosphocholine **3h**, a characteristic subunit of phospholipids. The ammonium salt **3i** bearing a *p*‐methoxybenzyl (PMB) group also underwent epoxidation with high selectivity, presenting the opportunity for further transformation via deprotection to access the corresponding tertiary amine [65]. The successful epoxidation of substrate **3j**, featuring a conformationally constrained scaffold with spatially separated olefin and trimethylammonio moieties, further underscores the structural adaptability of the polymer catalyst. Moreover, this method was not limited to cationic alkenes: olefinic sulfonate **3k** was also a viable substrate, providing the epoxidation product with high selectivity. The diminished selectivity observed for alkene **3l** bearing a noncoordinating tetrafluoroborate anion highlights the critical role of anion binding in achieving high selectivity [66].

**FIGURE 4:**
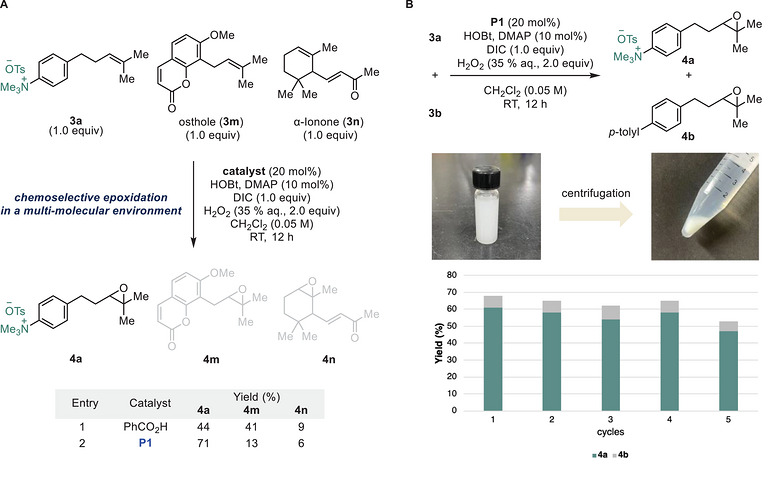
Synthetic applications of the polymer catalyst. (a) Chemoselective epoxidation in a multi‐molecular environment. (b) Recyclable usage of P1.

The performance of catalyst **P1** was further evaluated in a more complex mixture containing alkene‐bearing natural products (Figure 4a). An equimolar mixture of trimethylammonium **3a**, osthole (**3m**), and α‐ionone (**3n**) was subjected to the standard epoxidation conditions using benzoic acid as a catalyst, affording the corresponding epoxides **4a**, **4m**, and **4n** in 44%, 41%, and 9% yield, respectively (entry 1). In sharp contrast, the identical reaction catalyzed by polymer catalyst **P1** led to a substantial increase in the yield of the targeted product **4a** from 44% to 71%, underscoring the ability of **P1** to selectively recognize the ionic substrate even within a multi‐molecular environment (entry 2). To demonstrate the practical utility of the developed method, the reusability of polymer catalyst **P1** was examined in the competitive epoxidation of substrates **3a** and **3b** (Figure 4b). Notably, this heterogeneous catalyst was readily recovered by simple centrifugation and washing, and reused in at least five consecutive cycles without any significant loss in yield or selectivity.

**FIGURE 5:**
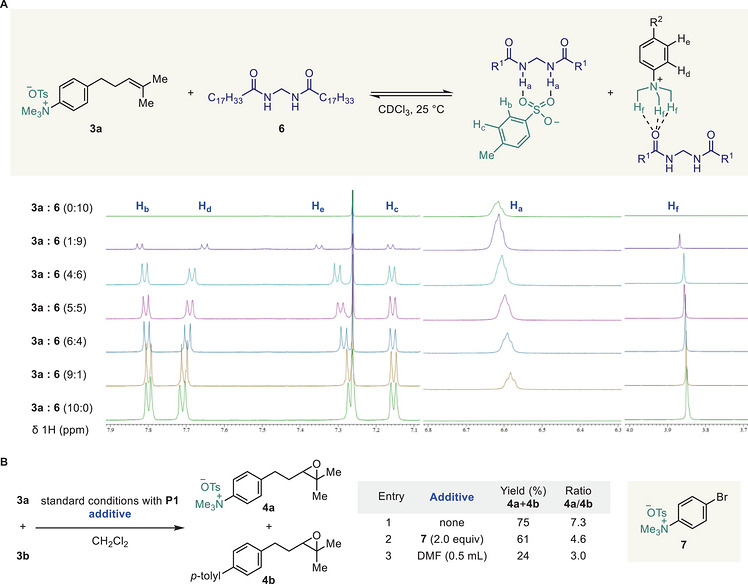
Mechanistic studies. a, ^1^H NMR spectra of the titration experiments using 3a and bis‐amide 6. b, Additive effect in the competitive reaction of 3a and 3b.

A number of additional experiments were performed to gain insight into the origin of the broad substrate generality and high selectivity exhibited by the polymer catalysts. We began by conducting ^1^H NMR titration experiments with varying ratios of ammonium tosylate **3a** and bis‐amide **6**, which is highly soluble in CDCl_3_, to probe the role of the cross‐linker in recognizing ionic substrates. As shown in Figure 5a, the anion binding of **6** was apparent as the presence of tosylate **3a** triggered an upfield shift of the NH signals (H_a_). Simultaneously, protons of the tosylate anion (H_b_ and H_c_) moved downfield. In addition, an incremental increase of the ratio of **6** led to the shift in the α‐protons adjacent to the quaternary ammonium moiety of **3a** (H_f_) and aromatic protons (H_d_ and H_e_), suggesting weak noncovalent interactions, such as C–H···O hydrogen bonding or electrostatic forces, between the quaternary ammonium cation and **6**. Similarly, titration experiments between urea and **3a** were performed, confirming that the urea moiety exhibits strong recognition toward **3a** (see the Supporting Information). We also performed a series of control experiments (Figure 5b). The addition of an excess amount of DMF to the competitive reaction between alkenes **3a** and **3b** led to a marked reduction in selectivity (**4a**/**4b** = 3.0, entry 2), likely due to the competitive hydrogen‐bonding of DMF with the ionic substrate **3a** and the polymer surface, thereby disrupting the key catalyst–substrate interactions. Similarly, the addition of 2 equivalents of ammonium tosylate **7** also diminished selectivity (**4a**/**4b** = 4.6, entry 3). Taken together, these observations provide compelling evidence that the MBAA cross‐linker engages in dual hydrogen‐bonding interactions—both as donor and acceptor—to effectively capture ionic substrates to achieve high selectivity.

**FIGURE 6:**
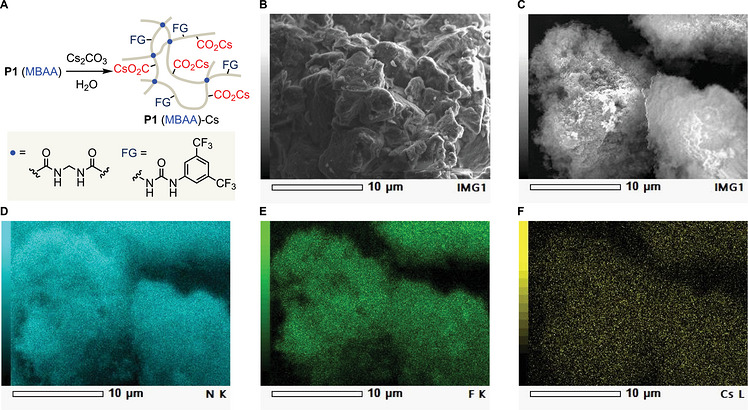
Structural characterization of P1. (a) Preparation of P1‐Cs. (b) SEM image of P1. (c) SEM image of P1‐Cs. (d–f) Energy‐dispersive spectroscopy mapping of P1‐Cs, N atom (light blue, d), F atom (green, e), and Cs atom (yellow, f), respectively.

To probe the features responsible for the observed generality toward diverse ionic alkenes, scanning electron microscopy (SEM) was conducted to reveal the microstructure of **P1**. The SEM images in Figure 6b showed irregular micrometer‐sized particles with dense and rough surfaces without a porous structure. To visualize the carboxylic acid moieties, cesium salt **P1**‐Cs was prepared by the treatment of **P1** with Cs_2_CO_3_ in H_2_O (Figure 6a). The elemental mapping analysis of **P1**‐Cs revealed uniform distributions of N, F, and Cs atoms, indicating that the urea, bis‐amide, and carboxylic acid functionalities are randomly dispersed across the polymer surface at the micro‐ and sub‐micrometer scales (Figure 6d–f). These findings align with our proposed structural model for random polymers and support the high structural adaptability exhibited by the polymer catalysts.

In summary, we have demonstrated that polymer‐based catalysts can reconcile high chemoselectivity with broad substrate generality—two characteristics often regarded as mutually exclusive in conventional small‐molecule catalysis. Through rational monomer design, a single heteropolymer functioned as a versatile epoxidation catalyst, delivering high selectivity across a structurally diverse array of ionic alkenes. Mechanistic investigations revealed that both the quaternary ammonium cation and its counteranion were captured on the polymer surface via hydrogen‐bonding interactions, thus enabling selective transformation. Scanning electron microscopy provided compelling evidence for a random distribution of functional residues across the polymer surface, a feature that likely underpins the observed substrate adaptability. We anticipate that this simple yet robust strategy will serve as a versatile platform for a broad spectrum of selective chemical transformations, particularly those demanding substrate generality.

## Conflicts of Interest

The authors declare no competing financial interests.

## Supporting information



The authors have cited additional references within the Supporting Information [67–76].
