# Supplementary material for: Harnessing Random Polymers as Chemoselective Catalysts With Structural Adaptability: Epoxidation of Olefinic Quaternary Ammonium Salts via Charge Recognition
Source: Chemistry. 2026 May 1;32(27):e71082. doi: 10.1002/chem.71082 (PMC13380382; doi:10.1002/chem.71082)

Supporting Information for:

Harnessing Random Polymers as Chemoselective Catalysts with Structural Adaptability: Epoxidation of Olefinic Quaternary Ammonium Salts via Charge Recognition

Yusei Fujii,[1] Kiyosei Takasu,[1]* Yusuke Kuroda[1,2]*

[1]Graduate School of Pharmaceutical Sciences, Kyoto University, Kyoto 606-8501, Japan
[2]The HAKUBI Center for Advanced Research, Kyoto University, Kyoto 606-8501, Japan

* Corresponding Author Email
takasu.kiyosei.6r@kyoto-u.ac.jp (K.T.)
kuroday@pharm.kyoto-u.ac.jp (Y.K.)

Table of Contents

1. General Considerations........1

2. Preparation of Substrates........2

3. Preparation of Authentic Samples of Epoxidation Products........10

4. Preparation of Epoxidation Catalysts........15

5. Competitive Epoxidation Reactions........20

6. ^{1}H NMR Titration Experiments........28

7. References........30

8. NMR Spectra........31

1. General Considerations

1.1. Solvents and reagents

N,N'-Methylenebisacrylamide (MBAA), 4-vinylbenzoic acid (VBA), 1-hydroxybenzotriazole monohydrate (HOBt·H_2O), and 2,2'-azobis(2,4-dimethylvaleronitrile) (V-65) were purchased from Tokyo Chemical Industry (TCI) and used as received. Hydrogen peroxide (35% aqueous solution) was purchased from Nacalai Tesque and used as received. *N,N'*-Diisopropylcarbodiimide (DIC) was obtained from Sigma-Aldrich and used as received. 4-Dimethylaminopyridine (DMAP) was purchased from Oakwood Chemical and used as received. *N,N*-Dimethylformamide (DMF) was purchased from FUJIFILM Wako Chemicals as "Super Dehydrated" and used as received. Dichloromethane (CH_2Cl_2) was purchased from Kanto Chemical Co., Inc. as "Dehydrated -Super^2-" and used as received. Unless otherwise noted, all other reagents were purchased from commercial suppliers and used as received.

1.2. Experimental procedures

Unless otherwise noted in the experimental procedures, reactions were carried out in flame or oven-dried glassware under a positive pressure of argon in anhydrous solvents using standard Schlenk techniques. Reaction progresses were monitored using thin-layer chromatography (TLC) on Merck TLC silica gel 60 F254 (0.25 mm) plates. Visualization of the developed plates was performed under UV-light (254 nm) irradiation, and then gently heated with $KMnO_4$ if necessary. Flash column chromatography was performed with a Yamazen Smart Flash EPCLC W-Prep 2XY automated flash chromatography system. Polymerization reactions were conducted using an Eppendorf ThermoMixer C. Centrifugation was carried out using an Eppendorf Centrifuge 5430.

1.3. Analytical instrumentation

NMR data were recorded on a JEOL JNM-LA 500 spectrometer (^{1}H NMR; 500 MHz, ^{13}C NMR; 126 MHz, ^{19}F NMR; 471 MHz) or a JEOL JNM-ECZ 600 spectrometer (^{1}H NMR; 600 MHz, ^{13}C NMR; 151 MHz, ^{31}P NMR; 243 MHz) typically at 20–23 °C. ^{1}H and ^{13}C NMR spectra were referenced using the residual solvent signal (^{1}H NMR; $CDCl_3$ at 7.26 ppm, ^{13}C NMR; $CDCl_3$ at 77.16 ppm). NMR data are reported as follows: chemical shift, multiplicity (s = singlet, d = doublet, t = triplet, q = quartet, quint = quintet, br = broad, m = multiplet), coupling constants (Hz), and integration. High-resolution mass spectra were obtained with Thermo Scientific Exactive Plus Orbitrap mass spectrometer for ESI mass spectrometry, and a JEOL JMS-SX102A or a JEOL JMS-MS700 mass spectrometer for EI mass spectrometry. Melting points were determined using an SRS OptiMelt MPA100 automated melting point system. Scanning electron microscopy (SEM) and energy-dispersive X-ray spectroscopy (EDX) analyses were carried out using a JEOL JSM-7900F microscope with JED-2300 after osmium coating. Image acquisition was performed with JEOL Analysis Station.

2. Preparation of Substrates

Representative procedure A

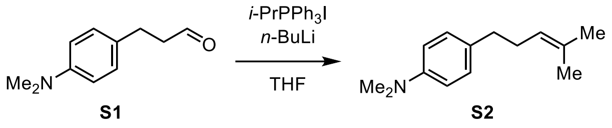

To a 50 mL round-bottom flask containing a solution of *i*-$PrPPh_3I$ (1.12 g, 2.60 mmol, 1.20 equiv) in THF (15 mL), *n*-BuLi (1.6 M in hexanes, 1.63 mL, 2.60 mmol, 1.20 equiv) was added dropwise over 15 min at 0 °C. The resulting mixture was stirred at 0 °C. After 1 h, aldehyde **S1** [67] (385 mg, 2.17 mmol, 1.00 equiv) was added, and the resulting mixture was then allowed to warm to room temperature. After 16 h, the mixture was diluted with hexanes, filtered through a pad of Celite and concentrated under reduced pressure. The crude residue was purified by column chromatography (SiO_2, 100/0 to 95/5 hexanes/EtOAc) to afford **S2** as a yellow oil (322 mg, 73%).

^{1}H NMR (500 MHz, $CDCl_3$): δ 7.07 (d, J = 8.6 Hz, 2H), 6.70 (d, J = 8.6 Hz, 2H), 5.18 (t, J = 7.2 Hz, 1H), 2.91 (s, 6H), 2.54 (t, J = 8.0 Hz, 2H), 2.25 (dt, J = 7.2, 8.0 Hz, 2H), 1.69 (s, 3H), 1.58 (s, 3H);

^{13}C NMR (151 MHz, $CDCl_3$): δ 149.1, 131.9, 130.9, 129.0, 124.2, 113.1, 41.1, 35.2, 30.5, 25.8, 17.8;

HRMS (ESI): Calc'd for $C_{14}H_{22}N$ $[M+H]^+$: 204.1747, found: 204.1745.

Representative procedure B

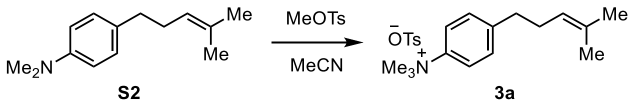

To a 50 mL round-bottom flask containing a solution of **S2** (322 mg, 1.59 mmol, 1.00 equiv) in MeCN (10 mL), MeOTs (264 µL, 1.75 mmol, 1.10 equiv) was added. The resulting mixture was stirred at 80 °C. After 3 h, the reaction mixture was allowed to cool to room temperature and concentrated under reduced pressure. The crude residue was dissolved in a minimum amount of $CHCl_3$, and **3a** was precipitated with Et_2O and collected by filtration as a white solid (384 mg, 62%).

Melting Point: 170–175 °C (decomp.);

^{1}H NMR (500 MHz, $CDCl_3$): δ 7.80 (d, J = 8.0 Hz, 2H), 7.70 (d, J = 8.6 Hz, 2H), 7.27 (d, J = 8.6 Hz, 2H), 7.15 (d, J = 8.0 Hz, 2H), 5.09 (t, J = 6.9 Hz, 1H), 3.85 (s, 9H), 2.63 (t, J = 7.7 Hz, 2H), 2.34 (s, 3H), 2.25 (dt, J = 6.9, 7.7 Hz, 2H), 1.67 (s, 3H), 1.54 (s, 3H);

^{13}C NMR (151 MHz, $CDCl_3$): δ 145.2, 145.1, 144.0, 139.3, 133.0, 130.5, 128.8, 126.0, 122.8, 119.6, 57.4, 35.3, 29.5, 25.7, 21.4, 17.8;

HRMS (ESI): Calc'd for $C_{15}H_{24}N$ $[M-OTs]^+$: 218.1903, found: 218.1904.

Preparation of 3b

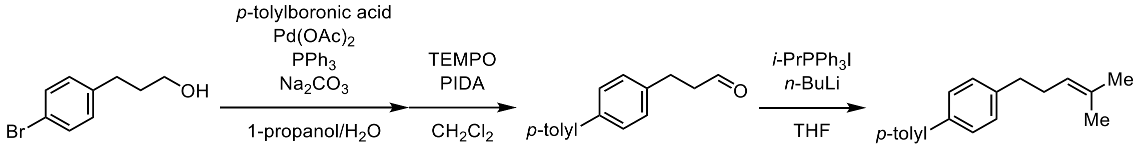

S3 S4 3b

To a 50 mL round-bottom flask containing **S3** (763 µL, 5.00 mmol, 1.00 equiv), *p*-tolylboronic acid (748 mg, 5.50 mmol, 1.10 equiv), $Pd(OAc)_2$ (6.7 mg, 0.030 mmol, 0.60 mol%), PPh_3 (24 mg, 0.090 mmol, 1.8 mol%) and Na_2CO_3 (583 mg, 5.50 mmol, 1.10 equiv), 1-propanol/H_2O (2/1, 15 mL) was added and the resulting mixture was stirred at 100 °C. After 18 h, the reaction mixture was cooled to room temperature and extracted with EtOAc (10 mL × 2). The combined organic layers were dried over Na_2SO_4, filtered and concentrated under reduced pressure to afford the corresponding crude alcohol, which was used directly in the next reaction without further purification. To a 50 mL round-bottom flask containing a solution of the crude alcohol in CH_2Cl_2 (20 mL), TEMPO (78 mg, 0.50 mmol, 10 mol%) and $PhI(OAc)_2$ (1.77 g, 5.50 mmol, 1.10 equiv) were added. The resulting mixture was stirred at room temperature. After 3 h, the reaction was quenched with sat. $Na_2S_2O_3$ aq. (10 mL), and phases were separated. The organic layer was washed with sat. Na_2CO_3 aq. (10 mL × 2), dried over Na_2SO_4, filtered, and concentrated under reduced pressure. The crude residue was purified by column chromatography (SiO_2, 100/0 to 90/10 hexanes/EtOAc) to afford **S4** as a white solid (1.00 g, 89% for 2 steps). **3b** was prepared using **S4** (551.8 mg, 2.46 mmol) following the representative procedure A. Purification by column chromatography (SiO_2, 100/0 hexanes/EtOAc) provided **3b** as a colorless oil (477 mg, 77%).

Rf: 0.50 (hexanes, UV);

^{1}H NMR (500 MHz, $CDCl_3$): δ 7.50 (d, J = 6.3 Hz, 2H), 7.48 (d, J = 6.3 Hz, 2H), 7.25 (d, J = 7.4 Hz, 2H), 7.24 (d, J = 7.4 Hz, 2H), 5.20 (t, J = 7.2 Hz, 1H), 2.67 (t, J = 7.7 Hz, 2H), 2.39 (s, 3H), 2.33 (dt, J = 7.2, 7.7 Hz, 2H), 1.70 (s, 3H), 1.59 (s, 3H);

^{13}C NMR (151 MHz, $CDCl_3$): δ 141.4, 138.7, 138.4, 136.8, 132.3, 129.5, 128.9, 126.94, 126.90, 123.9, 35.9, 30.2, 25.8, 21.2, 17.8;

HRMS (EI): Calc'd for $C_{19}H_{22}$ $[M]^+$: 250.1722, found: 250.1722.

Preparation of 3c

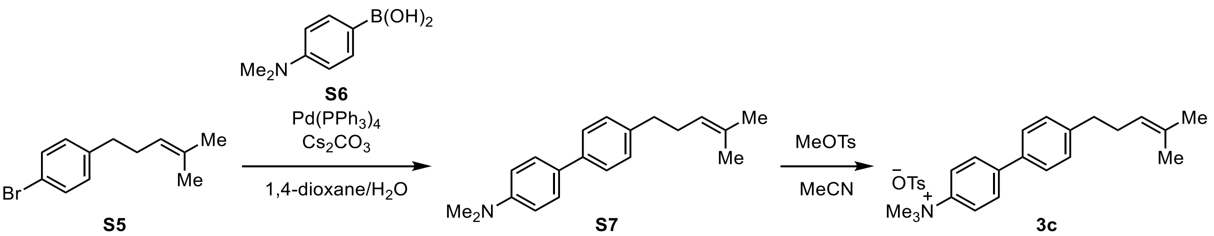

S5 S7 3c

To a 100 mL round-bottom flask containing **S5** (478 mg, 2.00 mmol, 1.00 equiv), **S6** (593 mg, 2.40 mmol, 1.20 equiv), $Pd(PPh_3)_4$ (116 mg, 0.100 mmol, 5.00 mol%) and Cs_2CO_3 (978 mg, 3.00 mmol, 1.50 equiv), 1,4-dioxane/H_2O (4/1, 20 mL) was added. The resulting mixture was stirred at 100 °C. After 18 h, the mixture was cooled to room temperature and extracted with $CHCl_3$ (10 mL × 2). The combined organic layers were dried over Na_2SO_4, filtered and concentrated under reduced pressure. The crude residue was purified by column

chromatography (SiO_2, 95/5 hexanes/EtOAc) to afford **S7** as a pale-yellow oil (444 mg, 80%). **3c** was prepared using **S7** (444 mg, 1.59 mmol) following the representative procedure B. Purification by recrystallization ($CHCl_3/Et_2O$) provided **3c** as a white solid (325 mg, 44%).

Melting Point: 193–198 °C (decomp.);

^{1}H NMR (500 MHz, $CDCl_3$): δ 7.87 (d, J = 9.2 Hz, 2H), 7.80 (d, J = 8.0 Hz, 2H), 7.61 (d, J = 9.2 Hz, 2H), 7.43 (d, J = 8.0 Hz, 2H), 7.28 (d, J = 8.0 Hz, 2H), 7.14 (d, J = 8.0 Hz, 2H), 5.17 (t, J = 7.2 Hz, 1H), 3.90 (s, 9H), 2.68 (t, J = 7.7 Hz, 2H), 2.34-2.30 (m, 2H), 2.31 (s, 3H), 1.69 (s, 3H), 1.57 (s, 3H);

^{13}C NMR (151 MHz, $CDCl_3$): δ 145.8, 143.8, 143.6, 143.3, 139.4, 135.8, 132.6, 129.3, 128.9, 128.8, 127.0, 126.0, 123.5, 120.0, 57.5, 35.8, 30.0, 25.8, 21.4, 17.8;

HRMS (ESI): Calc'd for $C_{21}H_{28}N$ $[M-OTs]^+$: 294.2216, found: 294.2218.

Preparation of 3d

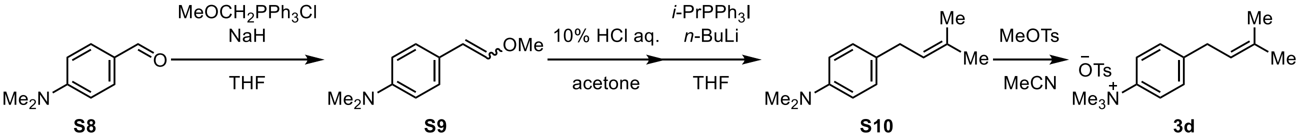

To a 100 mL round-bottom flask containing a solution of $MeOCH_2PPh_3Cl$ (5.14 g, 15.0 mmol, 1.50 equiv) in THF, NaH (60% oil, 600 mg, 15.0 mmol, 1.50 equiv) was added, and the resulting mixture was stirred at room temperature. After 2 h, **S8** (1.14 g, 10.0 mmol, 1.00 equiv) was added to the mixture, and the resulting mixture was heated to 100 °C. After 10 h, the reaction was cooled to room temperature, and quenched by sat. NH_4Cl aq. (20 mL) and the aqueous layer was extracted with EtOAc (20 mL × 2). The combined organic layers were dried over Na_2SO_4, filtered and concentrated under reduced pressure. The crude residue was purified by column chromatography (SiO_2, 90/10 hexanes/EtOAc) to afford **S9** as a pale-yellow oil (777 mg, 29%). To a 100 mL round-bottom flask containing a solution of **S9** (777 mg, 4.38 mmol, 1.00 equiv) in acetone (20 mL), 10% HCl aq. (5 mL) was added. The resulting mixture was heated to 70 °C. After 3 h, the reaction was cooled to room temperature, and quenched by sat. $NaHCO_3$ aq. (10 mL), and phases were separated. The aqueous layer was extracted with EtOAc (20 mL × 2). The combined organic layers were dried over Na_2SO_4, filtered and concentrated under reduced pressure to afford the corresponding crude aldehyde, which was used directly in the next reaction without further purification. **S10** was prepared using the crude aldehyde following the representative procedure A. Purification by column chromatography (SiO_2, 100/0 to 95/5 hexanes/EtOAc) provided **S10** as a yellow oil (467 mg, 61% for 2 steps). **3d** was prepared using **S10** (467 mg, 2.67 mmol) following the representative procedure B. Purification by recrystallization ($CHCl_3/Et_2O$) provided **3d** as a white solid (745 mg, 74%).

Melting Point: 145-150 °C (decomp.);

^{1}H NMR (600 MHz, $CDCl_3$): δ 7.76 (d, J = 7.6 Hz, 2H), 7.67 (d, J = 8.0 Hz, 2H), 7.25 (d, J = 7.6 Hz, 2H), 7.13 (d, J = 8.0 Hz, 2H), 5.22 (t, J = 6.9 Hz, 1H), 3.80 (s, 9H), 3.32 (d, J = 6.9 Hz, 2H), 2.33 (s, 3H), 1.74 (s, 3H), 1.68 (s, 3H);

^{13}C NMR (151 MHz, $CDCl_3$): δ 145.1, 144.8, 143.7, 139.4, 134.3, 130.4, 128.8, 126.0, 121.4, 119.5, 57.5, 33.6, 25.8, 21.4, 18.0;

HRMS (ESI): Calc'd for $C_{14}H_{22}N$ [M−OTs]$^+$: 204.1747, found: 204.1749.

Preparation of 3e

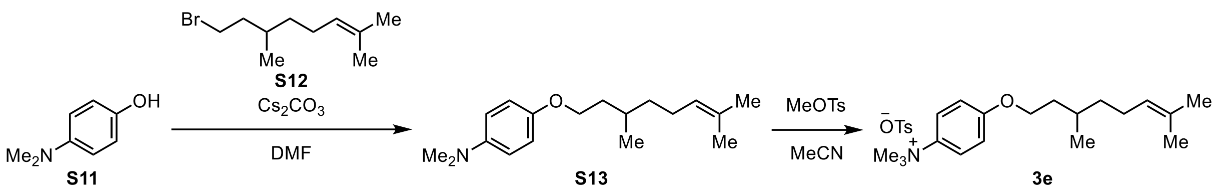

To a 50 mL round-bottom flask containing a solution of **S11** (274 mg, 2.00 mmol, 1.00 equiv) in DMF (10 mL), Cs_2CO_3 (978 mg, 3.00 mmol, 1.50 equiv) was added. The resulting mixture was heated to 60 °C. After 30 min, **S12** [68] (489 mg, 2.00 mmol, 1.00 equiv) was added dropwise over 10 min. After 12 h, the reaction was cooled to room temperature, and quenched with H_2O (10 mL). Then, hexanes were added to the reaction mixture, and the phases were separated. The aqueous layer was extracted with hexanes (10 mL × 2). The combined organic layers were dried over Na_2SO_4, filtered and concentrated under reduced pressure. The crude residue was purified by column chromatography (SiO_2, 90/10 hexanes/EtOAc) to afford **S13** as a pale-yellow oil (179 mg, 32%). **3e** was prepared using **S13** (179 mg, 0.65 mmol) following the representative procedure B. Purification by recrystallization ($CHCl_3$/Et_2O) provided **3e** as a white solid (215 mg, 72%).

Melting Point: 177–183 °C (decomp.);

^{1}H NMR (600 MHz, $CDCl_3$): δ 7.77 (d, *J* = 8.2 Hz, 2H), 7.70 (d, *J* = 9.0 Hz, 2H), 7.13 (d, *J* = 8.2 Hz, 2H), 6.88 (d, *J* = 9.0 Hz, 2H), 5.08 (t, *J* = 7.2 Hz, 1H), 3.98-3.91 (m, 2H), 3.80 (s, 9H), 2.32 (s, 3H), 2.04-1.94 (m, 2H), 1.69-1.63 (m, 1H), 1.67 (s, 3H), 1.59 (s, 3H), 1.59-1.54 (m, 1H), 1.40-1.34 (m, 1H), 1.24-1.18 (m, 1H), 0.93 (d, *J* = 6.9 Hz, 3H);

^{13}C NMR (151 MHz, $CDCl_3$): δ 160.0, 143.8, 139.8, 139.4, 131.5, 128.8, 126.0, 124.6, 121.1, 115.8, 67.0, 57.7, 37.1, 35.9, 29.5, 25.8, 25.5, 21.4, 19.5, 17.8;

HRMS (ESI): Calc'd for $C_{19}H_{32}ON$ [M−OTs]$^+$: 290.2478, found: 290.2482.

Preparation of 3f

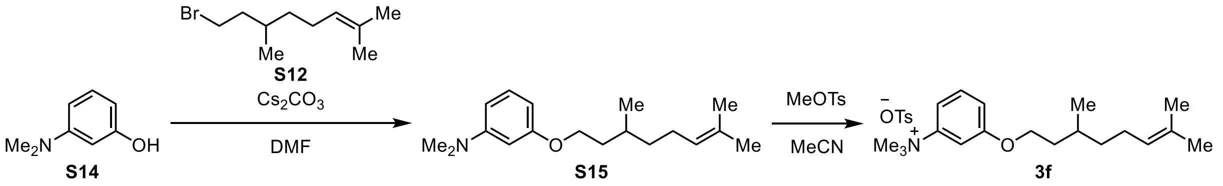

To a 50 mL round-bottom flask containing a solution of **S14** (274 mg, 2.00 mmol, 1.00 equiv) in DMF (10 mL), Cs_2CO_3 (978 mg, 3.00 mmol, 1.50 equiv) was added. The resulting mixture was heated to 60 °C. After 30 min, **S12** [68] (489 mg, 2.00 mmol, 1.00 equiv) was added dropwise over 10 min. After 12 h, the reaction was cooled to room temperature, and quenched with H_2O (10 mL), and phases were separated. The aqueous layer was extracted with hexanes (10 mL × 2). The combined organic layers were dried over Na_2SO_4, filtered and concentrated under reduced pressure. The crude residue was purified by column chromatography (SiO_2, 90/10 hexanes/EtOAc) to afford **S15** as a pale-yellow oil (510 mg, 93%). **3f** was prepared using **S15** (440 mg, 1.60 mmol) following the representative procedure B. Purification by recrystallization ($CHCl_3$/hexanes) provided **3f**

as a white solid (439 mg, 59%).

Melting Point: 73–78 °C (decomp.);

^{1}H NMR (500 MHz, $CDCl_3$): δ 7.79 (d, *J* = 8.0 Hz, 2H), 7.40 (t, *J* = 8.3 Hz, 1H), 7.30 (dd, *J* = 8.3, 2.6 Hz, 1H), 7.26 (s, 1H), 7.14 (d, *J* = 8.0 Hz, 2H), 6.98 (dd, *J* = 8.3, 2.6 Hz, 1H), 5.08 (t, *J* = 7.2 Hz, 1H), 4.05-3.98 (m, 2H), 3.85 (s, 9H), 2.34 (s, 3H), 2.04-1.91 (m, 2H), 1.81-1.76 (m, 1H), 1.67 (s, 3H), 1.65-1.62 (m, 1H), 1.60 (s, 3H), 1.56-1.48 (m, 1H), 1.39-1.15 (m, 2H), 0.92 (d, *J* = 6.9 Hz, 3H);

^{13}C NMR (151 MHz, $CDCl_3$): δ 160.6, 148.5, 143.8, 139.3, 131.4, 128.8, 126.0, 124.7, 116.1, 111.2, 106.7, 67.3, 57.4, 37.2, 35.9, 29.5, 25.8, 25.5, 21.4, 19.5, 17.8 (*One ^{13}C signal is overlapping with others*);

HRMS (ESI): Calc'd for $C_{19}H_{32}ON$ $[M{-}OTs]^+$: 290.2478, found: 290.2482.

Preparation of 3g

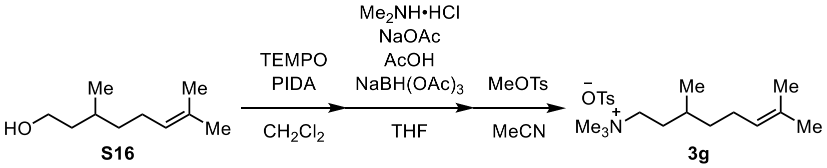

To a 100 mL round-bottom flask containing a solution of **S16** (898 μL, 5.00 mmol, 1.00 equiv) in CH_2Cl_2 (20 mL), TEMPO (78 mg, 0.50 mmol, 10 mol%) and $PhI(OAc)_2$ (1.77 g, 5.50 mmol, 1.10 equiv) were added. The resulting mixture was stirred at room temperature. After 3 h, the reaction was quenched with sat. $Na_2S_2O_3$ aq. (10 mL), and phases were separated. The organic layer was washed with sat. Na_2CO_3 aq. (10 mL × 2), dried over Na_2SO_4, filtered and concentrated under reduced pressure to afford the corresponding crude aldehyde, which was used directly in the next reaction without further purification. To a 100 mL round-bottom flask containing a solution of the crude aldehyde, $Me_2NH{\cdot}HCl$ (815 mg, 10.0 mmol, 2.00 equiv) and NaOAc (656 mg, 8.00 mmol, 1.60 equiv) in THF (20 mL), AcOH (172 μL, 3.00 mmol, 0.600 equiv) was added at 0 °C. The resulting mixture was stirred at 0 °C. After 1 h, $NaBH(OAc)_3$ (2.33 g, 11.0 mmol, 2.20 equiv) was added, and the resulting mixture was allowed to warm to room temperature. After 12 h, the reaction was quenched by 10% HCl aq. (20 mL), and phases were separated. The aqueous layer was washed with EtOAc (20 mL × 2). The aqueous layer was then neutralized with 1 M NaOH aq. (10 mL) and extracted with EtOAc (20 mL × 2). The combined organic layers were dried over Na_2SO_4, filtered and concentrated under reduced pressure to afford the corresponding crude amine, which was used directly in the next reaction without further purification. **3g** was prepared using the crude amine (649 mg, ca. 3.54 mmol) following the representative procedure B. Purification by recrystallization ($CHCl_3$/Et_2O) provided **3g** as a white solid (953 mg, 52% for 3 steps).

Melting Point: 165–170 °C (decomp.);

^{1}H NMR (500 MHz, $CDCl_3$): δ 7.74 (d, *J* = 7.8 Hz, 2H), 7.13 (d, *J* = 7.8 Hz, 2H), 5.01 (t, *J* = 6.6 Hz, 1H), 3.47-3.42 (m, 1H), 3.37-3.30 (m, 1H), 3.33 (s, 9H), 2.32 (s, 3H), 1.99-1.86 (m, 2H), 1.69-1.63 (m, 1H), 1.66 (s, 3H), 1.57 (s, 3H), 1.50-1.40 (m, 2H), 1.33-1.27 (m, 1H), 1.18-1.11 (m, 1H), 0.88 (dd, *J* = 6.3, 3.4 Hz, 3H);

^{13}C NMR (151 MHz, $CDCl_3$): δ 144.1, 139.3, 131.8, 128.7, 125.9, 124.1, 65.5, 52.9, 36.8, 30.3, 29.9, 25.8, 25.3, 21.4, 19.1, 17.8;

HRMS (ESI): Calc'd for $C_{13}H_{28}N$ $[M{-}OTs]^+$: 198.2216, found: 198.2213.

Preparation of 3h

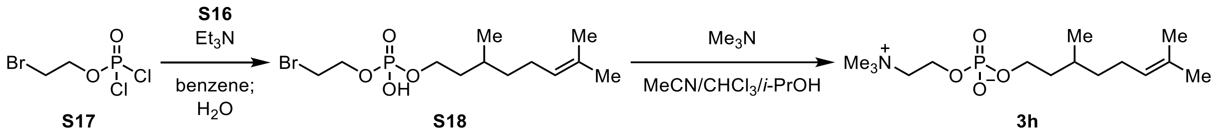

To a 30 mL round-bottom flask containing a solution of **S17** [69] (726 mg, 3.00 mmol, 1.50 equiv) in benzene (10 mL), Et_3N (416 µL, 3.00 mmol, 1.50 equiv) and **S16** (363 µL, 2.00 mmol, 1.00 equiv) were added. The resulting mixture was stirred at room temperature. After 18 h, volatiles were removed under reduced pressure and H_2O (10 mL) was added, and the resulting mixture was stirred at room temperature. After 1 h, the aqueous layer was extracted with $CHCl_3$ (20 mL × 2). The combined organic layers were dried over Na_2SO_4, filtered and concentrated under reduced pressure to afford the corresponding crude phosphate diester **S18**, which was used directly in the next reaction without further purification. To a 50 mL round-bottom flask containing a solution of **S18** in CH_3CN/*i*-PrOH/$CHCl_3$ (5/5/3, 13 mL), Me_3N aq. (4.3 M, 2.3 mL, 10 mmol, 5.0 equiv) was added. The resulting mixture was heated to 60 °C. After 18 h, the reaction mixture was concentrated under reduced pressure. The crude residue was purified by column chromatography (SiO_2, 80/20/0 to 60/33/7 $CHCl_3$/MeOH/H_2O) to afford **3h** as a white solid (229 mg, 36% for 2 steps).

Rf: 0.10 (60/33/7 $CHCl_3$/MeOH/H_2O, $KMnO_4$)

Melting Point: 83–88 °C (decomp.);

^{1}H NMR (500 MHz, $CDCl_3$): δ 5.06 (t, *J* = 6.9 Hz, 1H), 4.27 (brs, 2H), 3.87-3.81 (m, 4H), 3.39 (s, 9H), 2.01-1.86 (m, 2H), 1.66 (s, 3H), 1.64-1.60 (m, 1H), 1.57 (s, 3H), 1.54-1.51 (m, 1H), 1.42-1.36 (m, 1H), 1.34-1.27 (m, 1H), 1.16-1.09 (m, 1H), 0.86 (d, *J* = 6.9 Hz, 3H);

^{13}C NMR (151 MHz, $CDCl_3$): δ 131.2, 124.7, 66.2 (d, $J_{C–P}$ = 5.8 Hz), 64.0 (d, $J_{C–P}$ = 5.8 Hz), 59.3 (d, $J_{C–P}$ = 4.3 Hz), 54.3, 38.1 (d, $J_{C–P}$ = 5.8 Hz), 37.3, 29.5, 25.8, 25.5, 19.4, 17.7;

^{31}P NMR (243 MHz, $CDCl_3$): δ −0.8;

HRMS (ESI): Calc'd for $C_{15}H_{32}O_4NNaP$ $[M+Na]^+$: 344.1961, found: 344.1972.

Preparation of 3i

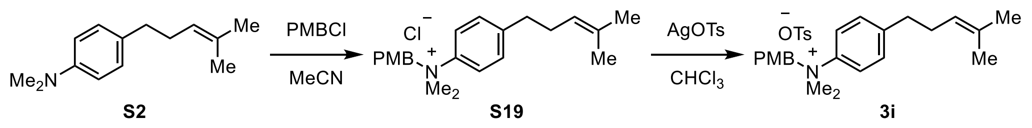

To a 50 mL round-bottom flask containing a solution of **S2** (203 mg, 1.00 mmol, 1.00 equiv) in MeCN (10 mL), *p*-methoxybenzyl chloride (149 µL, 1.10 mmol, 1.10 equiv) was added. The resulting mixture was stirred at room temperature. After 12 h, the reaction mixture was filtered and concentrated under reduced pressure to afford the corresponding chloride salt **S19**, which was used directly in the next reaction without further purification. To a 50 mL round-bottom flask containing a solution of **S19** in $CHCl_3$ (10 mL), AgOTs (1.40 g, 5.00 mmol, 5.00 equiv) was added. The resulting mixture was stirred at room temperature. After 12 h, the reaction mixture was filtered and concentrated under reduced pressure. The crude residue was dissolved in $CHCl_3$ and recrystallized upon slow addition of Et_2O to afford **3i** as a white solid (331 mg, 67% for 2 steps).

Melting Point: 127–132 °C (decomp.);

^{1}H NMR (500 MHz, $CDCl_3$): δ 7.88 (d, J = 8.6 Hz, 2H), 7.42 (d, J = 8.6 Hz, 2H), 7.25 (d, J = 7.4 Hz, 2H), 7.16 (d, J = 7.4 Hz, 2H), 6.93 (d, J = 8.6 Hz, 2H), 6.65 (d, J = 8.6 Hz, 2H), 5.21 (s, 2H), 5.07 (t, J = 7.2 Hz, 1H), 3.75 (s, 6H), 3.72 (s, 3H), 2.65 (t, J = 7.4 Hz, 2H), 2.34 (s, 3H), 2.27 (q, J = 7.3 Hz, 2H), 1.68 (s, 3H), 1.55 (s, 3H);
^{13}C NMR (151 MHz, $CDCl_3$): δ 161.0, 145.2, 144.0, 142.1, 139.3, 134.3, 133.0, 130.2, 128.8, 126.2, 122.8, 121.2, 119.8, 113.9, 73.3, 55.3, 53.0, 35.3, 29.5, 25.8, 21.4, 17.8;
HRMS (ESI): Calc'd for $C_{22}H_{30}ON$ $[M-OTs]^+$: 324.2322, found: 324.2325.

Preparation of 3j

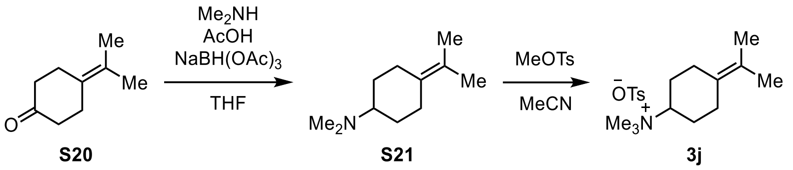

To a 100 mL round-bottom flask containing a solution of **S20** [70] (276 mg, 2.00 mmol, 1.00 equiv), Me_2NH (2.0 M in THF, 1.10 mL, 2.20 mmol, 1.10 equiv) in THF (10 mL), AcOH (11 µL, 0.20 mmol, 0.10 equiv) was added. The resulting mixture was stirred at room temperature. After 1 h, $NaBH(OAc)_3$ (2.33 g, 11.0 mmol, 5.50 equiv) was added. After 12 h, the reaction was quenched by 10% HCl aq. (20 mL), and the aqueous layer was washed with EtOAc (20 mL × 2). The aqueous layer was neutralized with 1 M NaOH aq. (10 mL) and extracted with EtOAc (20 mL × 2). The combined organic layers were dried over Na_2SO_4, filtered and concentrated under reduced pressure to afford the corresponding crude amine **S21**, which was used directly in the next reaction without further purification. **3j** was prepared using the crude amine **S21** (195 mg, ca. 1.16 mmol) following the representative procedure B. Purification by recrystallization ($CHCl_3/Et_2O$) provided **3j** as a white solid (383 mg, 54% for 2 steps).
Melting Point: 190–195 °C (decomp.);
^{1}H NMR (500 MHz, $CDCl_3$): δ 7.77 (d, J = 7.4 Hz, 2H), 7.14 (d, J = 7.4 Hz, 2H), 3.61 (tt, J = 12.0, 3.2 Hz, 1H), 3.27 (s, 9H), 2.78 (d, J = 14.3 Hz, 2H), 2.32 (s, 3H), 2.24 (d, J = 11.5 Hz, 2H), 1.83-1.76 (m, 2H), 1.63 (s, 6H), 1.38-1.32 (m, 2H);
^{13}C NMR (151 MHz, $CDCl_3$): δ 144.0, 139.3, 128.7, 126.5, 125.9, 124.0, 74.1, 51.0, 27.6, 26.9, 21.4, 20.1;
HRMS (ESI): Calc'd for $C_{12}H_{24}N$ $[M-OTs]^+$: 182.1903, found: 182.1902.

Preparation of 3k

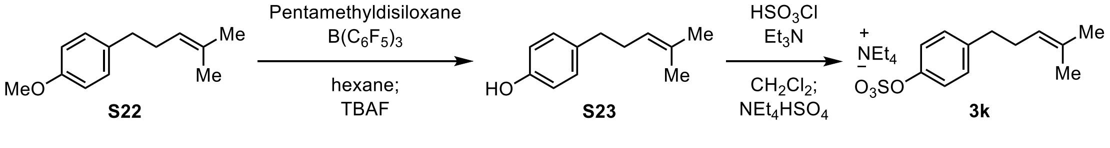

To a 50 mL round-bottom flask containing a solution of **S22** [71] (381 mg, 2.00 mmol, 1.00 equiv) in hexanes (10 mL), pentamethyldisiloxane (429 µL, 2.20 mmol, 1.10 equiv) was added. The resulting mixture was stirred at room temperature. After 1 min, $B(C_6F_5)_3$ (10 mg, 0.020 mmol, 1.0 mol%) was added. After 2 h, TBAF (1 M in THF, 3.0 mL, 3.0 mmol, 1.5 equiv) was added. After 30 min, the reaction was quenched by sat. NH_4Cl aq. (5

mL), and the aqueous layer was extracted with EtOAc (20 mL × 2). The combined organic layers were dried over Na_2SO_4, filtered and concentrated under reduced pressure. The crude residue was purified by column chromatography (SiO_2, 90/10 to 85/15 hexanes/EtOAc) to afford **S23** as a colorless oil (282 mg, 80%). To a 30 mL round-bottom flask containing a solution of **S23** (353 mg, 2.00 mmol, 1.00 equiv) in CH_2Cl_2 (5 mL), Et_3N (2.77 mL, 20.0 mmol, 10.0 equiv) and chlorosulfonic acid (132 µL, 2.00 mmol, 10.0 equiv) were added at 0 °C. The reaction mixture was then allowed to warm to room temperature. After 1 h, the reaction mixture was quenched by 10% NaOH aq. (2 mL), and the aqueous layer was washed with CH_2Cl_2 (10 mL × 2). To the aqueous layer was added NEt_4HSO_4 (409 mg, 1.80 mmol, 0.90 equiv), and extracted with CH_2Cl_2 (10 ml × 3). The combined organic layers were dried over Na_2SO_4, filtered and concentrated under reduced pressure. The crude residue was dissolved in $CHCl_3$ and recrystallized upon slow addition of Et_2O to afford **3k** as a white solid (384 mg, 62%).

Melting Point: 88–91 °C;

^{1}H NMR (500 MHz, $CDCl_3$): δ 7.27 (d, *J* = 8.6 Hz, 2H), 7.08 (d, *J* = 8.6 Hz, 2H), 5.14 (t, *J* = 6.9 Hz, 1H), 3.29 (q, *J* = 7.3 Hz, 8H), 2.55 (t, *J* = 7.7 Hz, 2H), 2.23 (dt, *J* = 6.9, 7.7 Hz, 2H), 1.67 (s, 3H), 1.56 (s, 3H), 1.29 (t, *J* = 7.3 Hz, 12H);

^{13}C NMR (151 MHz, $CDCl_3$): δ 151.5, 137.9, 132.2, 128.9, 123.8, 121.1, 52.4, 35.6, 30.3, 25.8, 17.8, 7.6;

HRMS (ESI): Calc'd for $C_{12}H_{15}O_4S$ $[M-NEt_4]^-$: 255.0686, found: 255.0692.

Preparation of 3l

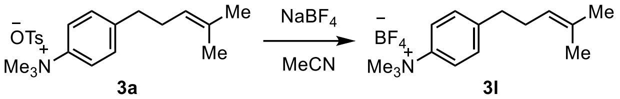

To a 30 mL round-bottom flask containing a solution of **3a** (77.9 mg, 0.200 mmol, 1.00 equiv) in MeCN (5 mL), $NaBF_4$ (110 mg, 1.00 mmol, 5.00 equiv) was added. The resulting mixture was stirred at room temperature. After 3 h, the reaction mixture was filtered and concentrated under reduced pressure to afford **3l** as a white solid (60.5 mg, 99%).

Melting Point: 126–129 °C;

^{1}H NMR (600 MHz, $CDCl_3$): δ 7.64 (d, *J* = 9.0 Hz, 2H), 7.35 (d, *J* = 9.0 Hz, 2H), 5.08 (t, *J* = 7.2 Hz, 1H), 3.65 (s, 9H), 2.64 (t, *J* = 7.9 Hz, 2H), 2.26 (dt, *J* = 7.2, 7.9 Hz, 2H), 1.66 (s, 3H), 1.53 (s, 3H);

^{13}C NMR (151 MHz, $CDCl_3$): δ 145.5, 144.8, 133.1, 130.7, 122.8, 119.3, 57.3, 35.3, 29.5, 25.7, 17.8;

^{19}F NMR (471 MHz, $CDCl_3$): δ −150.6;

HRMS (ESI): Calc'd for $C_{15}H_{24}N$ $[M-BF_4]^+$: 218.1903, found: 218.1904.

3. Preparation of Authentic Samples of Epoxidation Products

Representative procedure C

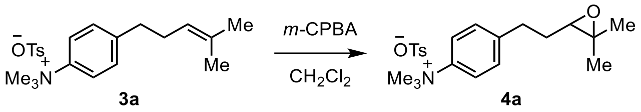

To a 30 mL round-bottom flask containing alkene **3a** (39.5 mg, 0.10 mmol, 1.00 equiv) in CH_2Cl_2 (2.0 mL), *m*-CPBA (ca. 30% water, 27.1 mg, 0.110 mmol, 1.10 equiv) was added at 0 °C, and the resulting mixture was stirred at 0 °C. After 1 h, Et_2O (10 mL) was added, and the resulting suspension was then filtered to afford epoxide **4a** as a white solid (30.8 mg, 76%).

Melting Point: 176–181 °C (decomp.);

^{1}H NMR (600 MHz, $CDCl_3$): δ 7.77-7.75 (m, 4H), 7.24 (d, *J* = 6.9 Hz, 2H), 7.12 (d, *J* = 8.3 Hz, 2H), 3.81 (s, 9H), 2.82-2.78 (m, 1H), 2.73-2.68 (m, 2H), 2.32 (s, 3H), 1.84-1.79 (m, 1H), 1.76-1.70 (m, 1H), 1.27 (s, 3H), 1.19 (s, 3H);

^{13}C NMR (151 MHz, $CDCl_3$): δ 145.5, 144.2, 144.0, 139.4, 130.5, 128.8, 126.0, 119.9, 63.4, 58.6, 57.3, 32.1, 30.3, 24.9, 21.4, 18.8;

HRMS (ESI): Calc'd for $C_{15}H_{24}ON$ $[M-OTs]^+$: 234.1852, found: 234.1855.

Preparation of 4b

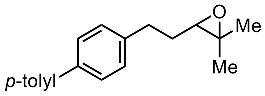

4b

To a 10 mL round-bottom flask containing alkene **3b** (25.0 mg, 0.10 mmol, 1.00 equiv) in CH_2Cl_2 (2.0 mL), *m*-CPBA (ca. 30% water, 27.1 mg, 0.110 mmol, 1.10 equiv) was added at 0 °C. The resulting mixture was stirred at 0 °C. After 1 h, the reaction was quenched by sat. $Na_2S_2O_3$ aq. (10 mL), and phases were separated. The aqueous phase was extracted by $CHCl_3$ (10 mL × 2), dried over Na_2SO_4, filtered, and concentrated under reduced pressure. The crude residue was purified by column chromatography (SiO_2, 100/0 to 90/10 hexanes/EtOAc) to afford **4b** as a colorless oil (23.7 mg, 89%).

Rf: 0.15 (90/10 hexanes/EtOAc, UV);

^{1}H NMR (600 MHz, $CDCl_3$): δ 7.53 (d, *J* = 8.3 Hz, 2H), 7.49 (d, *J* = 8.3 Hz, 2H), 7.27 (d, *J* = 8.3 Hz, 2H), 7.25 (d, *J* = 8.3 Hz, 2H), 2.91-2.87 (m, 1H), 2.81-2.74 (m, 2H), 2.40 (s, 3H), 1.96-1.91 (m, 1H), 1.88-1.82 (m, 1H), 1.30 (s, 3H), 1.17 (s, 3H);

^{13}C NMR (151 MHz, $CDCl_3$): δ 140.3, 139.0, 138.2, 136.9, 129.6, 129.0, 127.0, 126.9, 64.0, 58.7, 32.5, 30.9, 24.9, 21.2, 18.8;

HRMS (ESI): Calc'd for $C_{19}H_{22}ONa$ $[M+Na]^+$: 289.1563, found: 289.1562.

Preparation of 4c

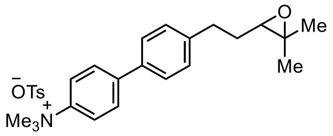

4c

Following the representative procedure C using alkene **3c** (46.6 mg, 0.100 mmol), epoxide **4c** (38.9 mg, 81%) was obtained as a white solid.

Melting Point: 210–220 °C (decomp.);

^{1}H NMR (500 MHz, $CDCl_3$): δ 7.90 (d, *J* = 8.6 Hz, 2H), 7.81 (d, *J* = 8.0 Hz, 2H), 7.62 (d, *J* = 8.6 Hz, 2H), 7.45 (d, *J* = 8.0 Hz, 2H), 7.31 (d, *J* = 8.0 Hz, 2H), 7.15 (d, *J* = 8.0 Hz, 2H), 3.93 (s, 9H), 2.91-2.87 (m, 1H), 2.81-2.75 (m, 2H), 2.32 (s, 3H), 1.91-1.85 (m, 2H), 1.29 (s, 3H), 1.18 (s, 3H);

^{13}C NMR (151 MHz, $CDCl_3$): δ 146.2, 144.0, 142.9, 142.0, 139.4, 136.4, 129.3, 128.8, 128.7, 127.2, 126.0, 120.4, 63.8, 58.7, 57.3, 32.5, 30.8, 24.9, 21.4, 18.8;

HRMS (ESI): Calc'd for $C_{21}H_{28}ON$ [M−OTs]$^+$: 310.2165, found: 310.2169.

Preparation of 4d

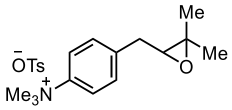

4d

Following the representative procedure C using alkene **3d** (37.6 mg, 0.100 mmol), epoxide **4d** (32.3 mg, 82%) was obtained as a white solid.

Melting Point: 175–180 °C (decomp.);

^{1}H NMR (500 MHz, $CDCl_3$): δ 7.80 (d, *J* = 8.6 Hz, 2H), 7.78 (d, *J* = 8.0 Hz, 2H), 7.34 (d, *J* = 8.6 Hz, 2H), 7.15 (d, *J* = 8.0 Hz, 2H), 3.84 (s, 9H), 2.90-2.79 (m, 3H), 2.34 (s, 3H), 1.37 (s, 3H), 1.32 (s, 3H);

^{13}C NMR (151 MHz, $CDCl_3$): δ 145.9, 144.0, 140.9, 139.4, 130.8, 128.8, 126.0, 120.3, 63.7, 58.9, 57.3, 34.7, 24.8, 21.4, 19.0;

HRMS (ESI): Calc'd for $C_{14}H_{22}ON$ [M−OTs]$^+$: 220.1696, found: 220.1695.

Preparation of 4e

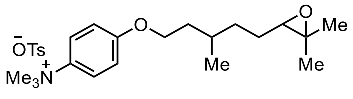

4e

Following the representative procedure C using alkene **3e** (46.2 mg, 0.100 mmol), epoxide **4e** (39.5 mg, 83%, dr = 1:1) was obtained as a white solid.

Melting Point: 167–172 °C (decomp.);

^{1}H NMR (500 MHz, $CDCl_3$): δ 7.79 (d, *J* = 8.0 Hz, 2H), 7.72 (d, *J* = 9.5 Hz, 2H), 7.15 (d, *J* = 8.0 Hz, 2H), 6.91 (d, *J* = 9.5 Hz, 2H), 4.01-3.93 (m, 2H), 3.83 (s, 9H), 2.70 (t, *J* = 6.0 Hz, 1H), 2.34 (s, 3H), 1.87-1.80 (m, 1H),

1.74-1.65 (m, 1H), 1.64-1.39 (m, 5H), 1.31 (s, 3H), 1.27 (s, 3H), 0.96 (d, *J* = 6.9 Hz, 3H);
^{13}C NMR (151 MHz, $CDCl_3$): δ 159.9, 143.9, 139.9, 139.3, 128.8, 126.0, 121.2, 115.8, [66.8, 66.7], [64.6, 64.5], [58.4, 58.3], 57.6, [36.0, 35.8], [33.8, 33.7], [29.8, 29.7], [26.6, 26.3], 25.0, 21.4, [19.5, 19.4], [18.84, 18.80] (*Signals in square brackets correspond to diastereomeric pairs*);
HRMS (ESI): Calc'd for $C_{19}H_{32}O_2N$ $[M{-}OTs]^+$: 306.2428, found: 306.2423.

Preparation of 4f

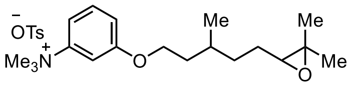

4f

Following the representative procedure C using alkene **3f** (46.2 mg, 0.100 mmol), epoxide **4f** (47.3 mg, 99%, dr = 1:1) was obtained as a white solid.
Melting Point: 46–51 °C (decomp.);
^{1}H NMR (600 MHz, $CDCl_3$): δ 7.78 (d, *J* = 7.6 Hz, 2H), 7.41 (t, *J* = 8.3 Hz, 1H), 7.32-7.30 (m, 1H), 7.28 (dd, *J* = 8.3, 2.8 Hz, 1H), 7.14 (d, *J* = 7.6 Hz, 2H), 6.98 (td, *J* = 5.2, 2.8 Hz, 1H), 4.07-4.02 (m, 2H), 3.86 (s, 9H), 2.69 (t, *J* = 6.2 Hz, 1H), 2.33 (s, 3H), 1.82-1.39 (m, 7H), 1.29 (s, 3H), 1.26 (s, 3H), 0.93 (d, *J* = 5.5 Hz, 3H);
^{13}C NMR (151 MHz, $CDCl_3$): δ 160.6, 148.4, 143.7, 139.4, 131.5, 128.7, 126.0, 116.2, 110.9, 106.9, [67.3, 67.1], [64.7, 64.6], [58.5, 58.4], 57.5, [36.0, 35.7], [33.8, 33.6], [29.8, 29.6], [26.6, 26.2], 25.0, 21.4, [19.5, 19.4], [18.9, 18.8] (*Signals in square brackets correspond to diastereomeric pairs*);
HRMS (ESI): Calc'd for $C_{19}H_{32}O_2N$ $[M{-}OTs]^+$: 306.2428, found: 306.2423.

Preparation of 4g

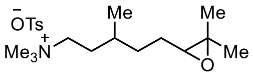

4g

Following the representative procedure C using alkene **3g** (37.0 mg, 0.100 mmol), epoxide **4g** (32.2 mg, 84%, dr = 1:1) was obtained as a white solid.
Melting Point: 113–118 °C (decomp.);
^{1}H NMR (600 MHz, $CDCl_3$): δ 7.67 (d, *J* = 7.6 Hz, 2H), 7.10 (d, *J* = 7.6 Hz, 2H), 3.35-3.23 (m, 2H), 3.15 (s, 9H), 2.59 (t, *J* = 5.5 Hz, 1H), 2.28 (s, 3H), 1.65-1.27 (m, 7H), 1.23 (s, 3H), 1.19 (s, 3H), 0.80 (d, *J* = 5.9 Hz, 3H);
^{13}C NMR (151 MHz, $CDCl_3$): δ 143.5, 139.6, 128.8, 125.9, [65.3, 65.2], [64.4, 64.3], [58.6, 58.4], 53.1, [33.5, 33.3], [30.7, 30.4], [29.7, 29.4], [26.4, 25.9], 24.9, 21.3, [19.14, 19.06], [18.8, 18.7] (*Signals in square brackets correspond to diastereomeric pairs*);
HRMS (ESI): Calc'd for $C_{13}H_{28}ON$ $[M{-}OTs]^+$: 214.2165, found: 214.2166.

Preparation of 4h

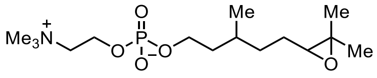

4h

Following the representative procedure C using alkene **3h** (32.1 mg, 0.100 mmol), epoxide **4h** (26.4 mg, 78%, dr = 1:1) was obtained as a white solid.

Melting Point: 65–70 °C (decomp.);

^{1}H NMR (500 MHz, $CDCl_3$): δ 4.22 (brs, 2H), 3.82-3.75 (m, 4H), 3.35 (s, 9H), 2.64-2.61 (m, 1H), 1.64-1.43 (m, 4H), 1.39-1.31 (m, 2H), 1.240 (s, 1.5H), 1.236 (s, 1.5H), 1.20 (s, 3H), 1.18-1.10 (m, 1H), 0.840 (d, J = 6.5 Hz, 1.5H), 0.834 (d, J = 6.5 Hz, 1.5H);

^{13}C NMR (151 MHz, $CDCl_3$): δ 66.3 (d, J = 5.7 Hz), 64.7, 64.5, 63.7 (d, $J_{C–P}$ = 5.7 Hz), 63.6 (d, $J_{C–P}$ = 5.9 Hz), 59.3 (d, $J_{C–P}$ = 4.4 Hz), 58.5, 58.3, 54.3, 38.2 (d, $J_{C–P}$ = 7.2 Hz), 37.9 (d, $J_{C–P}$ = 7.2 Hz), 33.8, 33.7, 29.6, 29.4, 26.6, 26.2, 25.0, 19.5, 19.4, 18.84, 18.82 (*Four 13C signals are overlapping with others*);

^{31}P NMR (243 MHz, $CDCl_3$) δ −0.6;

HRMS (ESI): Calc'd for $C_{15}H_{32}O_5NNaP$ $[M+Na]^+$: 360.1910, found: 360.1923.

Preparation of 4i

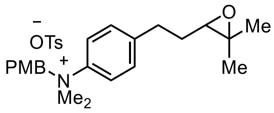

4i

Following the representative procedure C using alkene **3i** (49.6 mg, 0.100 mmol), epoxide **4i** (41.1 mg, 80%) was obtained as a white solid.

Melting Point: 87–92 °C (decomp.);

^{1}H NMR (500 MHz, $CDCl_3$): δ 7.88 (d, J = 8.0 Hz, 2H), 7.47 (d, J = 8.6 Hz, 2H), 7.31 (d, J = 8.6 Hz, 2H), 7.17 (d, J = 8.0 Hz, 2H), 6.95 (d, J = 8.6 Hz, 2H), 6.66 (d, J = 8.6 Hz, 2H), 5.21 (s, 2H), 3.76 (s, 6H), 3.72 (s, 3H), 2.89-2.75 (m, 2H), 2.71 (dd, J = 7.7, 4.9 Hz, 1H), 2.34 (s, 3H), 1.92-1.85 (m, 1H), 1.80-1.73 (m, 1H), 1.30 (s, 3H), 1.23 (s, 3H);

^{13}C NMR (151 MHz, $CDCl_3$): δ 160.9, 144.0, 143.9, 142.4, 139.3, 134.3, 130.0, 128.8, 126.1, 121.7, 119.9, 113.8, 72.9, 63.4, 58.6, 55.2, 53.0, 52.9, 32.1, 30.3, 24.9, 21.4, 18.8;

HRMS (ESI): Calc'd for $C_{22}H_{30}O_2N$ $[M−OTs]^+$: 340.2271, found: 340.2277.

Preparation of 4j

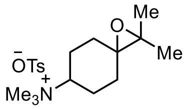

4j

Following the representative procedure C using alkene **3j** (35.4 mg, 0.100 mmol), epoxide **4j** (32.0 mg, 71%,

dr = 7.2:1) was obtained as a white solid.

Melting Point: 198–203 °C (decomp.);

^{1}H NMR (500 MHz, $CDCl_3$): δ 7.78 (d, *J* = 8.0 Hz, 2H), 7.15 (d, *J* = 8.0 Hz, 2H), 4.26-4.20 (m, 1H), 3.28 (s, 9H), 2.33 (s, 3H), 2.30-2.28 (m, 2H), 2.06-2.00 (m, 2H), 1.71-1.69 (m, 4H), 1.30 (s, 6H);

^{13}C NMR (151 MHz, $CDCl_3$): δ 143.7, 139.5, 128.8, 125.9, 72.3, 63.8, 63.7, 51.2, 28.4, 23.8, 21.4, 20.7;

HRMS (ESI): Calc'd for $C_{12}H_{24}ON$ $[M-OTs]^+$: 198.1852, found: 198.1851.

Preparation of 4k

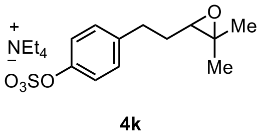

4k

Following the representative procedure C using alkene **3k** (38.6 mg, 0.100 mmol), epoxide **4k** (28.1 mg, 70%) was obtained as a white solid.

Melting Point: 50–55 °C (decomp.);

^{1}H NMR (600 MHz, $CDCl_3$): δ 7.27 (d, *J* = 8.3 Hz, 2H), 7.09 (d, *J* = 8.3 Hz, 2H), 3.27 (q, *J* = 7.3 Hz, 8H), 2.78-2.72 (m, 2H), 2.66-2.61 (m, 1H), 1.84-1.73 (m, 2H), 1.27 (t, *J* = 7.3 Hz, 12H), 1.25 (s, 3H), 1.14 (s, 3H);

^{13}C NMR (151 MHz, $CDCl_3$): δ 151.8, 136.8, 129.0, 121.3, 63.9, 58.7, 52.6, 32.2, 31.0, 24.9, 18.7, 7.6;

HRMS (ESI): Calc'd for $C_{12}H_{15}O_5S$ $[M-NEt_4]^-$: 271.0651, found: 271.0645.

Preparation of 4l

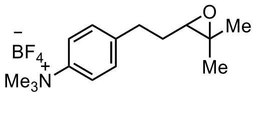

4l

Following the representative procedure C using alkene **3l** (30.5 mg, 0.100 mmol), epoxide **4l** (25.0 mg, 78%) was obtained as a white solid.

Melting Point: 67–72 °C (decomp.);

^{1}H NMR (500 MHz, $CDCl_3$): δ 7.67 (d, *J* = 8.6 Hz, 2H), 7.44 (d, *J* = 8.6 Hz, 2H), 3.69 (s, 9H), 2.92-2.86 (m, 1H), 2.83-2.77 (m, 1H), 2.73 (dd, *J* = 7.4, 5.2 Hz, 1H), 1.93-1.86 (m, 1H), 1.82-1.75 (m, 1H), 1.30 (s, 3H), 1.22 (s, 3H);

^{13}C NMR (151 MHz, $CDCl_3$): δ 145.1, 144.5, 130.6, 128.9, 125.9, 119.7, 77.3, 77.1, 76.9, 63.4, 58.7, 57.3, 32.1, 30.3, 24.8, 18.8;

^{19}F NMR (471 MHz, $CDCl_3$): δ −149.7;

HRMS (ESI): Calc'd for $C_{15}H_{24}ON$ $[M-BF_4]^+$: 234.1852, found: 234.1855.

4. Preparation of Epoxidation Catalysts

4.1. Representative procedure for the preparation of polymer catalyst

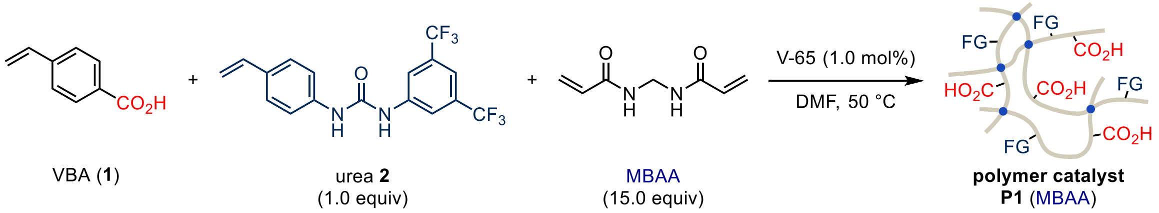

To a disposable glass tube containing 4-vinylbenzoic acid (**1**) (18.5 mg, 0.125 mmol, 1.00 equiv), 1,3-diarylurea **2**[72] (46.8 mg, 0.125 mmol, 1.00 equiv), *N,N'*-methylenebisacrylamide (MBAA) (289 mg, 1.88 mmol, 15.0 equiv) and 2,2'-azobis(2,4-dimethylvaleronitrile) (V-65) (9.9 mg, 0.040 mmol, 1.0 mol% relative to total polymerizable units), DMF (2.0 mL) was added, washing down the sides of the tube. The tube was flushed with argon, sealed with a rubber septum, and stirred at 50 °C on a thermo-mixer for 15 h. The resulting polymer block was ground into fine powder and transferred to a 50 mL conical tube. The powder was then centrifuged (7830 rpm, 20 min) with MeOH (15 mL) three times, and dried under vacuum at 70 °C for 12 h, affording **P1** as a white powder (345.7 mg).

Representative procedure for the determination of carboxylic acid content:

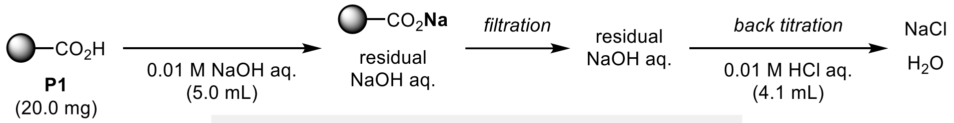

$$CO_2H \text{ content (mmol/g)} = \frac{0.01\times(5.0-4.1)\times1000}{20.0} = 0.45$$

The carboxylic acid content in polymer catalysts was determined according to a previously reported procedure [63]. **P1** (20.0 mg) was suspended in 0.01 M aqueous NaOH (5.0 mL) in a screw-capped test tube and stirred at room temperature for 30 min. The resulting mixture was filtered, and the solid residue was washed thoroughly with distilled water. The combined filtrate and washings were collected, to which five drops of 1% aqueous phenolphthalein solution were added as an indicator. The resulting solution was titrated with 0.01 M aqueous HCl until the solution became colorless. The volume of HCl consumed was used to calculate the carboxylic acid content of the polymer, which was found to be 0.45 mmol/g.

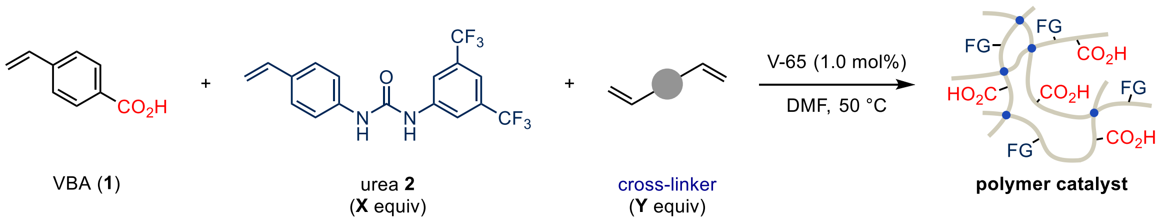

polymer catalyst	X (equiv)	cross-linker	Y (equiv)	CO_2H content (mmol/g)
**P1**	1.0	MBAA	15	0.45
**P2**	1.0	EGDA	15	0.41
**P3**	1.0	DVB	15	0.49
**P4**	1.0	EBAA	15	0.46
**P5**	1.0	DMEBAA	15	0.40
**P6**	1.0	BAP	15	0.40
**P7**	0	MBAA	15	0.46
**P8**	5.0	MBAA	15	0.32
**P9**	1.0	MBAA	5	0.62
**P10**	1.0	MBAA	10	0.52

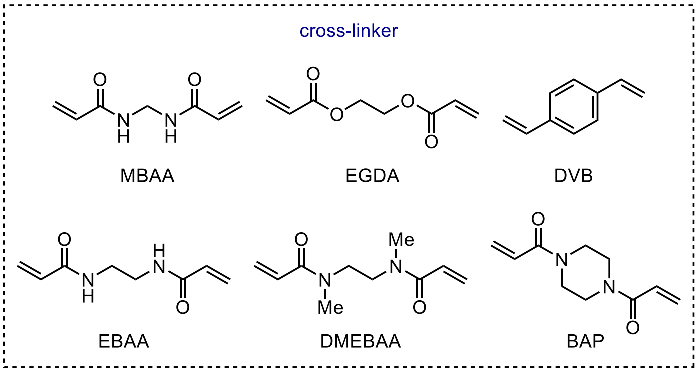

Figure S1. Characterizations of polymer catalysts

Preparation of P2

Prepared from 4-vinylbenzoic acid (**1**) (18.5 mg, 0.125 mmol, 1.00 equiv), 1,3-diarylurea **2** (46.8 mg, 0.125 mmol, 1.00 equiv) and ethylene glycol diacrylate (EGDA) (320 mg, 1.88 mmol, 15.0 equiv) following the representative procedure. **P2** was obtained as a white solid (276.6 mg).

Preparation of P3

Prepared from 4-vinylbenzoic acid (**1**) (18.5 mg, 0.125 mmol, 1.00 equiv), 1,3-diarylurea **2** (46.8 mg, 0.125 mmol, 1.00 equiv) and divinylbenzene (DVB) (245 mg, 1.88 mmol, 15.0 equiv) following the representative procedure. **P3** was obtained as a white solid (291.3 mg).

Preparation of P4

Prepared from 4-vinylbenzoic acid (**1**) (18.5 mg, 0.125 mmol, 1.00 equiv), 1,3-diarylurea **2** (46.8 mg, 0.125 mmol, 1.00 equiv) and *N,N'*-ethylenebisacrylamide (EBAA) (315 mg, 1.88 mmol, 15.0 equiv) following the representative procedure. **P4** was obtained as a white solid (353.0 mg).

Preparation of P5

Prepared from 4-vinylbenzoic acid (**1**) (18.5 mg, 0.125 mmol, 1.00 equiv), 1,3-diarylurea **2** (46.8 mg, 0.125 mmol, 1.00 equiv) and *N,N'*-dimethylethylenebisacrylamide (DMEBAA) (368 mg, 1.88 mmol, 15.0 equiv) following the representative procedure. **P5** was obtained as a white solid (399.2 mg).

Preparation of P6

Prepared from 4-vinylbenzoic acid (**1**) (18.5 mg, 0.125 mmol, 1.00 equiv), 1,3-diarylurea **2** (46.8 mg, 0.125 mmol, 1.00 equiv) and 1,4-bis(acryloyl)piperazine (BAP) (364 mg, 1.88 mmol, 15.0 equiv) following the

representative procedure. **P6** was obtained as a white solid (395.0 mg).

Preparation of P7

Prepared from 4-vinylbenzoic acid (**1**) (18.5 mg, 0.125 mmol, 1.00 equiv) and *N,N'*-methylenebisacrylamide (MBAA) (289 mg, 1.88 mmol, 15.0 equiv) following the representative procedure. **P7** was obtained as a white solid (302.5 mg).

Preparation of P8

Prepared from 4-vinylbenzoic acid (**1**) (18.5 mg, 0.125 mmol, 1.00 equiv), 1,3-diarylurea **2** (234 mg, 0.625 mmol, 5.00 equiv) and *N,N'*-methylenebisacrylamide (MBAA) (289 mg, 1.88 mmol, 15.0 equiv) following the representative procedure. **P8** was obtained as a white solid (516.5 mg).

Preparation of P9

Prepared from 4-vinylbenzoic acid (**1**) (18.5 mg, 0.125 mmol, 1.0 equiv), 1,3-diarylurea **2** (46.8 mg, 0.125 mmol, 1.00 equiv) and *N,N'*-methylenebisacrylamide (MBAA) (96.4 mg, 0.625 mmol, 5.00 equiv) following the representative procedure. **P9** was obtained as a white solid (134.3 mg).

Preparation of P10

Prepared from 4-vinylbenzoic acid (**1**) (18.5 mg, 0.125 mmol, 1.00 equiv), 1,3-diarylurea **2** (46.8 mg, 0.125 mmol, 1.00 equiv) and *N,N'*-methylenebisacrylamide (MBAA) (193 mg, 1.25 mmol, 10.0 equiv) following the representative procedure. **P10** was obtained as a white solid (244.2 mg).

4.2. Preparation of small-molecule catalysts

Representative procedure D

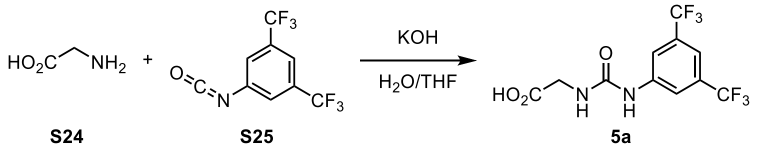

To a 4 mL scintillation vial containing a solution of **S24** (210 mg, 2.80 mmol, 1.40 equiv) and KOH (112 mg, 2.00 mmol, 1.00 equiv) in H_2O (0.6 mL) and THF (0.2 mL), **S25** (0.340 mL, 2.00 mmol, 1.00 equiv) was added. The resulting mixture was stirred at room temperature for 1 h. 1 N HCl aq. (3.0 mL) was then added to the reaction mixture, and the mixture was extracted with EtOAc (5 mL × 3). The combined organic layers were washed with brine, dried over $MgSO_4$, filtered and concentrated under reduced pressure to afford a white solid. Trituration with Et_2O provided **5a** as a white solid (536 mg, 81%).

Melting Point: 185–187 °C;

^{1}H NMR (600 MHz, CD_3OD): δ 7.93 (s, 2H), 7.41 (s, 1H), 3.87 (s, 2H);

^{13}C NMR (151 MHz, CD_3OD): δ 172.5, 156.0, 141.9, 131.8 (q, $^2J_{C–F}$ = 33.2 Hz), 123.5 (q, $^1J_{C–F}$ = 271.8 Hz), 117.7, 114.3, 41.0;

^{19}F NMR (471 MHz, CD_3OD): δ −64.6;
HRMS (ESI): Calc'd for $C_{11}H_7F_6N_2O_3$ $[M-H]^-$: 329.0361, found: 329.0370.

Preparation of 5b

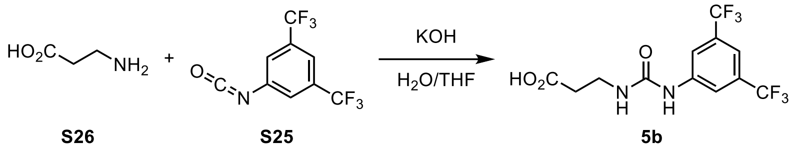

Following the representative procedure D using **S26** (249 mg, 2.80 mmol, 1.40 equiv), **5b** (534 mg, 78%) was obtained as a white solid.
Melting Point: 191–193 °C;
^{1}H NMR (600 MHz, CD_3OD): δ 7.97 (s, 2H), 7.45 (s, 1H), 3.45 (t, J = 6.2 Hz, 2H), 2.53 (t, J = 6.2 Hz, 2H);
^{13}C NMR (151 MHz, CD_3OD): δ 174.4, 155.9, 142.0, 131.8 (q, $^2J_{C-F}$ = 33.2 Hz), 123.5 (q, $^1J_{C-F}$ = 271.8 Hz), 117.6, 114.1, 35.3, 34.0;
^{19}F NMR (471 MHz, CD_3OD): δ −64.6;
HRMS (ESI): Calc'd for $C_{12}H_9F_6N_2O_3$ $[M-H]^-$: 343.0517, found: 343.0527.

Preparation of 5c

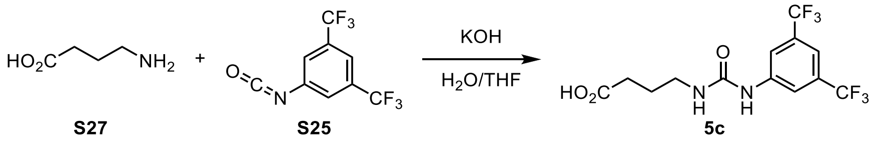

Following the representative procedure D using **S27** (289 mg, 2.80 mmol, 1.40 equiv), **5c** (534 mg, 75%) was obtained as a white solid.
Melting Point: 159–161 °C;
^{1}H NMR (600 MHz, CD_3OD): δ 7.91 (s, 2H), 7.37 (s, 1H), 3.16 (t, J = 6.9 Hz, 2H), 2.28 (t, J = 7.6 Hz, 2H), 1.74 (tt, J = 6.9, 7.6 Hz, 2H);
^{13}C NMR (151 MHz, CD_3OD): δ 175.7, 156.1, 142.1, 131.8 (q, $^2J_{C-F}$ = 33.2 Hz), 123.5 (q, $^1J_{C-F}$ = 271.8 Hz), 117.7, 114.1, 38.9, 30.8, 25.1;
^{19}F NMR (471 MHz, CD_3OD): δ −64.6;
HRMS (ESI): Calc'd for $C_{13}H_{11}F_6N_2O_3$ $[M-H]^-$: 357.0674, found: 357.0684.

Preparation of 5d

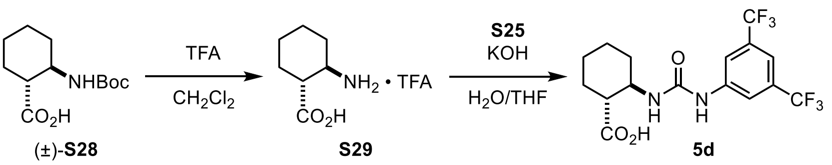

To a 30 mL round-bottom flask containing a solution of (±)-**S28** (730 mg, 3.00 mmol, 1.50 equiv) in CH_2Cl_2 (3.0 mL), trifluoroacetic acid (2.30 mL, 30.0 mmol, 15.0 equiv) was added. The resulting mixture was stirred

at room temperature for 14 h, and then concentrated under reduced pressure. The crude residue was triturated with Et_2O to provide **S29** was a white solid that was used in the next step without further purification. To a 10 mL round-bottom flask containing a solution of **S29** and KOH (224 mg, 4.00 mmol, 2.00 equiv) in H_2O (0.6 mL) and THF (0.2 mL), **S25** (0.340 mL, 2.00 mmol, 1.00 equiv) was added. The resulting mixture was stirred at room temperature for 1 h. 1 N HCl aq. (3.0 mL) was then added to the reaction mixture, and the mixture was extracted with EtOAc (5 mL × 3). The combined organic layers were washed with brine, dried over $MgSO_4$, filtered and concentrated under reduced pressure to afford a white solid. Trituration with Et_2O provided **5d** as a white solid (503 mg, 63%).

Melting Point: 204–206 °C;

^{1}H NMR (600 MHz, CD_3OD): δ 7.95 (s, 2H), 7.44 (s, 1H), 3.82 (td, J = 11.0, 3.4 Hz, 1H), 2.29 (td, J = 11.0, 3.9 Hz, 1H), 2.03-1.95 (m, 2H), 1.77-1.72 (m, 2H), 1.60-1.53 (m, 1H), 1.44-1.37 (m, 1H), 1.30-1.23 (m, 2H);

^{13}C NMR (151 MHz, CD_3OD): δ 176.7, 155.2, 142.1, 131.8 (q, $^2J_{C–F}$ = 33.2 Hz), 123.5 (q, $^1J_{C–F}$ = 271.8 Hz), 117.7, 114.1, 50.3, 49.5, 32.6, 29.0, 24.6, 24.3;

^{19}F NMR (471 MHz, CD_3OD): δ −64.5;

HRMS (ESI): Calc'd for $C_{16}H_{15}F_6N_2O_3$ $[M-H]^-$: 397.0987, found: 397.0994.

Preparation of 5e

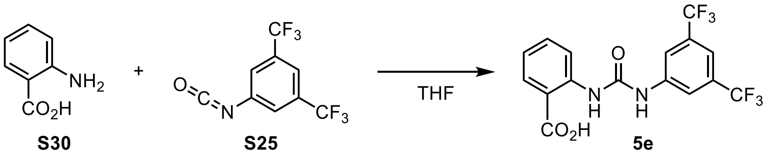

To a 50 mL round-bottom flask containing a solution of **S30** (274 mg, 2.00 mmol, 1.00 equiv) in THF (10 mL), **S25** (0.377 mL, 2.20 mmol, 1.10 equiv) was added. The resulting mixture was stirred at room temperature for 12 h, and then concentrated under reduced pressure. The resulting solid was washed with hexanes (50 mL×5) to afford **5e** as a white solid (750 mg, 96%).

Melting Point: 192–195 °C;

^{1}H NMR (500 MHz, CD_3OD): δ 8.41 (dd, J = 8.5, 1.0 Hz, 1H), 8.13 (s, 2H), 8.05 (dd, J = 8.0, 1.7 Hz, 1H), 7.55-7.51 (m, 2H), 7.06 (ddd, J = 8.0, 7.5, 1.0 Hz, 1H);

^{13}C NMR (126 MHz, CD_3OD): δ 170.0, 152.9, 142.0, 141.9, 133.7, 131.8 (q, $^2J_{C–F}$ = 33.2 Hz), 131.1, 123.5 (q, $^1J_{C–F}$ = 271.7 Hz), 121.3, 119.7, 118.2, 115.5, 114.7;

^{19}F NMR (471 MHz, CD_3OD): δ −64.5;

HRMS (ESI): Calc'd for $C_{16}H_9F_6N_2O_3$ $[M-H]^-$: 391.0523, found: 391.0528.

5. Competitive Epoxidation Reactions

Representative procedure E

A 4 mL scintillation vial containing a stir bar was charged with **3a** (19.5 mg, 0.0500 mmol, 1.00 equiv), **3b** (12.5 mg, 0.0500 mmol, 1.00 equiv) and benzoic acid (1.2 mg, 0.010 mmol, 20 mol%). A solution of HOBt·H_2O and DMAP in CH_2Cl_2 (0.02 M, 250 μL, 0.0050 mmol, 10 mol% each), CH_2Cl_2 (750 μL), DIC (7.7 μL, 0.050 mmol, 1.0 equiv) and H_2O_2 (35% aq., 9.7 μL, 0.10 mmol, 2.0 equiv) were added in this order, and the resulting mixture was stirred at room temperature. After 12 h, the reaction mixture was transferred to a 15 mL conical tube, and treated with sat. $Na_2S_2O_3$ aq. (50 μL). The reaction mixture was then centrifuged (7830 rpm, 10 min) with MeCN (10 mL) three times, and the combined supernatants were concentrated under reduced pressure. ^1H NMR analysis using triphenylmethane as an internal standard revealed the formation of epoxides **4a** and **4b** in a combined yield of 88% (**4a**: 36%, **4b**: 52%), with a product ratio of **4a**/**4b** = 0.7, as confirmed by the spectra shown in **Figure S2**.

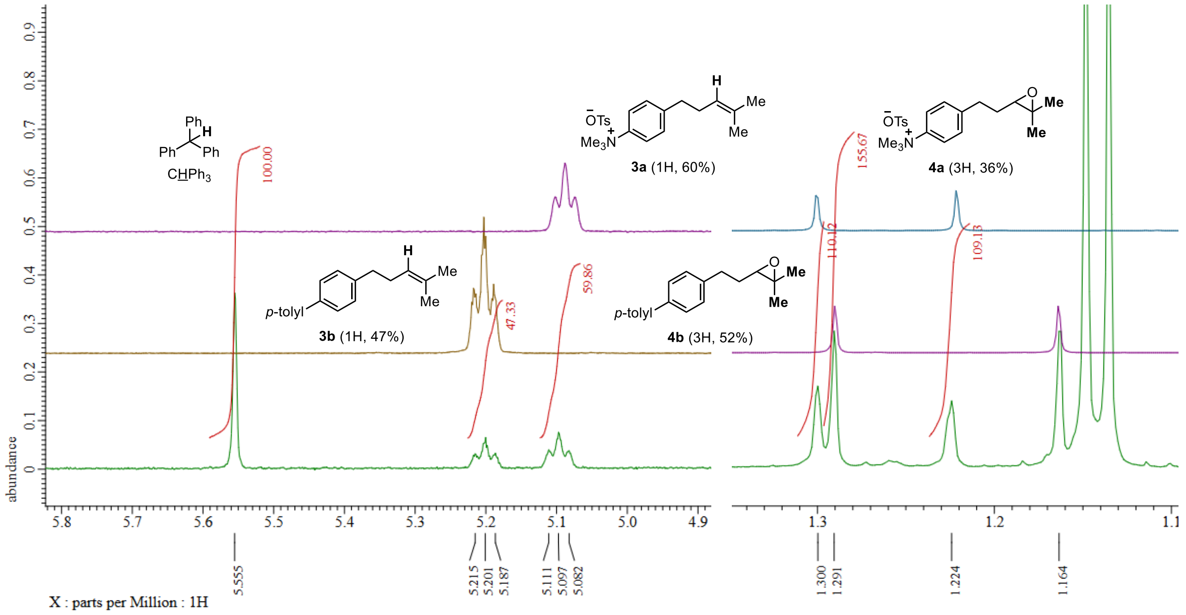

Figure S2. Analysis of the product ratio in competitive reaction by ^1H NMR.

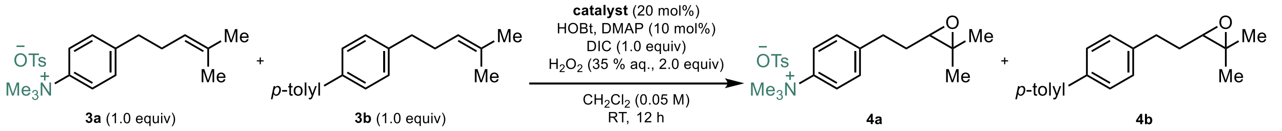

Entry	Catalyst	NMR Yield (%) 4a	4b	4a+4b	Ratio 4a/4b
1	PhCO$_2$H	36	52	88	0.7
2	**P1**	66	9	75	7.3
3	**P2**	69	20	89	3.5
4	**P3**	53	25	78	2.1
5	**P4**	67	9	76	7.4
6	**P5**	61	9	70	6.8
7	**P6**	58	21	79	2.8
8	**P7**	56	9	65	6.2
9	**P8**	59	12	71	4.9
10	**P9**	64	12	76	5.3
11	**P10**	62	10	72	6.2

Entry	Catalyst	additive	NMR Yield (%) 4a	4b	4a+4b	Ratio 4a/4b
12	**5a**	-	46	19	65	2.4
13	**5b**	-	61	37	98	1.7
14	**5c**	-	63	30	93	2.1
15	**5d**	-	51	26	77	2.0
16	**5e**	-	43	28	71	1.5
17	**P1**	**7** (2.0 equiv)	50	11	61	4.6
18	**P1**	DMF (0.5 mL)	18	6	24	3.0

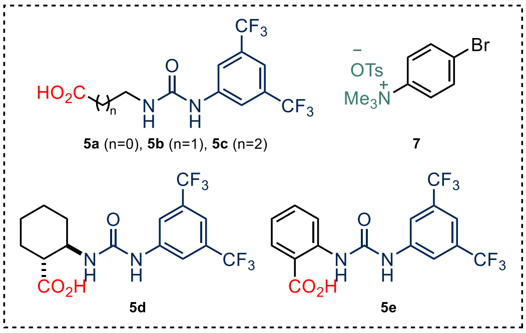

Figure S3. Evaluation of epoxidation catalysts

Entry 2

Following the representative procedure E, epoxidation was conducted with **P1** (22 mg, 0.010 mmol, 20 mol%). ^{1}H NMR analysis indicated the formation of epoxides **4a** and **4b** in a combined yield of 75% (**4a**: 66%, **4b**: 9%) with a product ratio of **4a**/**4b** = 7.3.

Entry 3

Following the representative procedure E, epoxidation was conducted with **P2** (25 mg, 0.010 mmol, 20 mol%). ^{1}H NMR analysis indicated the formation of epoxides **4a** and **4b** in a combined yield of 89% (**4a**: 69%, **4b**: 20%) with a product ratio of **4a**/**4b** = 3.5.

Entry 4

Following the representative procedure E, epoxidation was conducted with **P3** (20 mg, 0.010 mmol, 20 mol%). ^{1}H NMR analysis indicated the formation of epoxides **4a** and **4b** in a combined yield of 78% (**4a**: 53%, **4b**: 25%) with a product ratio of **4a**/**4b** = 2.1.

Entry 5

Following the representative procedure E, epoxidation was conducted with **P4** (22 mg, 0.010 mmol, 20 mol%). ^{1}H NMR analysis indicated the formation of epoxides **4a** and **4b** in a combined yield of 76% (**4a**: 67%, **4b**: 9%) with a product ratio of **4a**/**4b** = 7.4.

Entry 6

Following the representative procedure E, epoxidation was conducted with **P5** (25 mg, 0.010 mmol, 20 mol%). ^{1}H NMR analysis indicated the formation of epoxides **4a** and **4b** in a combined yield of 70% (**4a**: 61%, **4b**: 9%) with a product ratio of **4a**/**4b** = 6.8.

Entry 7

Following the representative procedure E, epoxidation was conducted with **P6** (25 mg, 0.010 mmol, 20 mol%). ^{1}H NMR analysis indicated the formation of epoxides **4a** and **4b** in a combined yield of 79% (**4a**: 58%, **4b**: 21%) with a product ratio of **4a**/**4b** = 2.8.

Entry 8

Following the representative procedure E, epoxidation was conducted with **P7** (24 mg, 0.010 mmol, 20 mol%). ^{1}H NMR analysis indicated the formation of epoxides **4a** and **4b** in a combined yield of 65% (**4a**: 56%, **4b**: 9%) with a product ratio of **4a**/**4b** = 6.2.

Entry 9

Following the representative procedure E, epoxidation was conducted with **P8** (32 mg, 0.010 mmol, 20 mol%). ^{1}H NMR analysis indicated the formation of epoxides **4a** and **4b** in a combined yield of 71% (**4a**: 59%, **4b**:

12%) with a product ratio of **4a**/**4b** = 4.9.

Entry 10

Following the representative procedure E, epoxidation was conducted with **P9** (16 mg, 0.010 mmol, 20 mol%). ^{1}H NMR analysis indicated the formation of epoxides **4a** and **4b** in a combined yield of 76% (**4a**: 64%, **4b**: 12%) with a product ratio of **4a**/**4b** = 5.3.

Entry 11

Following the representative procedure E, epoxidation was conducted with **P10** (19 mg, 0.010 mmol, 20 mol%). ^{1}H NMR analysis indicated the formation of epoxides **4a** and **4b** in a combined yield of 72% (**4a**: 62%, **4b**: 10%) with a product ratio of **4a**/**4b** = 6.2.

Entry 12

Following the representative procedure E, epoxidation was conducted with **5a** (3.3 mg, 0.010 mmol, 20 mol%). ^{1}H NMR analysis indicated the formation of epoxides **4a** and **4b** in a combined yield of 65% (**4a**: 46%, **4b**: 19%) with a product ratio of **4a**/**4b** = 2.4.

Entry 13

Following the representative procedure E, epoxidation was conducted with **5b** (3.4 mg, 0.010 mmol, 20 mol%). ^{1}H NMR analysis indicated the formation of epoxides **4a** and **4b** in a combined yield of 98% (**4a**: 61%, **4b**: 37%) with a product ratio of **4a**/**4b** = 1.7.

Entry 14

Following the representative procedure E, epoxidation was conducted with **5c** (3.6 mg, 0.010 mmol, 20 mol%). ^{1}H NMR analysis indicated the formation of epoxides **4a** and **4b** in a combined yield of 93% (**4a**: 63%, **4b**: 30%) with a product ratio of **4a**/**4b** = 2.1.

Entry 15

Following the representative procedure E, epoxidation was conducted with **5d** (4.0 mg, 0.010 mmol, 20 mol%). ^{1}H NMR analysis indicated the formation of epoxides **4a** and **4b** in a combined yield of 77% (**4a**: 51%, **4b**: 26%) with a product ratio of **4a**/**4b** = 2.0.

Entry 16

Following the representative procedure E, epoxidation was conducted with **5e** (3.9 mg, 0.010 mmol, 20 mol%). ^{1}H NMR analysis indicated the formation of epoxides **4a** and **4b** in a combined yield of 71% (**4a**: 43%, **4b**: 28%) with a product ratio of **4a**/**4b** = 1.5.

Entry 17

Following the representative procedure E, epoxidation was conducted with **P1** (22 mg, 0.010 mmol, 20 mol%) and ammonium salt **7** (38.5 mg, 0.100 mmol, 2.00 equiv). ^{1}H NMR analysis indicated the formation of epoxides **4a** and **4b** in a combined yield of 61% (**4a**: 50%, **4b**: 11%) with a product ratio of **4a**/**4b** = 4.6.

Entry 18

Following the representative procedure E, epoxidation was conducted with **P1** (22 mg, 0.010 mmol, 20 mol%) and DMF (0.5 mL). ^{1}H NMR analysis indicated the formation of epoxides **4a** and **4b** in a combined yield of 24% (**4a**: 18%, **4b**: 6%) with a product ratio of **4a**/**4b** = 3.0.

5.1. Reproducibility test

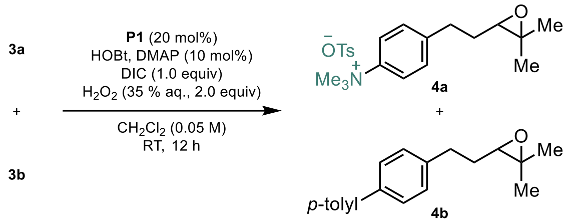

Batch	NMR Yield (%) 3a	3b	4a	4b	4a+4b	Ratio 4a/4b
1	33	90	66	9	75	7.3
2	37	88	63	9	72	7.0
3	37	89	62	8	70	7.8

Competitive epoxidation of **3a** and **3b** with three independently prepared batches of catalyst **P1** yielded identical outcomes, confirming the full reproducibility of the catalysis.

5.2. Substrate generality

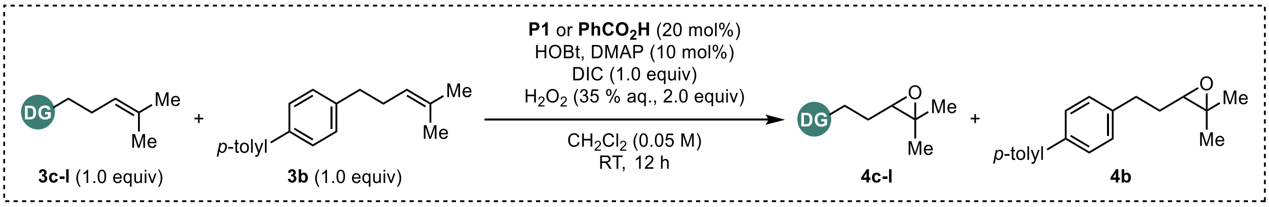

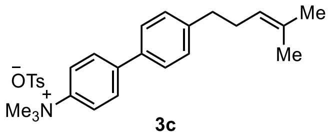

3c

Following the representative procedure E, epoxidation was conducted with **3c** (20.3 mg, 0.0500 mmol, 1.00 equiv), **3b** (12.5 mg, 0.0500 mmol, 1.00 equiv) and **P1** (22 mg, 0.010 mmol, 20 mol%). ^{1}H NMR analysis indicated the formation of epoxides **4c** and **4b** in a combined yield of 76% (**4c**: 67%, **4b**: 9%) with a product ratio of **4c**/**4b** = 7.4.

Following the representative procedure E, epoxidation was conducted with benzoic acid (1.2 mg, 0.010 mmol, 20 mol%). ^{1}H NMR analysis indicated the formation of epoxides **4c** and **4b** in a combined yield of 82% (**4c**: 34%, **4b**: 48%) with a product ratio of **4c**/**4b** = 0.7.

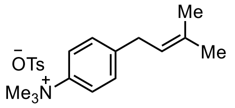

3d

Following the representative procedure E, epoxidation was conducted with **3d** (18.8 mg, 0.0500 mmol, 1.00 equiv), **3b** (12.5 mg, 0.0500 mmol, 1.00 equiv) and **P1** (22 mg, 0.010 mmol, 20 mol%). ^{1}H NMR analysis indicated the formation of epoxides **4d** and **4b** in a combined yield of 63% (**4d**: 48%, **4b**: 15%) with a product ratio of **4d**/**4b** = 3.2.

Following the representative procedure E, epoxidation was conducted with benzoic acid (1.2 mg, 0.010 mmol, 20 mol%). ^{1}H NMR analysis indicated the formation of epoxides **4d** and **4b** in a combined yield of 78% (**4d**: 19%, **4b**: 59%) with a product ratio of **4d**/**4b** = 0.3.

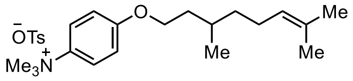

3e

Following the representative procedure E, epoxidation was conducted with **3e** (23.1 mg, 0.0500 mmol, 1.00 equiv), **3b** (12.5 mg, 0.0500 mmol, 1.00 equiv) and **P1** (22 mg, 0.010 mmol, 20 mol%). ^{1}H NMR analysis indicated the formation of epoxides **4e** and **4b** in a combined yield of 95% (**4e**: 85%, **4b**: 10%) with a product ratio of **4e**/**4b** = 8.5.

Following the representative procedure E, epoxidation was conducted with benzoic acid (1.2 mg, 0.010 mmol, 20 mol%). ^{1}H NMR analysis indicated the formation of epoxides **4e** and **4b** in a combined yield of 100% (**4e**: 50%, **4b**: 50%) with a product ratio of **4e**/**4b** = 1.0.

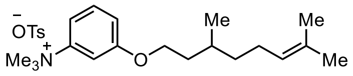

3f

Following the representative procedure E, epoxidation was conducted with **3f** (23.1 mg, 0.0500 mmol, 1.00 equiv), **3b** (12.5 mg, 0.0500 mmol, 1.00 equiv) and **P1** (22 mg, 0.010 mmol, 20 mol%). ^{1}H NMR analysis indicated the formation of epoxides **4f** and **4b** in a combined yield of 71% (**4f**: 64%, **4b**: 7%) with a product ratio of **4f**/**4b** = 9.1.

Following the representative procedure E, epoxidation was conducted with benzoic acid (1.2 mg, 0.010 mmol, 20 mol%). ^{1}H NMR analysis indicated the formation of epoxides **4f** and **4b** in a combined yield of 94% (**4f**: 44%, **4b**: 50%) with a product ratio of **4f**/**4b** = 0.9.

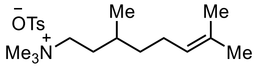

3g

Following the representative procedure E, epoxidation was conducted with **3g** (18.5 mg, 0.0500 mmol, 1.00 equiv), **3b** (12.5 mg, 0.0500 mmol, 1.00 equiv) and **P1** (22 mg, 0.010 mmol, 20 mol%). ^{1}H NMR analysis indicated the formation of epoxides **4g** and **4b** in a combined yield of 57% (**4g**: 51%, **4b**: 6%) with a product

ratio of **4g**/**4b** = 8.5.

Following the representative procedure E, epoxidation was conducted with benzoic acid (1.2 mg, 0.010 mmol, 20 mol%). ^{1}H NMR analysis indicated the formation of epoxides **4g** and **4b** in a combined yield of 78% (**4g**: 42%, **4b**: 36%) with a product ratio of **4g**/**4b** = 1.2.

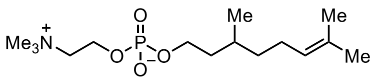

3h

Following the representative procedure E, epoxidation was conducted with **3h** (16.1 mg, 0.0500 mmol, 1.00 equiv), **3b** (12.5 mg, 0.0500 mmol, 1.00 equiv) and **P1** (22 mg, 0.010 mmol, 20 mol%). ^{1}H NMR analysis indicated the formation of epoxides **4h** and **4b** in a combined yield of 46% (**4h**: 40%, **4b**: 6%) with a product ratio of **4h**/**4b** = 6.7.

Following the representative procedure E, epoxidation was conducted with benzoic acid (1.2 mg, 0.010 mmol, 20 mol%). ^{1}H NMR analysis indicated the formation of epoxides **4h** and **4b** in a combined yield of 86% (**4h**: 39%, **4b**: 47%) with a product ratio of **4h**/**4b** = 0.8.

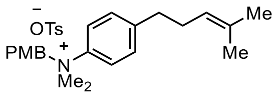

3i

Following the representative procedure E, epoxidation was conducted with **3i** (24.8 mg, 0.0500 mmol, 1.00 equiv), **3b** (12.5 mg, 0.0500 mmol, 1.00 equiv) and **P1** (22 mg, 0.010 mmol, 20 mol%). ^{1}H NMR analysis indicated the formation of epoxides **4i** and **4b** in a combined yield of 71% (**4i**: 61%, **4b**: 10%) with a product ratio of **4i**/**4b** = 6.1.

Following the representative procedure E, epoxidation was conducted with benzoic acid (1.2 mg, 0.010 mmol, 20 mol%). ^{1}H NMR analysis indicated the formation of epoxides **4i** and **4b** in a combined yield of 86% (**4i**: 35%, **4b**: 51%) with a product ratio of **4i**/**4b** = 0.7.

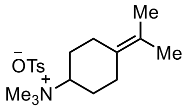

3j

Following the representative procedure E, epoxidation was conducted with **3j** (17.8 mg, 0.0500 mmol, 1.00 equiv), **3b** (12.5 mg, 0.0500 mmol, 1.00 equiv) and **P1** (22 mg, 0.010 mmol, 20 mol%). ^{1}H NMR analysis indicated the formation of epoxides **4j** and **4b** in a combined yield of 68% (**4j**: 68%, **4b**: <5%) with a product ratio of **4j**/**4b** = >20.

Following the representative procedure E, epoxidation was conducted with benzoic acid (1.2 mg, 0.010 mmol, 20 mol%). ^{1}H NMR analysis indicated the formation of epoxides **4j** and **4b** in a combined yield of 97% (**4j**: 68%, **4b**: 29%) with a product ratio of **4j**/**4b** = 2.3.

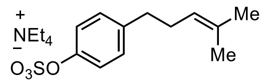

3k

Following the representative procedure E, epoxidation was conducted with **3k** (19.3 mg, 0.0500 mmol, 1.00 equiv), **3b** (12.5 mg, 0.0500 mmol, 1.00 equiv) and **P1** (22 mg, 0.010 mmol, 20 mol%). ^{1}H NMR analysis indicated the formation of epoxides **4k** and **4b** in a combined yield of 81% (**4k**: 71%, **4b**: 10%) with a product ratio of **4k**/**4b** = 7.8.

Following the representative procedure E, epoxidation was conducted with benzoic acid (1.2 mg, 0.010 mmol, 20 mol%). ^{1}H NMR analysis indicated the formation of epoxides **4k** and **4b** in a combined yield of 75% (**4k**: 40%, **4b**: 35%) with a product ratio of **4k**/**4b** = 1.1.

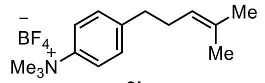

3l

Following the representative procedure E, epoxidation was conducted with **3l** (15.3 mg, 0.0500 mmol, 1.00 equiv), **3b** (12.5 mg, 0.0500 mmol, 1.00 equiv) and **P1** (22 mg, 0.010 mmol, 20 mol%). ^{1}H NMR analysis indicated the formation of epoxides **4l** and **4b** in a combined yield of 74% (**4l**: 63%, **4b**: 11%) with a product ratio of **4l**/**4b** = 5.7.

Following the representative procedure E, epoxidation was conducted with benzoic acid (1.2 mg, 0.010 mmol, 20 mol%). ^{1}H NMR analysis indicated the formation of epoxides **4l** and **4b** in a combined yield of 83% (**4l**: 35%, **4b**: 48%) with a product ratio of **4l**/**4b** = 0.7.

5.3. Chemoselective epoxidation of ionic alkene 3a in the presence of biomolecules.

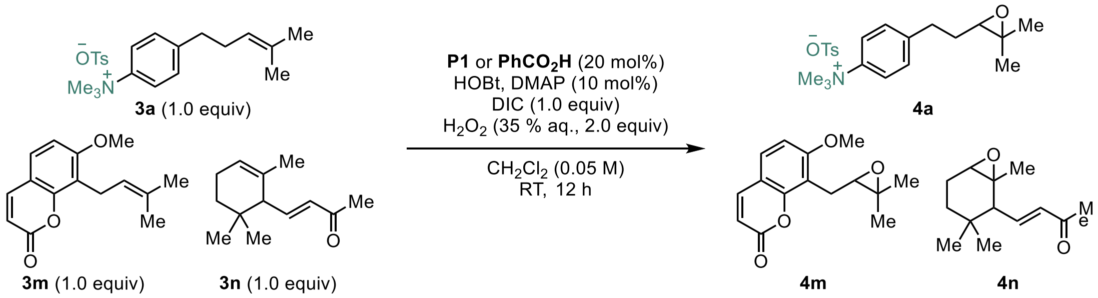

Entry	Catalyst	3a	3m	3n	4a	4m	4n
		NMR Yield (%)					
1	$PhCO_2H$	48	53	85	44	41	9
2	**P1**	25	84	89	71	13	6

Following the representative procedure E, epoxidation was conducted with **3a** (19.5 mg, 0.0500 mmol, 1.00 equiv), **3m** (12.2 mg, 0.0500 mmol, 1.00 equiv), **3n** (9.6 mg, 0.0500 mmol, 1.00 equiv) and benzoic acid (1.2 mg, 0.010 mmol, 20 mol%). ^{1}H NMR analysis indicated the formation of epoxides **4a**, **4m** [73] and **4n** [74] in a combined yield of 94% (**4a**: 44%, **4m**: 41%, **4n**: 9%).

Following the representative procedure E, epoxidation was conducted with **3a** (19.5 mg, 0.0500 mmol, 1.00 equiv), **3m** (12.2 mg, 0.0500 mmol, 1.00 equiv), **3n** (9.6 mg, 0.0500 mmol, 1.00 equiv) and **P1** (22 mg, 0.010

mmol, 20 mol%). ^{1}H NMR analysis indicated the formation of epoxides **4a**, **4m** and **4n** in a combined yield of 90% (**4a**: 71%, **4m**: 13%, **4n**: 6%).

5.4. Catalyst reusability

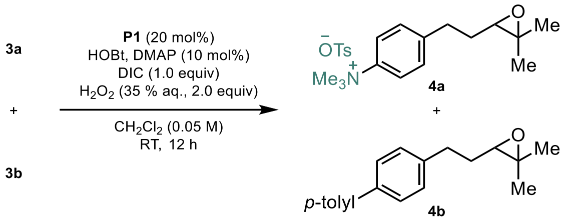

Cycle	NMR Yield (%)					Ratio
	**3a**	**3b**	**4a**	**4b**	**4a+4b**	**4a/4b**
1	30	61	93	7	68	8.7
2	41	58	92	7	65	8.3
3	41	54	92	8	62	6.8
4	42	58	88	7	65	8.3
5	53	47	94	6	53	7.8

Following the representative procedure E, epoxidation was conducted with **P1** (22 mg, 0.010 mmol, 20 mol%). After 12 h, the insoluble polymer, isolated by centrifugation with MeCN (3 × 10 mL), was sequentially centrifuged with MeOH (10 mL) three times and Et_2O (10 mL) two times, and then dried under reduced pressure overnight. The dried polymer was directly reused for the next reaction cycle.

6. ¹H NMR Titration Experiments

6.1. Interaction between trimethylammonium 3a and bis-amide 6

An NMR tube was charged with a solution of **3a** and **6** [75] in $CDCl_3$ (0.033 M each), and the molar ratios of the two components were varied while keeping the total amount constant at 0.020 mmol in 600 μL. Samples were mixed and maintained at 25 °C prior to ¹H NMR analysis.

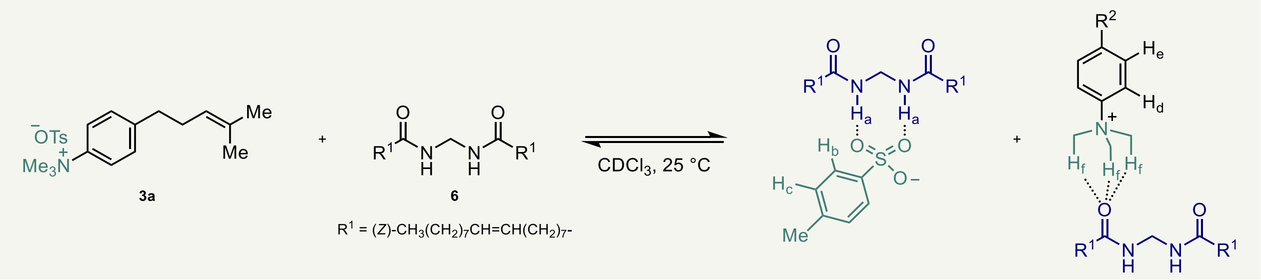

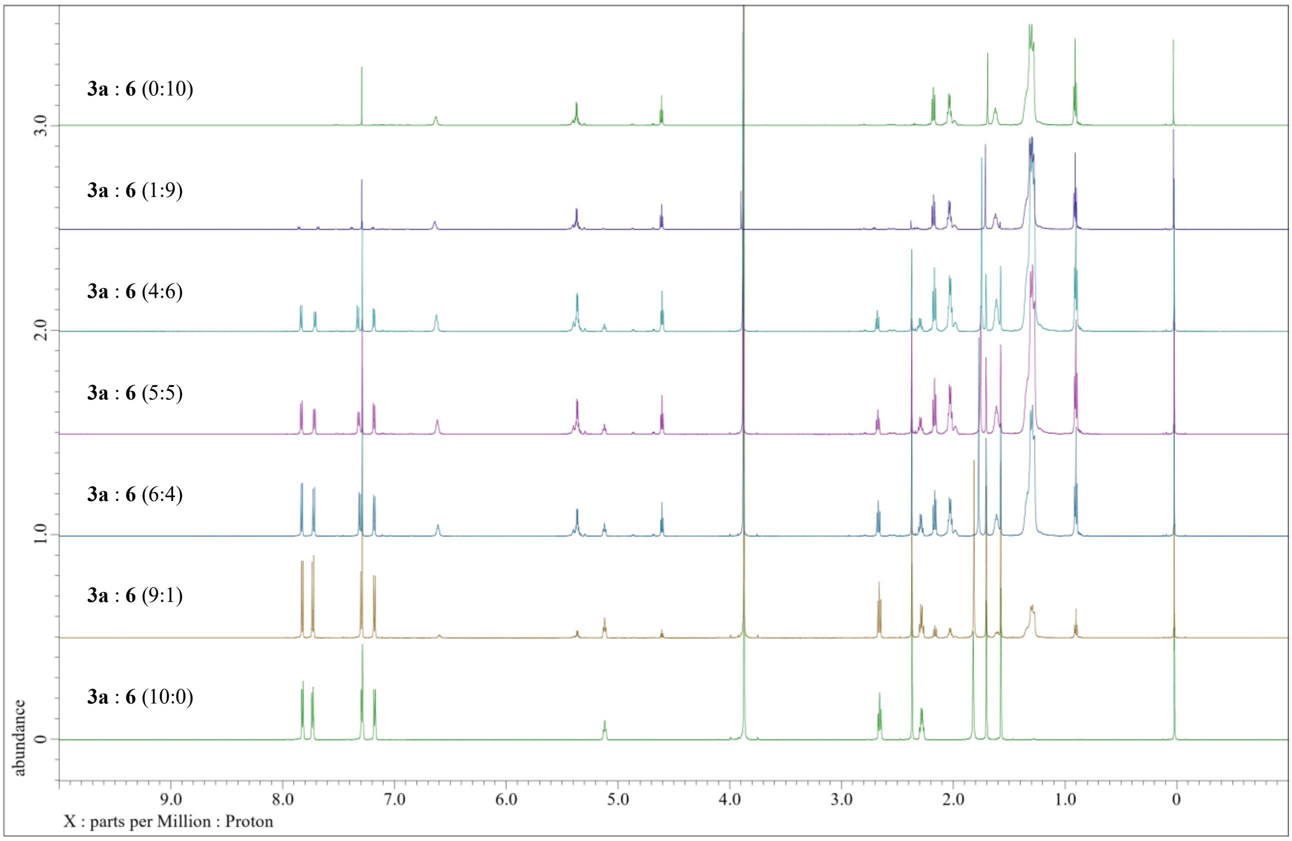

6.2. Interaction between trimethylammonium 3a and urea 8

An NMR tube was charged with a solution of **3a** and **8** [76] in $CDCl_3$ (0.033 M each), and the molar ratios of the two components were varied while keeping the total amount constant at 0.020 mmol in 600 µL. Samples were mixed and maintained at 25 °C prior to ^1H NMR analysis.

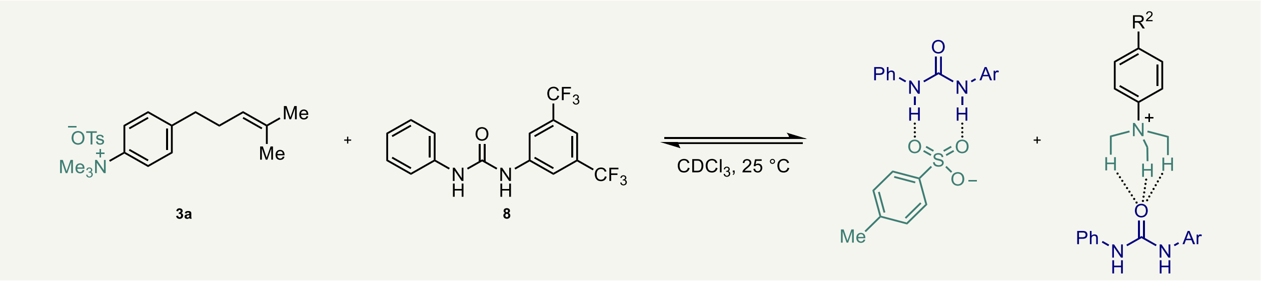

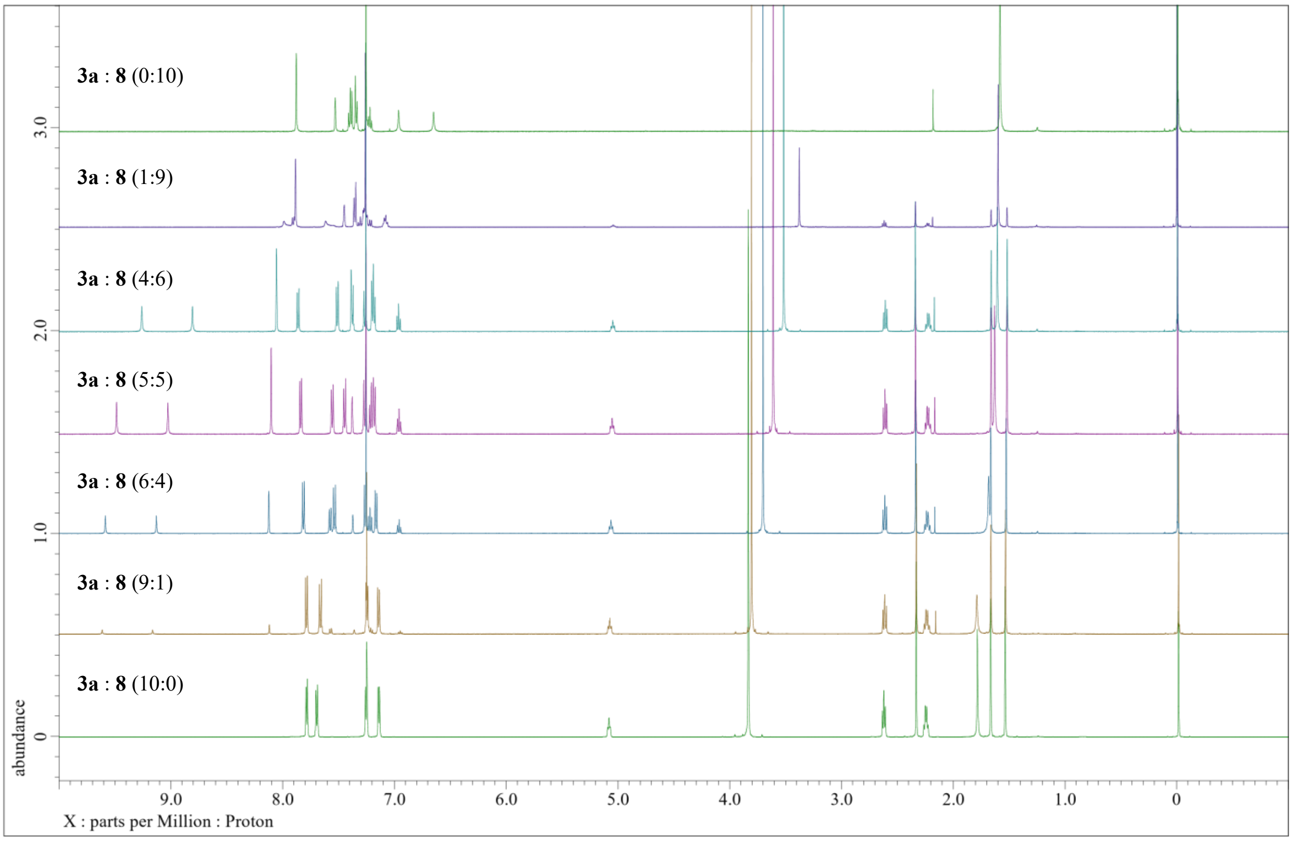

7. References

63. H. Chen, Y. Wang, Q. Wang, J. Li, S. Yang, and Z. Zhu, “Bifunctional Organic Polymeric Catalysts With a Tunable Acid-Base Distance and Framework Flexibility,” *Scientific Reports* 4 (2014): 6475.

67. W. Li and T. Werner, “$B(C_6F_5)_3$-Catalyzed Michael Reactions: Aromatic C–H as Nucleophiles,” *Organic Letters* 19 (2017): 2568–2571.

68. X. Lv, P. Gao, X. Zhao, and Z. Jiang, “Metal-Free Construction of Multisubstituted Indolizines via Intramolecular Amination of Allylic Alcohols,” *Journal of Organic Chemistry* 88 (2023): 9459–9468.

69. M. D. Pungente, H.-R. Sliwka, C. L. Øpstad, et al., “Novel Cationic Polyene Glycol Phospholipids as DNA Transfer Reagents—Lack of a Structure–activity Relationship due to Uncontrolled Self-assembling Processes,” *Chemistry and Physics of Lipids* 183 (2014): 117–136.

70. Y. Li, H. Xu, J. He, et al., “Domino Aryne Annulation via a Nucleophilic–Ene Process,” *Journal of the American Chemistry Society* 140 (2018): 3555–3559.

71. F. T. Schevenels, M. Shen, and S. A. Snyder, “Alkyldisulfanium Salts: Isolable, Electrophilic Sulfur Reagents Competent for Polyene Cyclizations,” *Organic Letters* 19 (2017): 2–5.

72. A. J. Hall, P. Manesiotis, M. Emgenbroich, M. Quaglia, E. De Lorenzi, and B. Sellergren, “Urea Host Monomers for Stoichiometric Molecular Imprinting of Oxyanions,” *Journal of Organic Chemistry* 70 (2005): 17321736.

73. Z. Ren, M. Lv, Y. Yang, et al., “Structural Optimization of Natural Plant Products: Construction, Pesticidal Activities, and Toxicology Study of New 2-Isopropanol-4-methoxy-7-alkyl/Aryloxycarbonyl-(E)-vinyl-2,3-dihydrobenzofurans,” *Journal of Agricultural and Food Chemistry* 73 (2025): 1804–1812.

74. G. Majetich, J. Shimkus, and Y. Li, “Epoxidation of Olefins by β-bromoalkoxydimethylsulfonium Ylides,” *Tetrahedron Letters* 51 (2010): 6830–6834.

75. B. F. Cravatt, R. A. Lerner, and D. L. Boger, “Structure Determination of an Endogenous Sleep-Inducing Lipid, cis-9-Octadecenamide (Oleamide): A Synthetic Approach to the Chemical Analysis of Trace Quantities of a Natural Product,” *Journal of the American Chemical Society* 118 (1996): 580–590.

76. M. Blain, H. Yau, L. Jean-Gérard, et al., “Urea- and Thiourea-Catalyzed Aminolysis of Carbonates,” *Chemsuschem* 9 (2016): 2269–2272.

8. NMR Spectra

500 MHz, $CDCl_3$

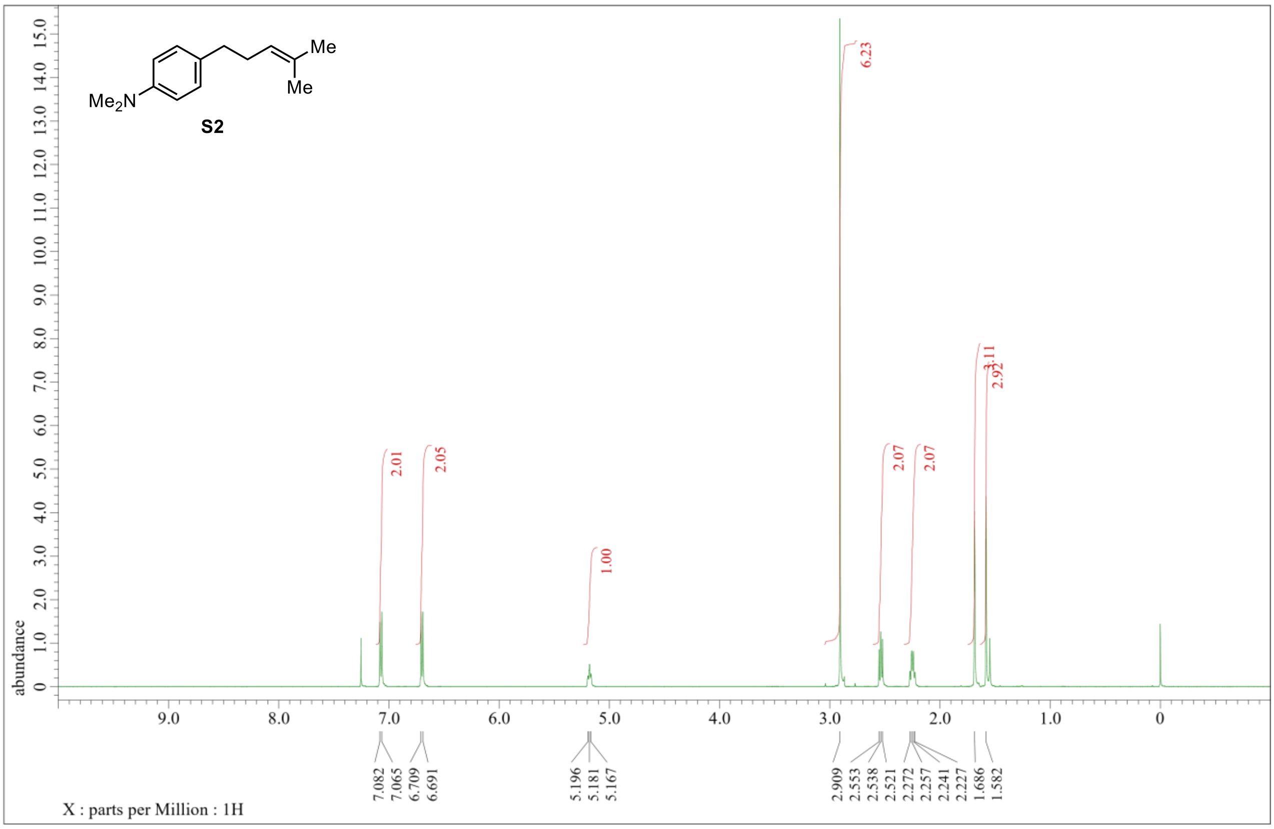

151 MHz, $CDCl_3$

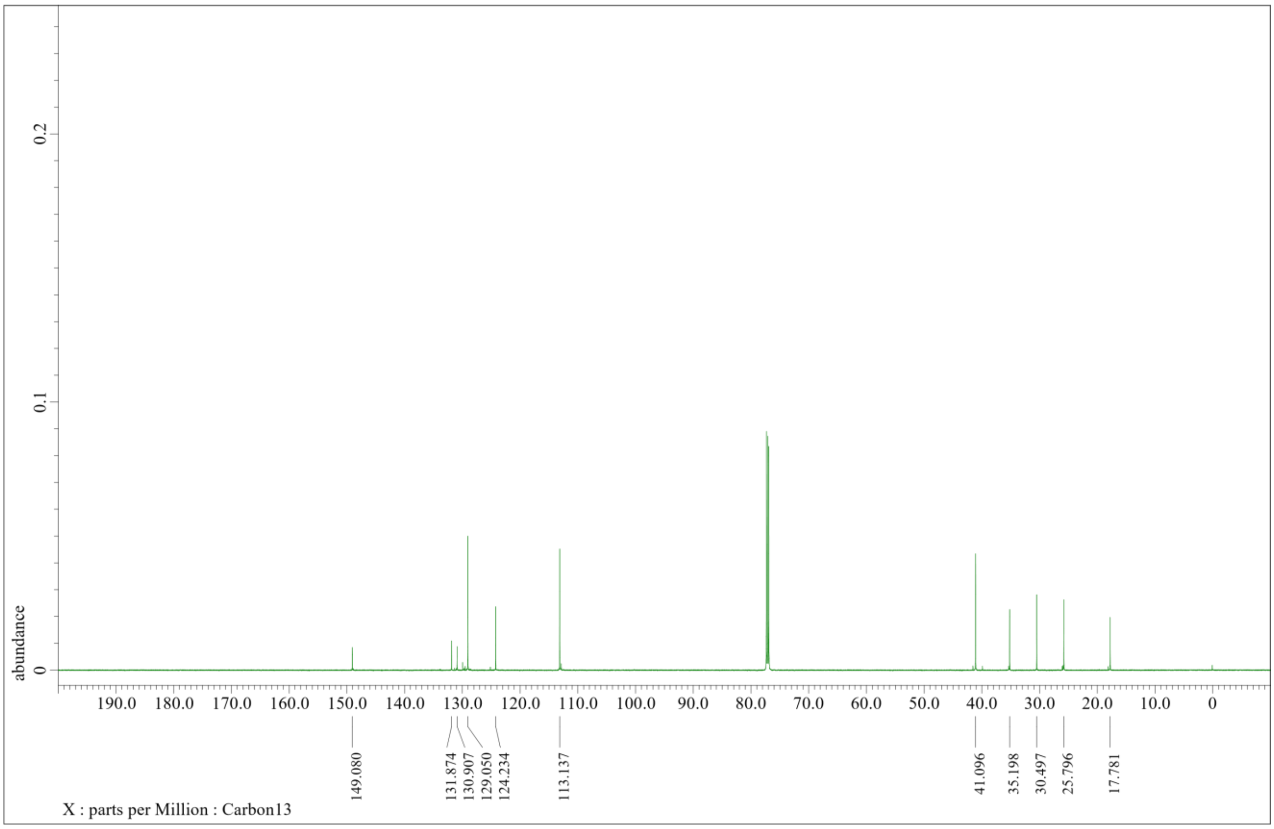

500 MHz, $CDCl_3$

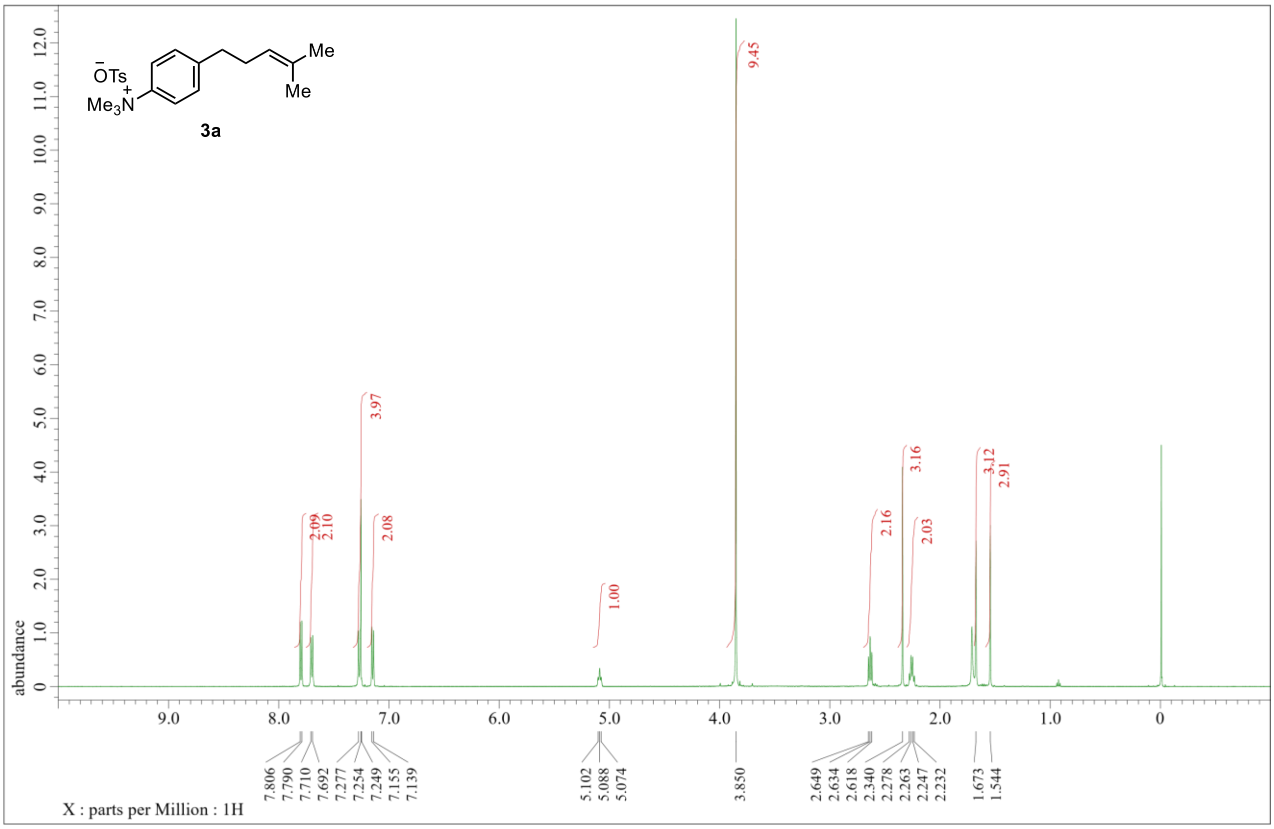

151 MHz, $CDCl_3$

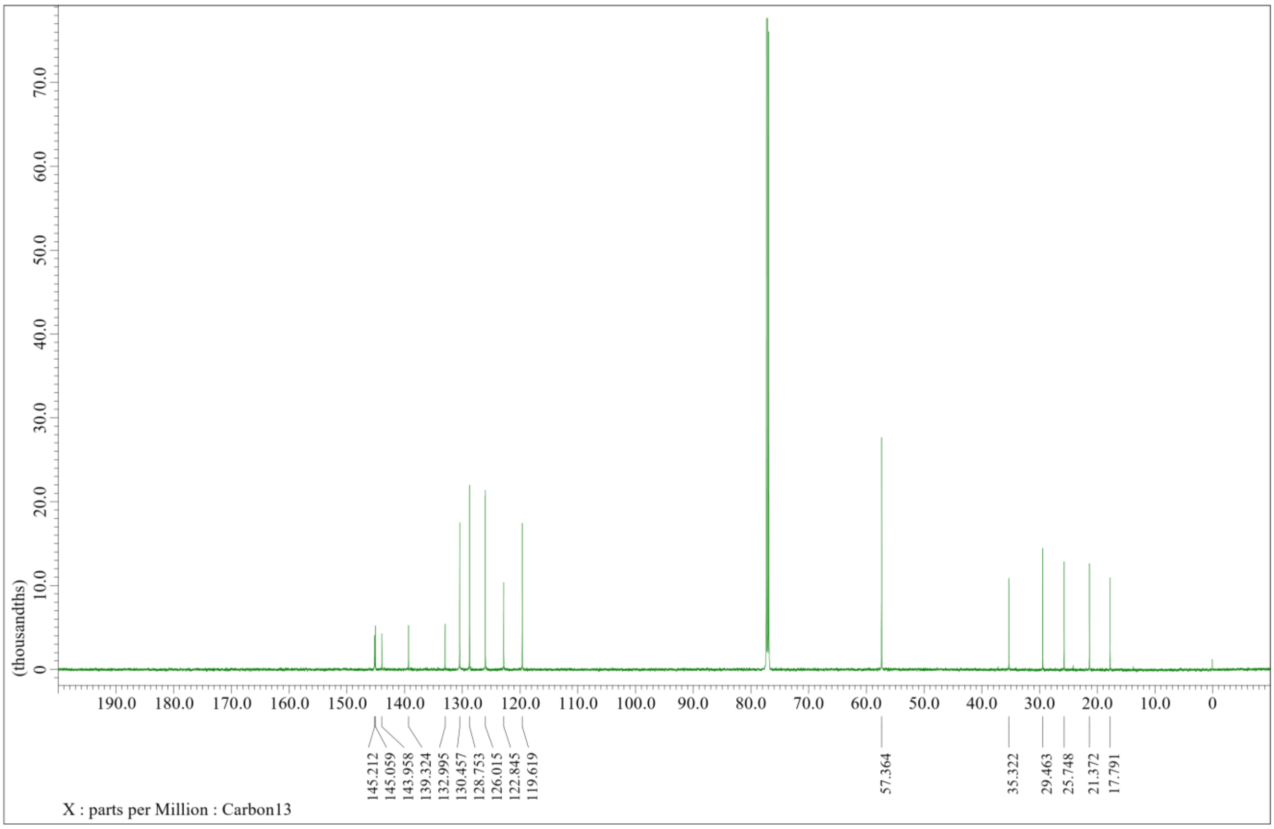

500 MHz, $CDCl_3$

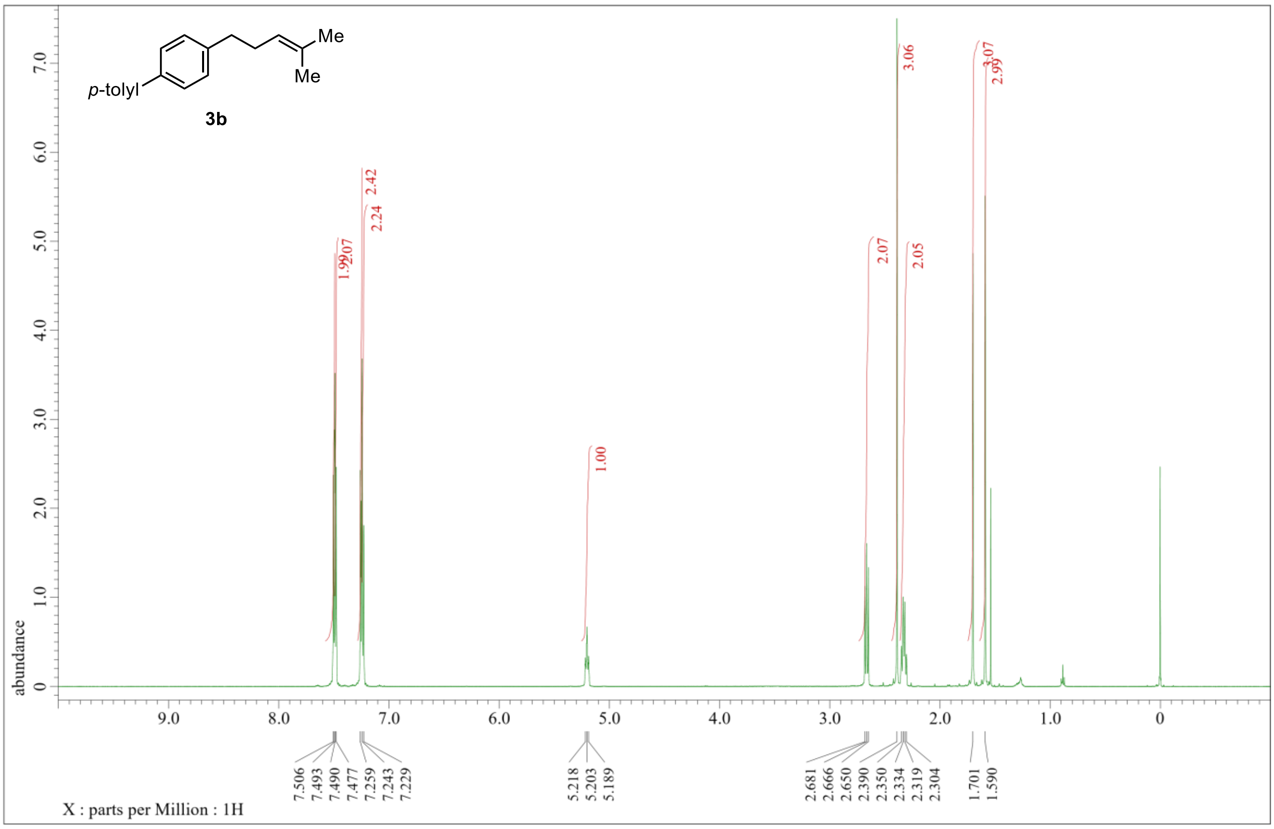

151 MHz, $CDCl_3$

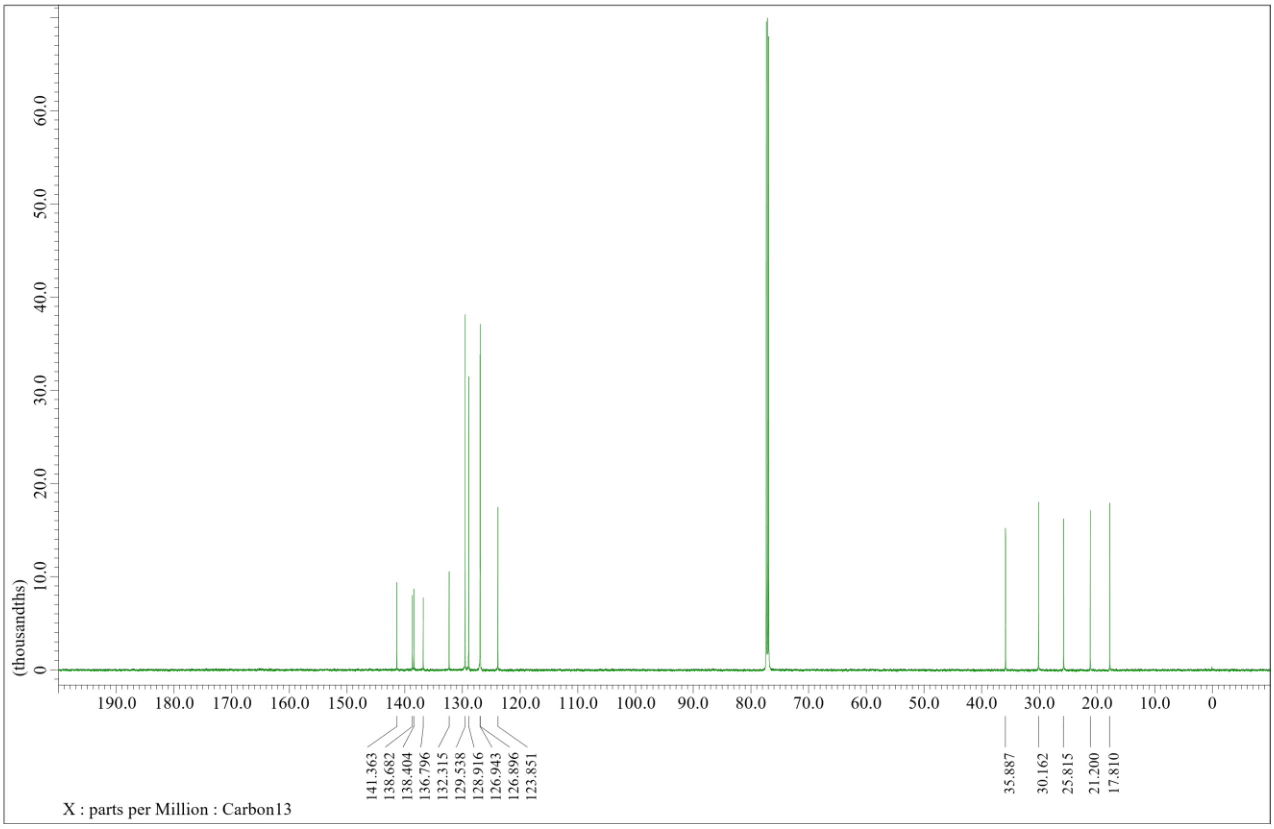

500 MHz, CDCl₃

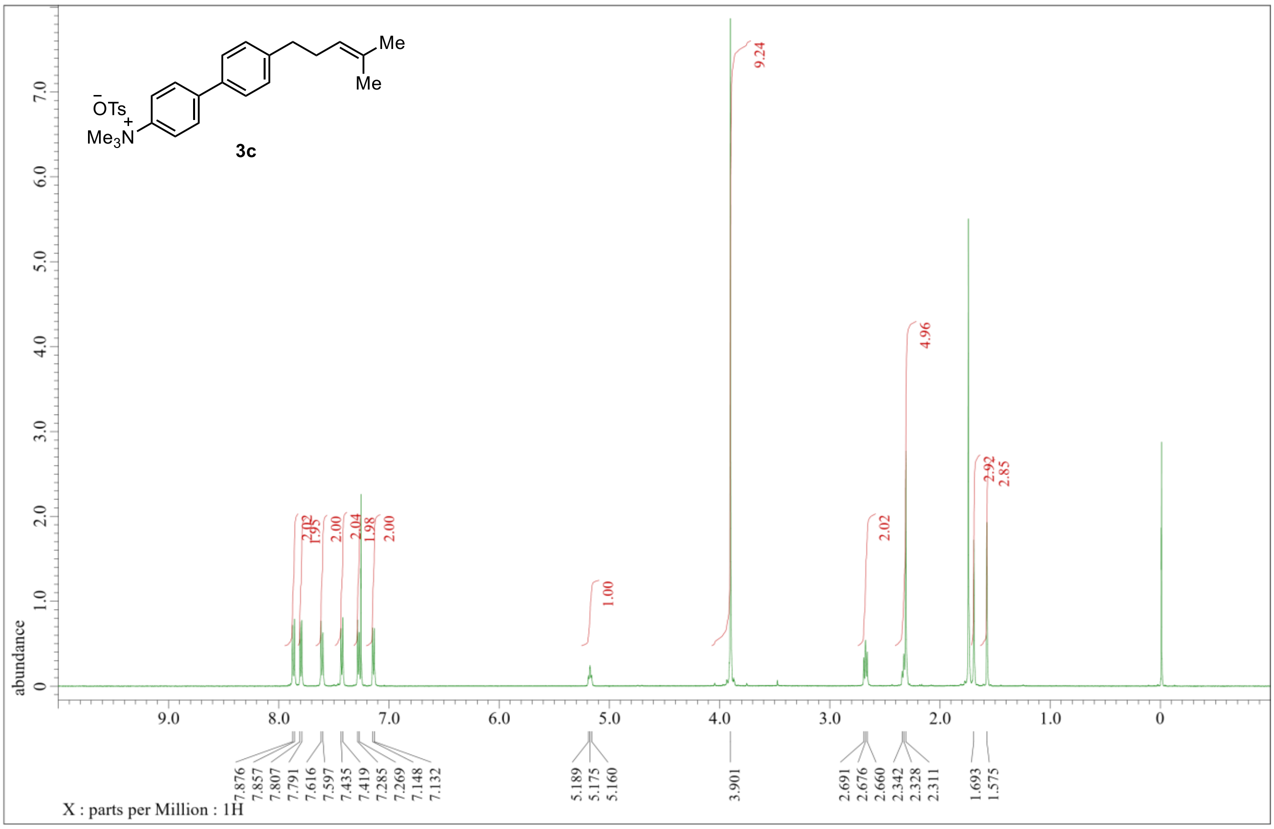

151 MHz, CDCl₃

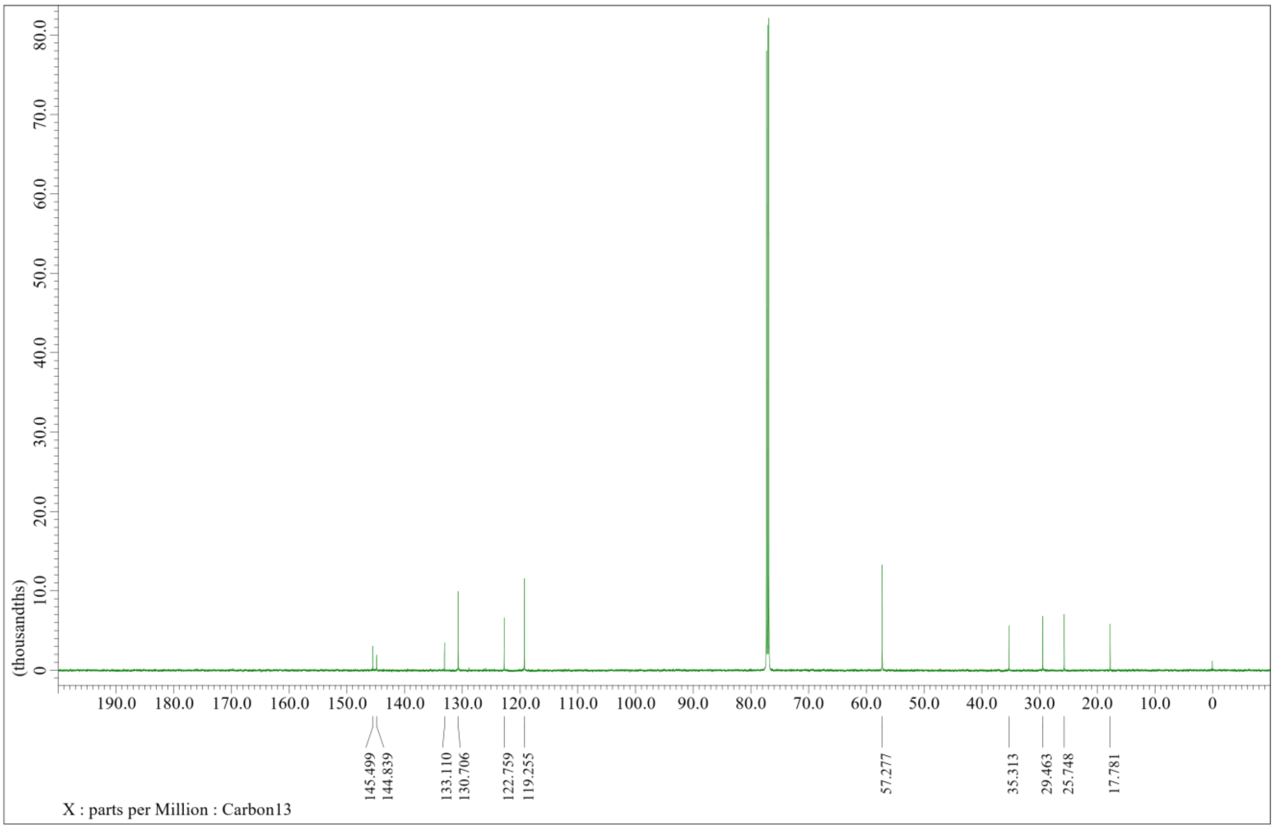

600 MHz, $CDCl_3$

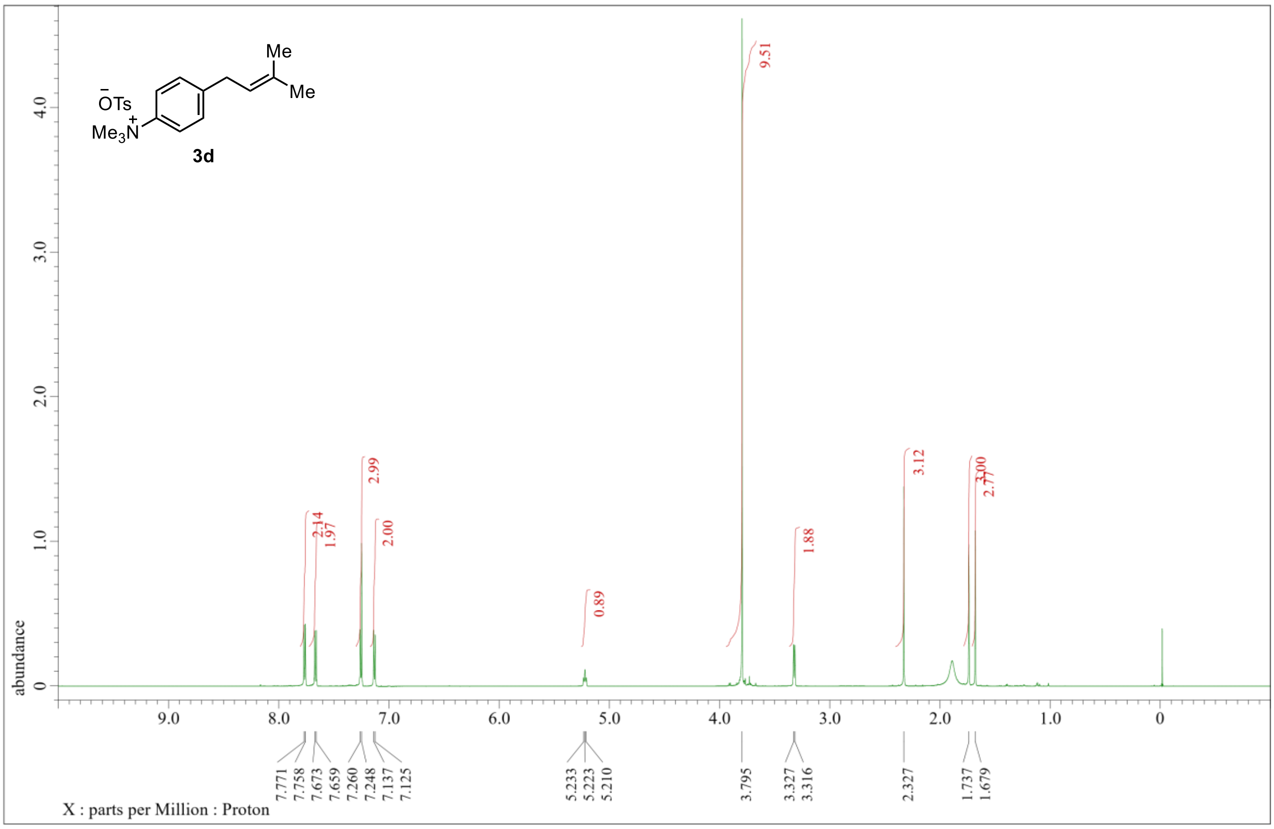

151 MHz, $CDCl_3$

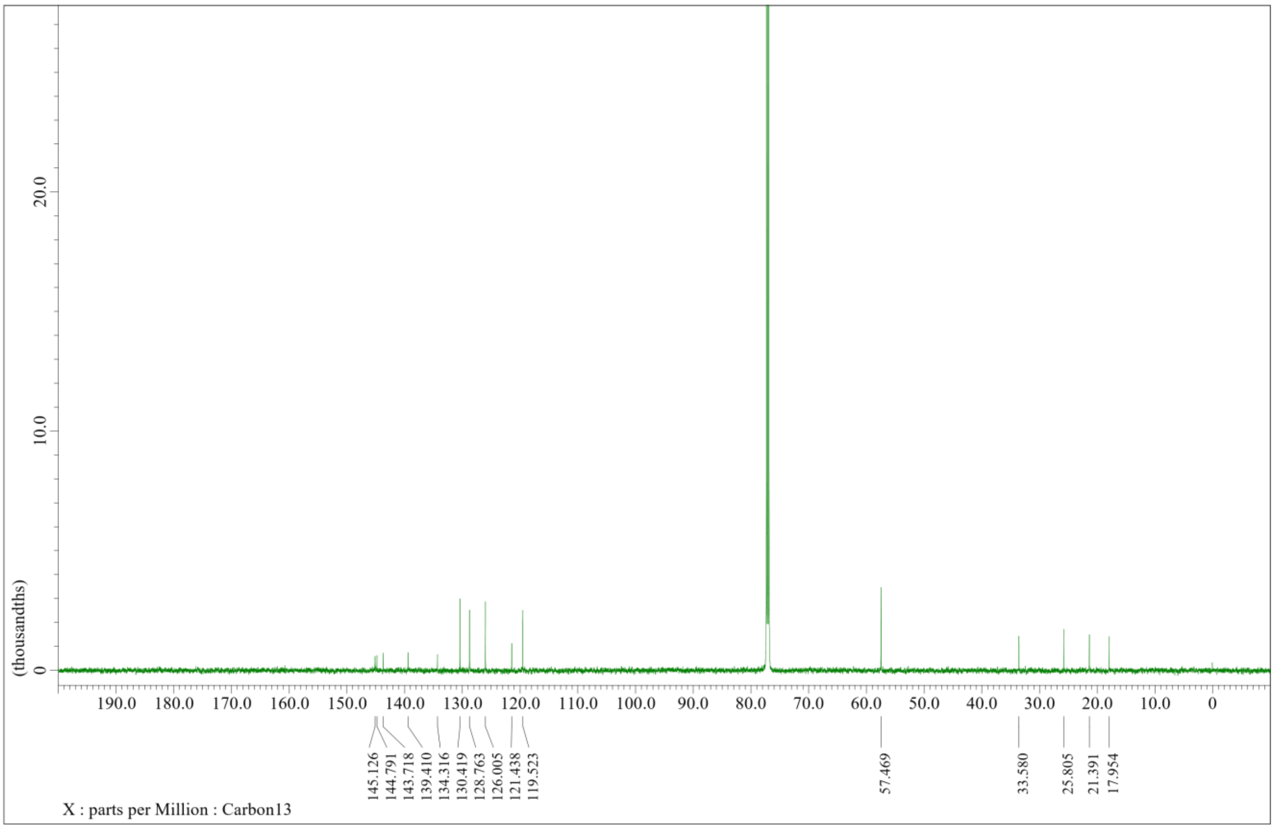

600 MHz, $CDCl_3$

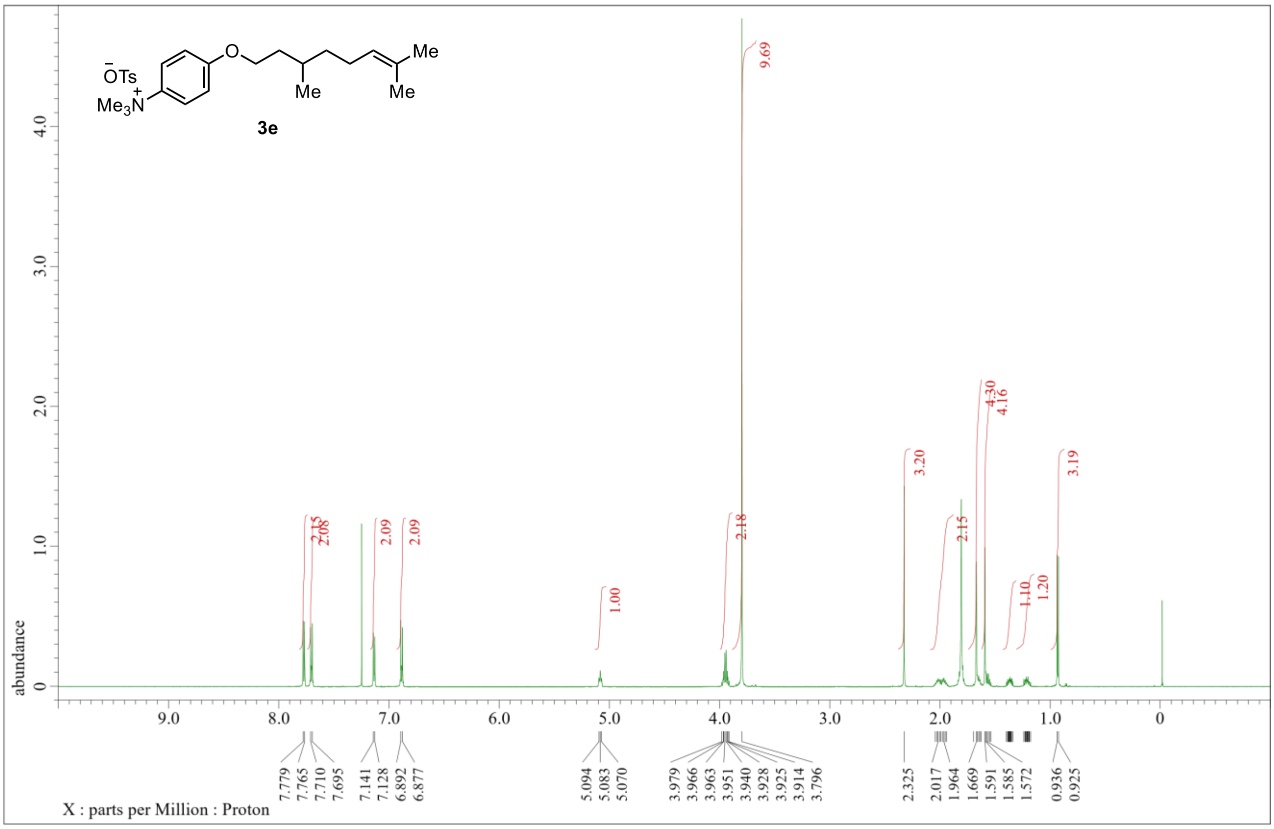

3e

151 MHz, $CDCl_3$

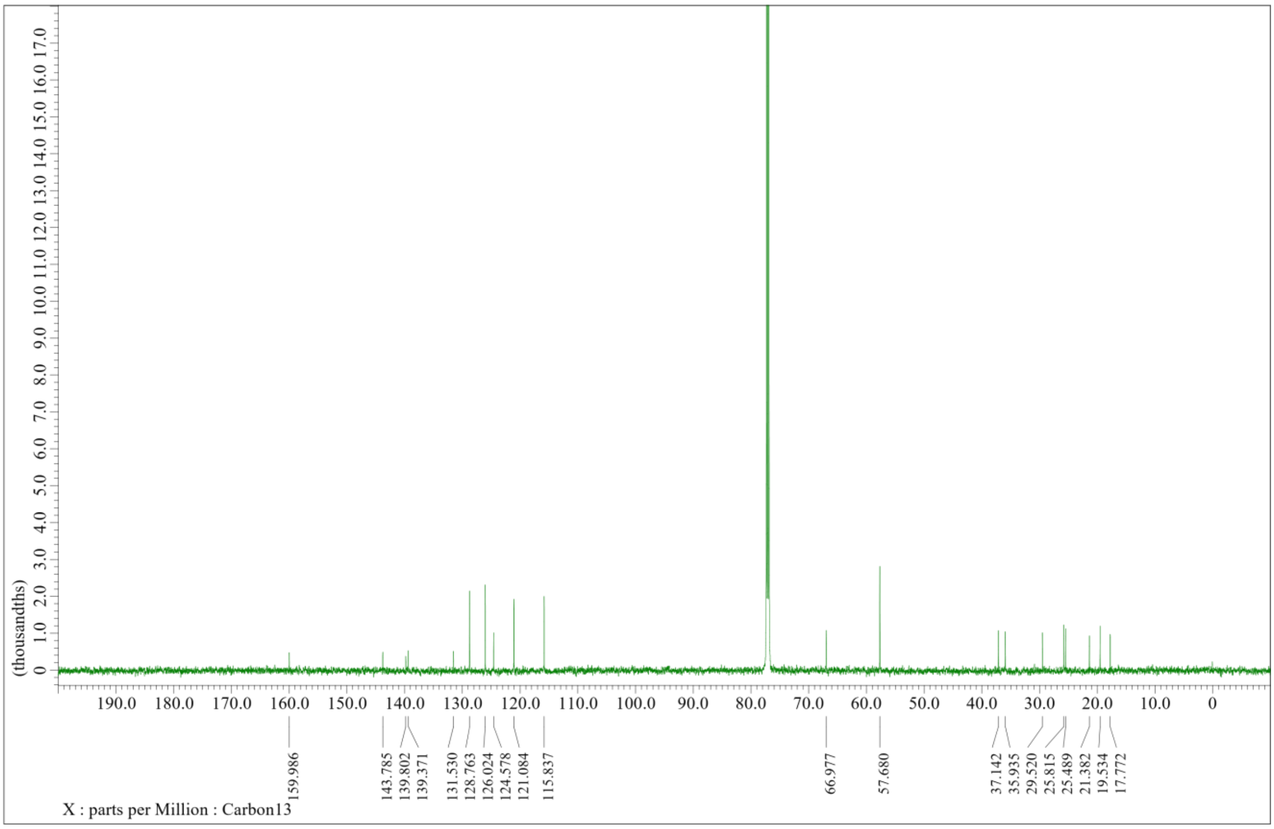

500 MHz, $CDCl_3$

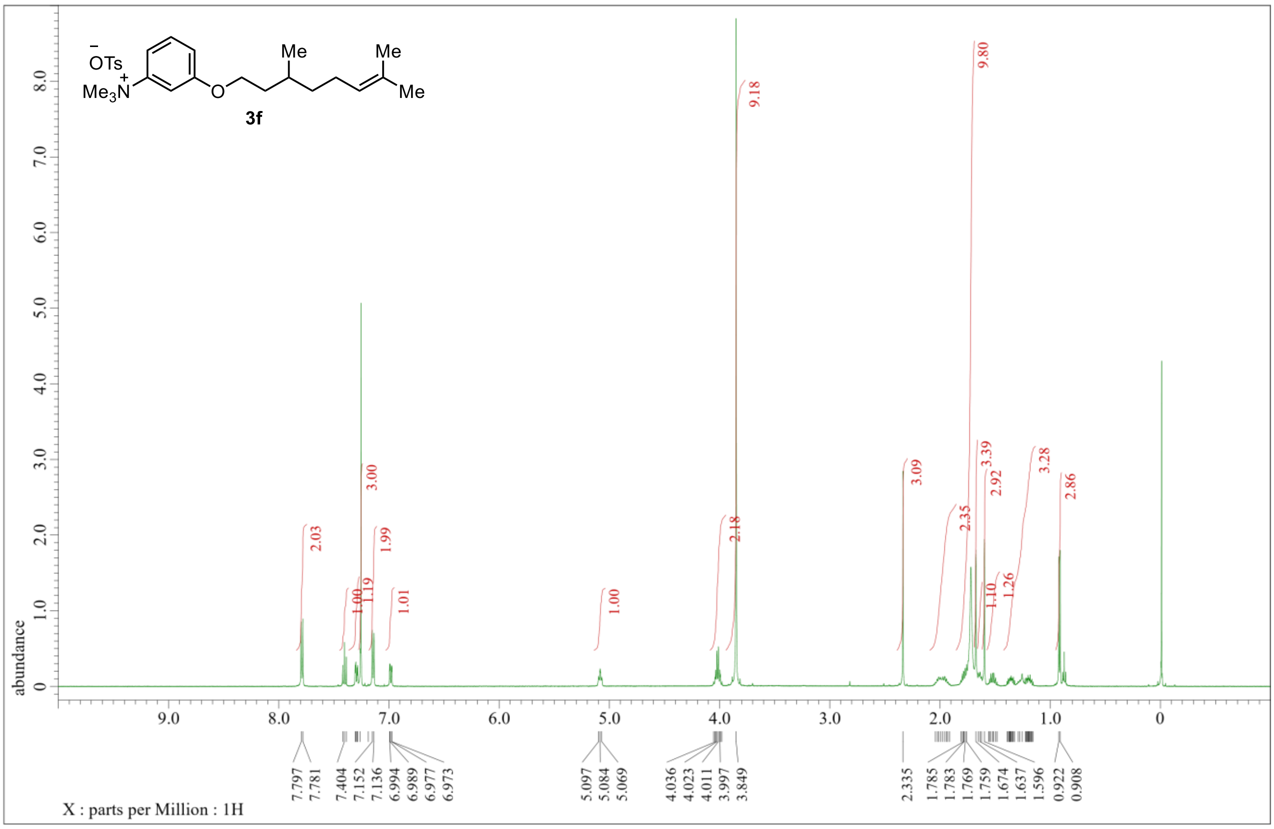

151 MHz, $CDCl_3$

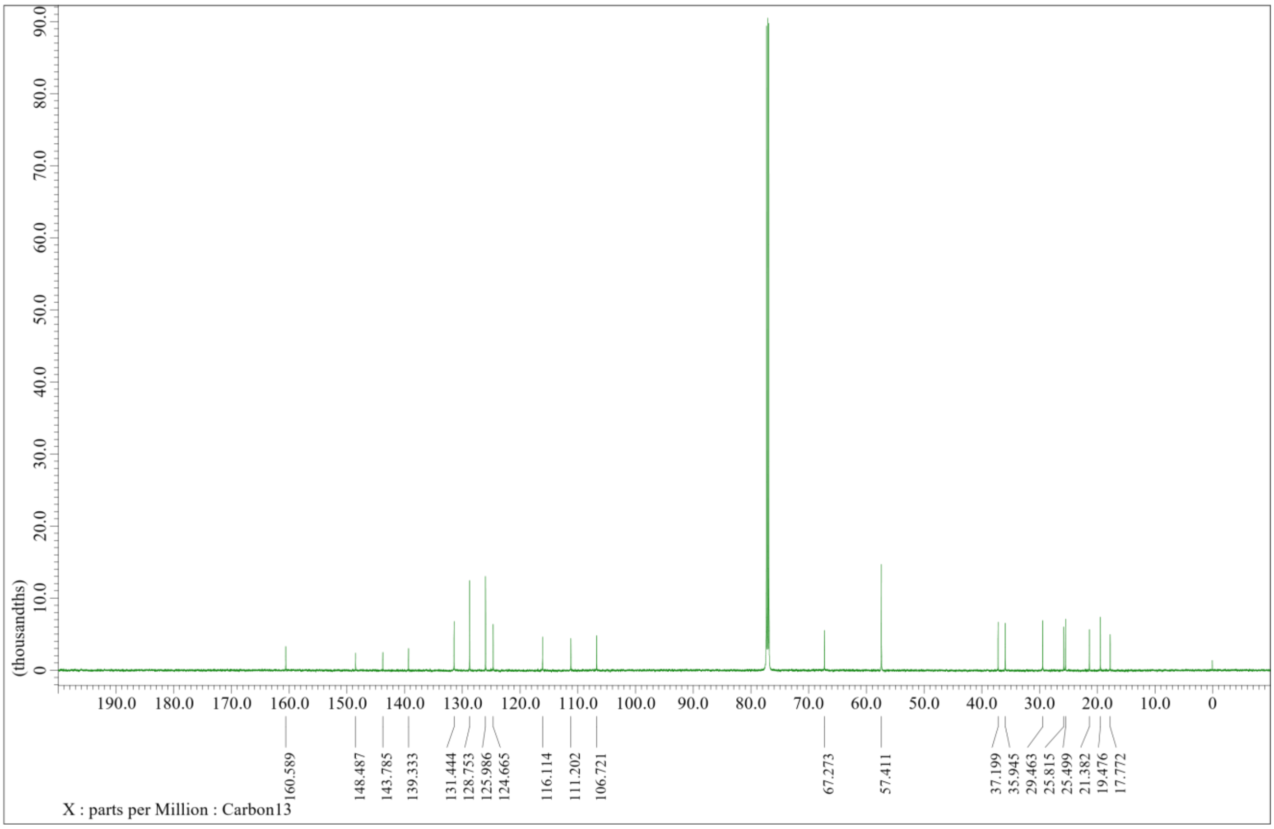

500 MHz, $CDCl_3$

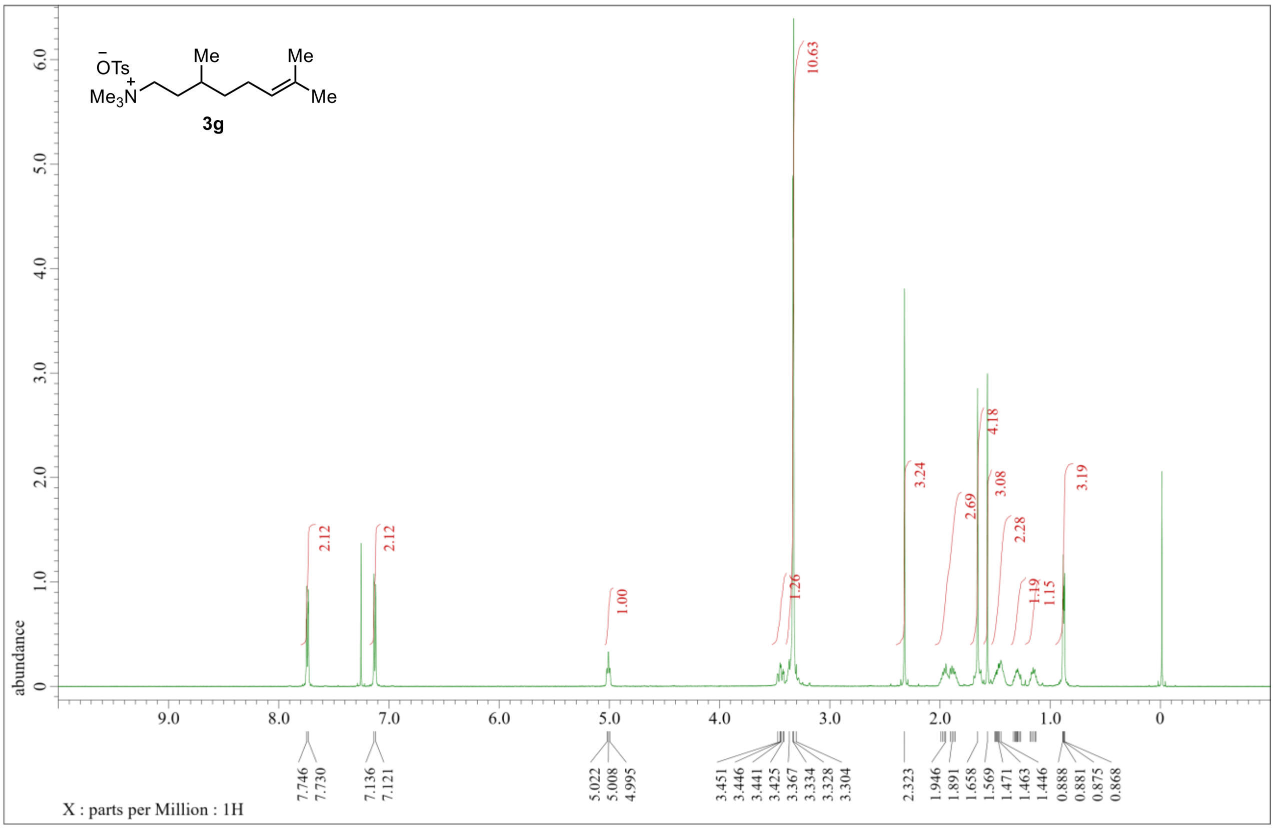

151 MHz, $CDCl_3$

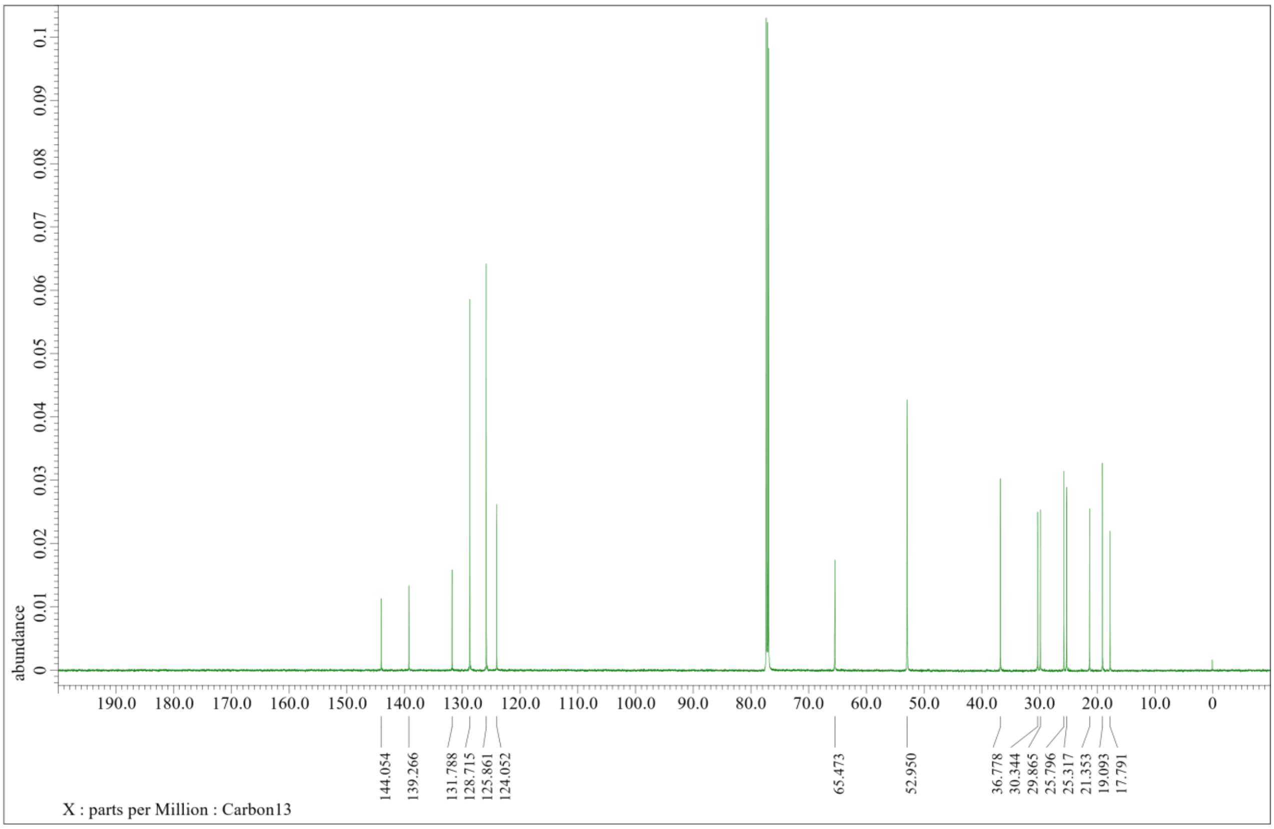

500 MHz, CDCl$_3$

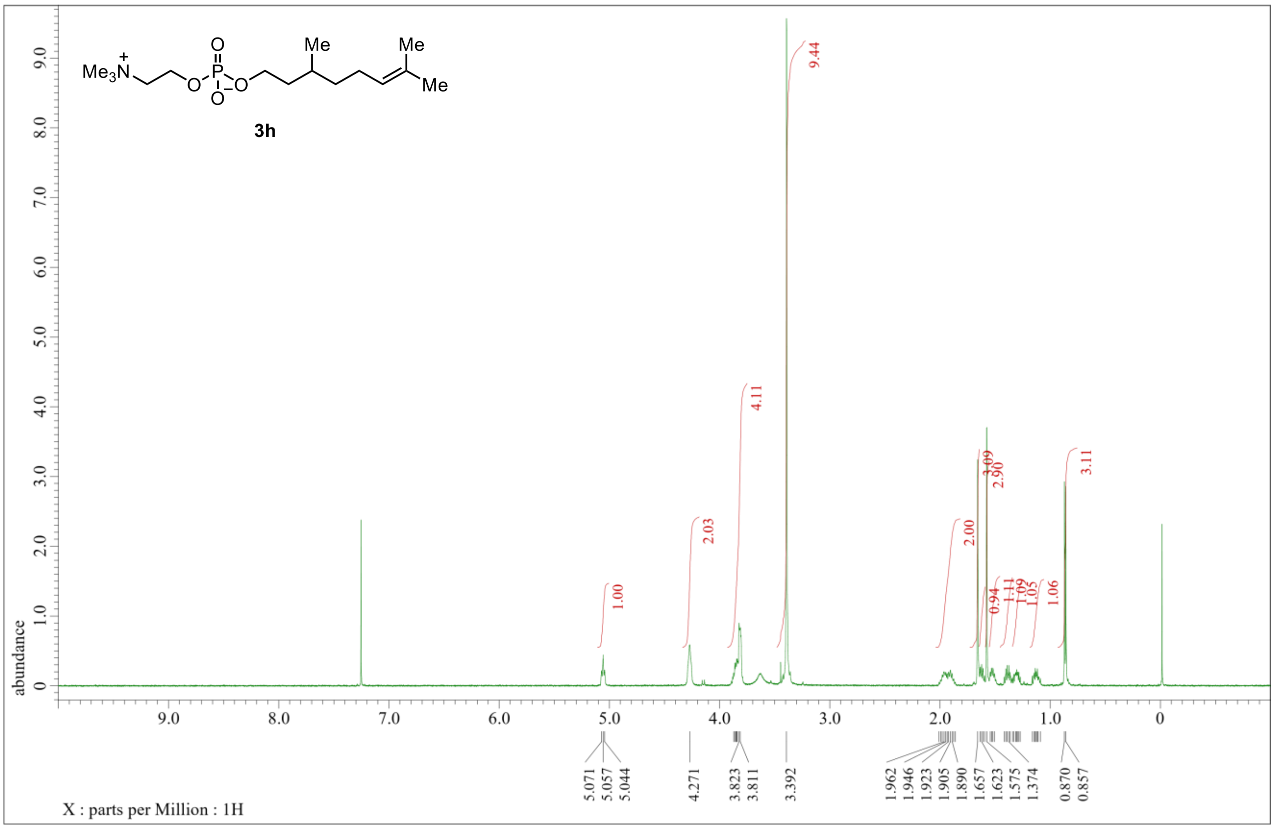

151 MHz, CDCl$_3$

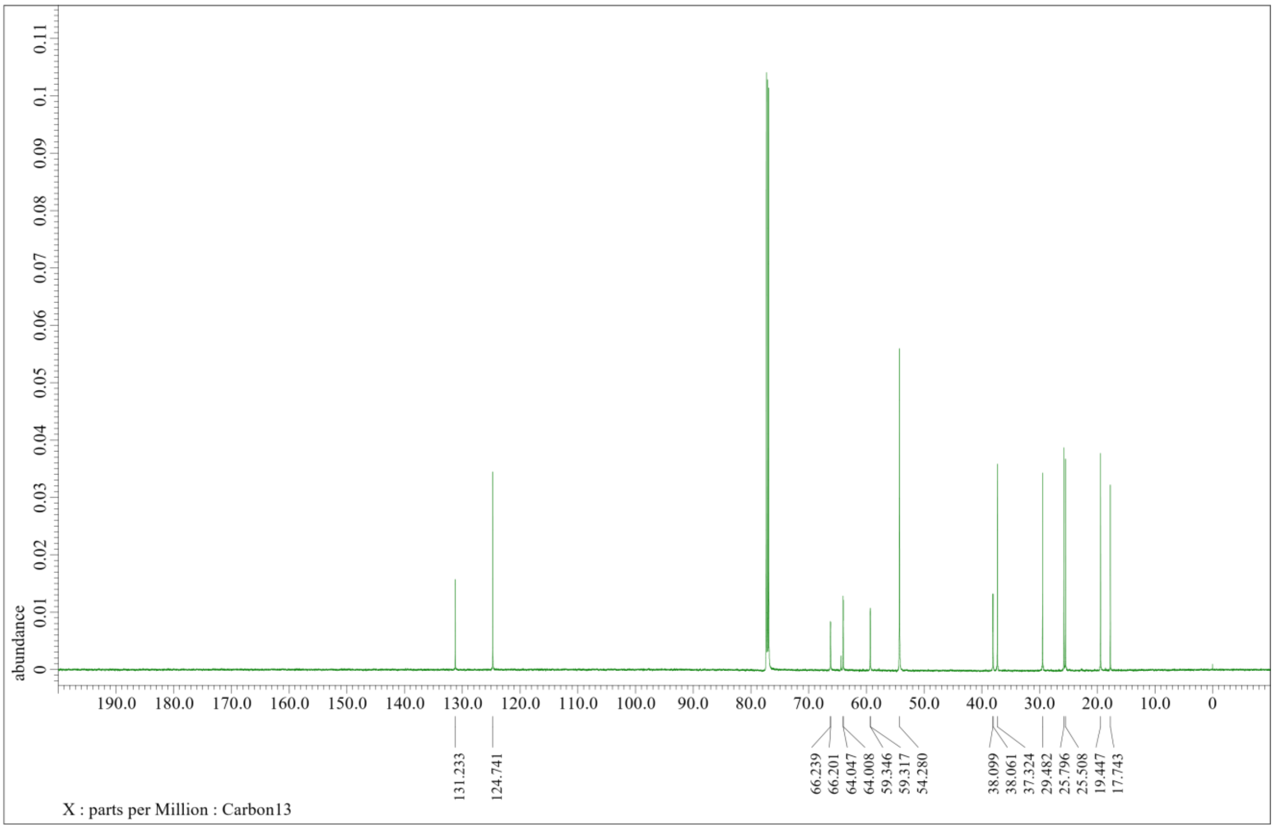

^{31}P NMR: 243 MHz, $CDCl_3$

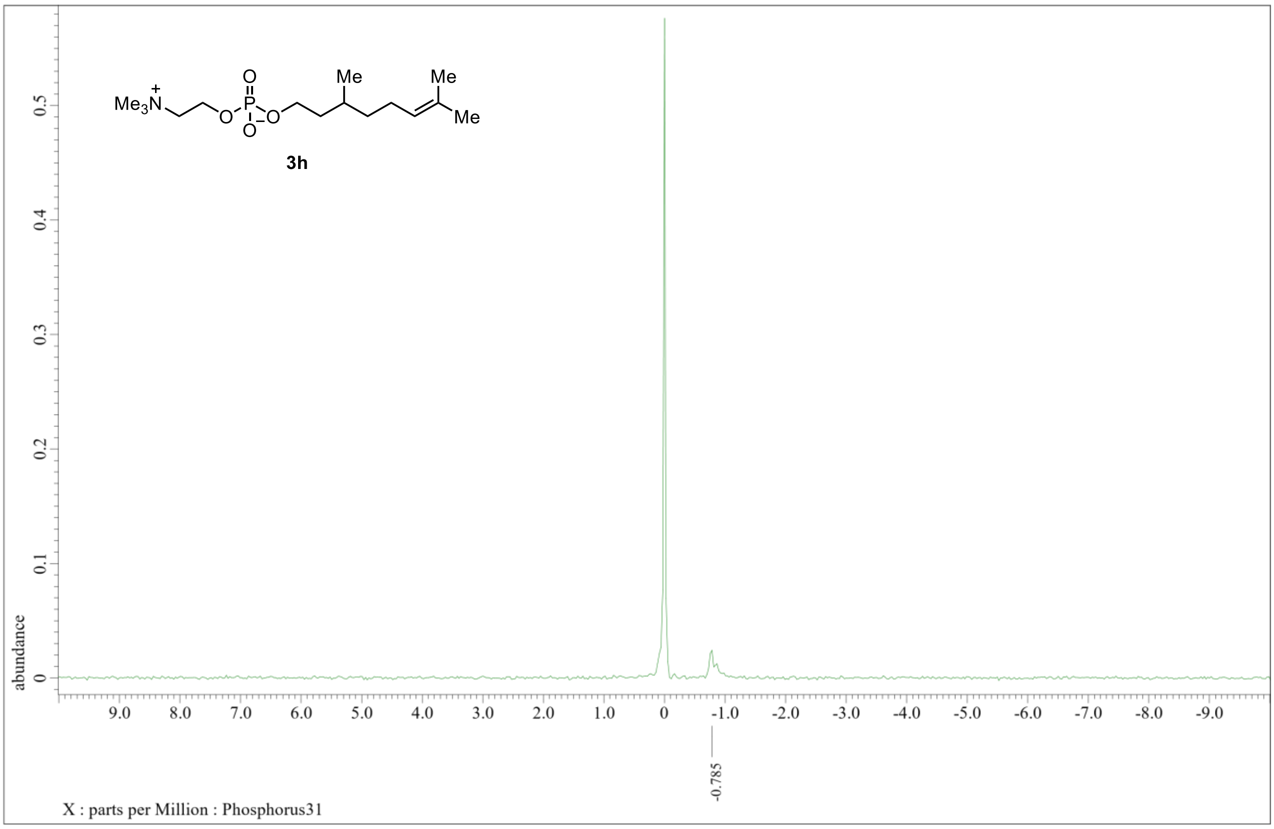

500 MHz, CDCl$_3$

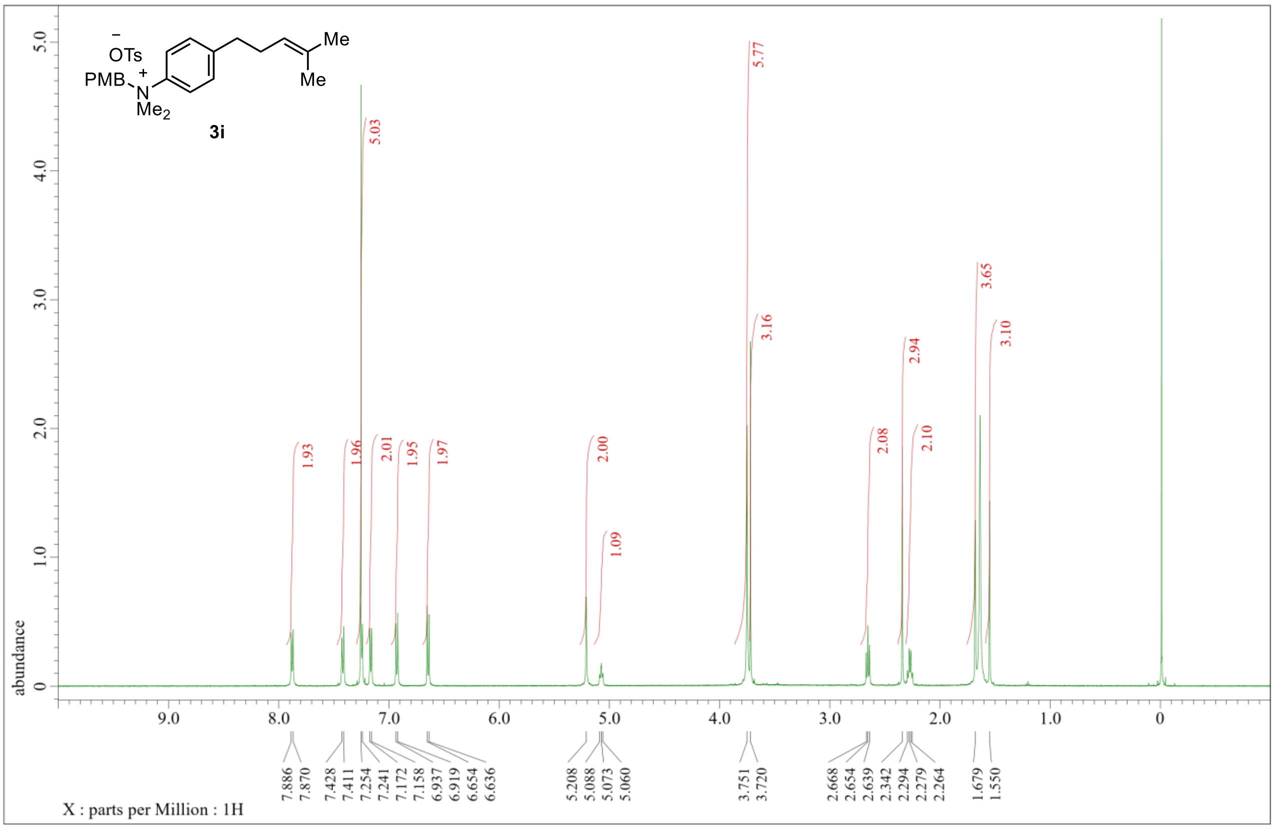

151 MHz, CDCl$_3$

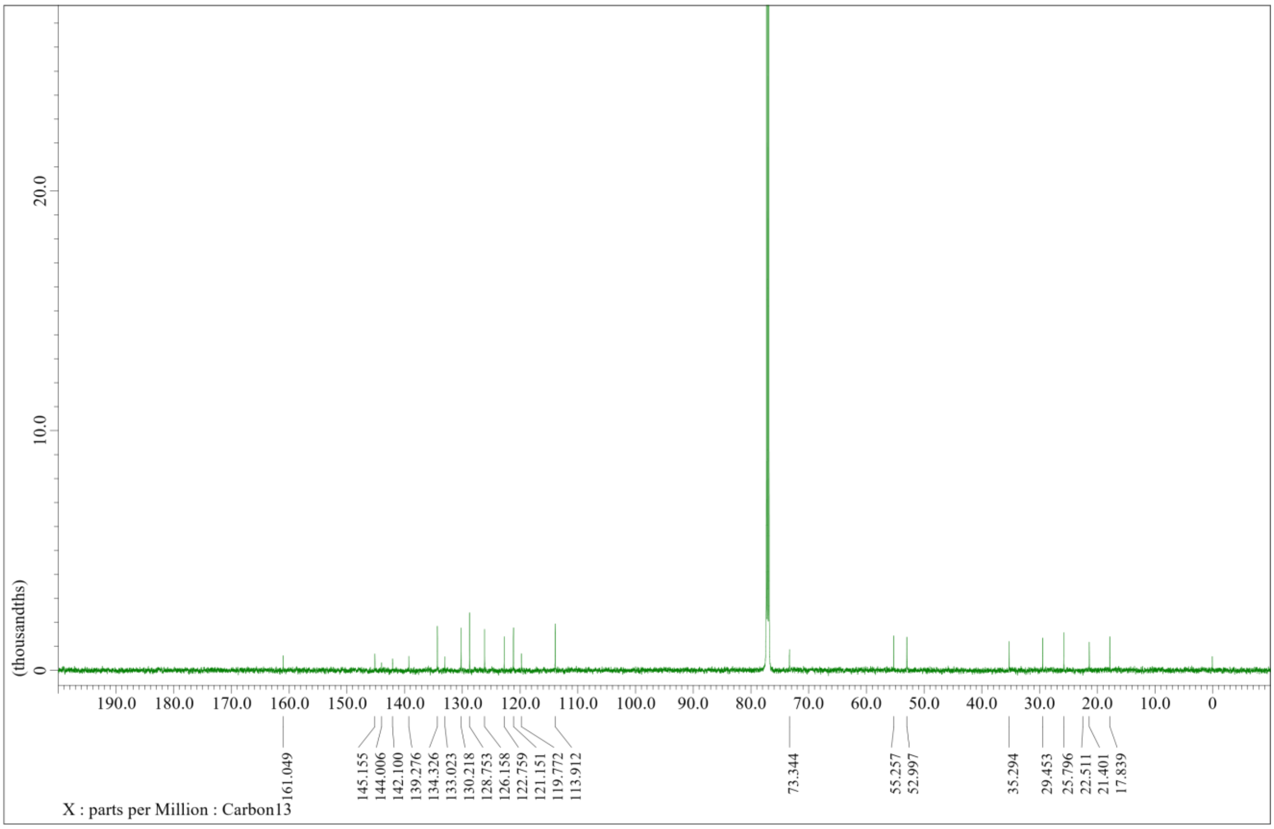

500 MHz, $CDCl_3$

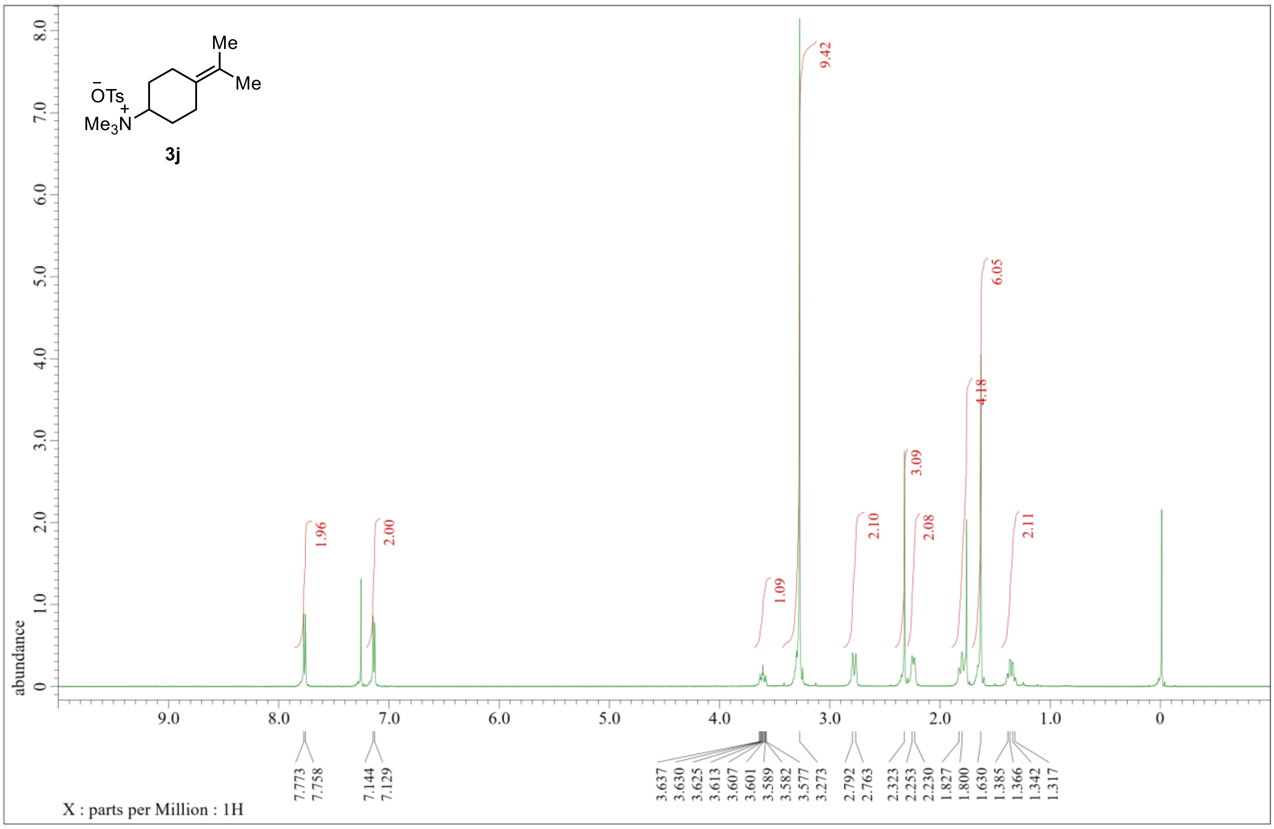

151 MHz, $CDCl_3$

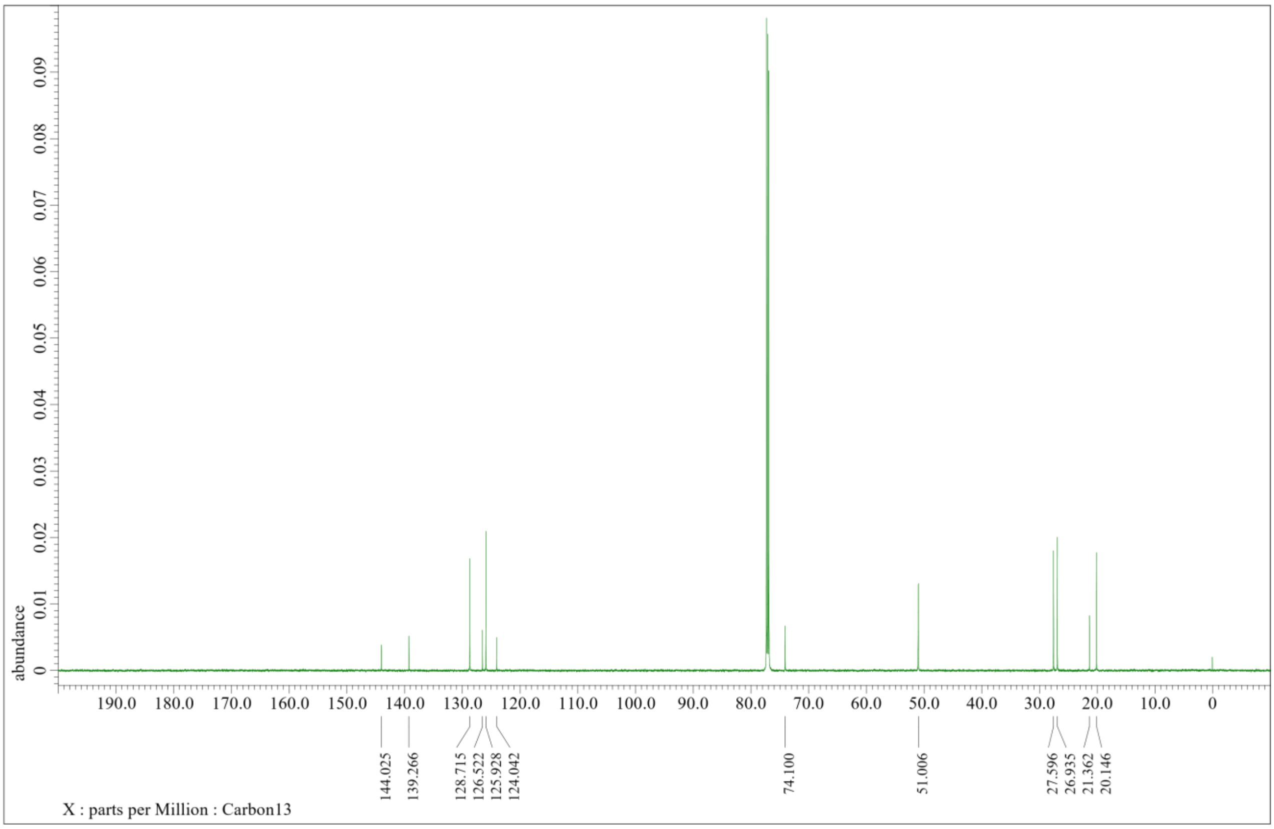

500 MHz, $CDCl_3$

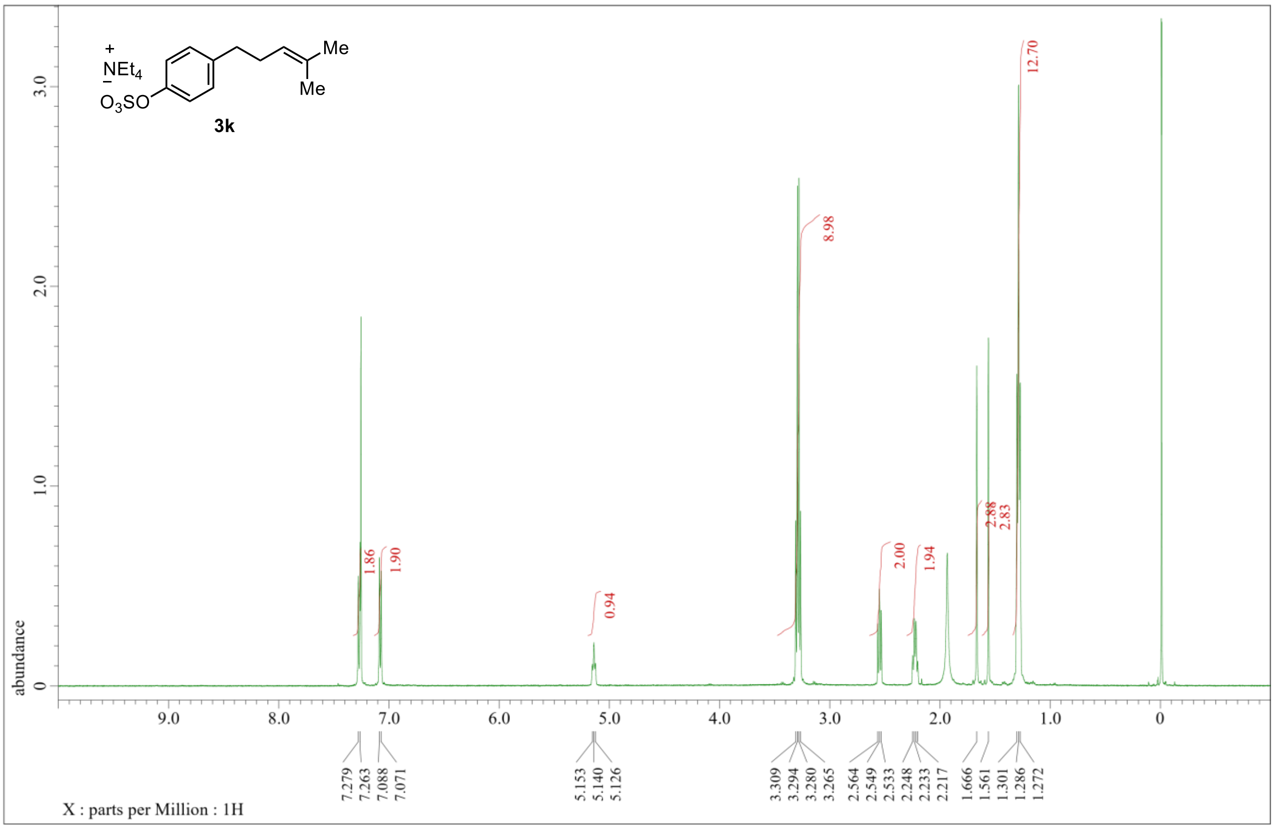

151 MHz, $CDCl_3$

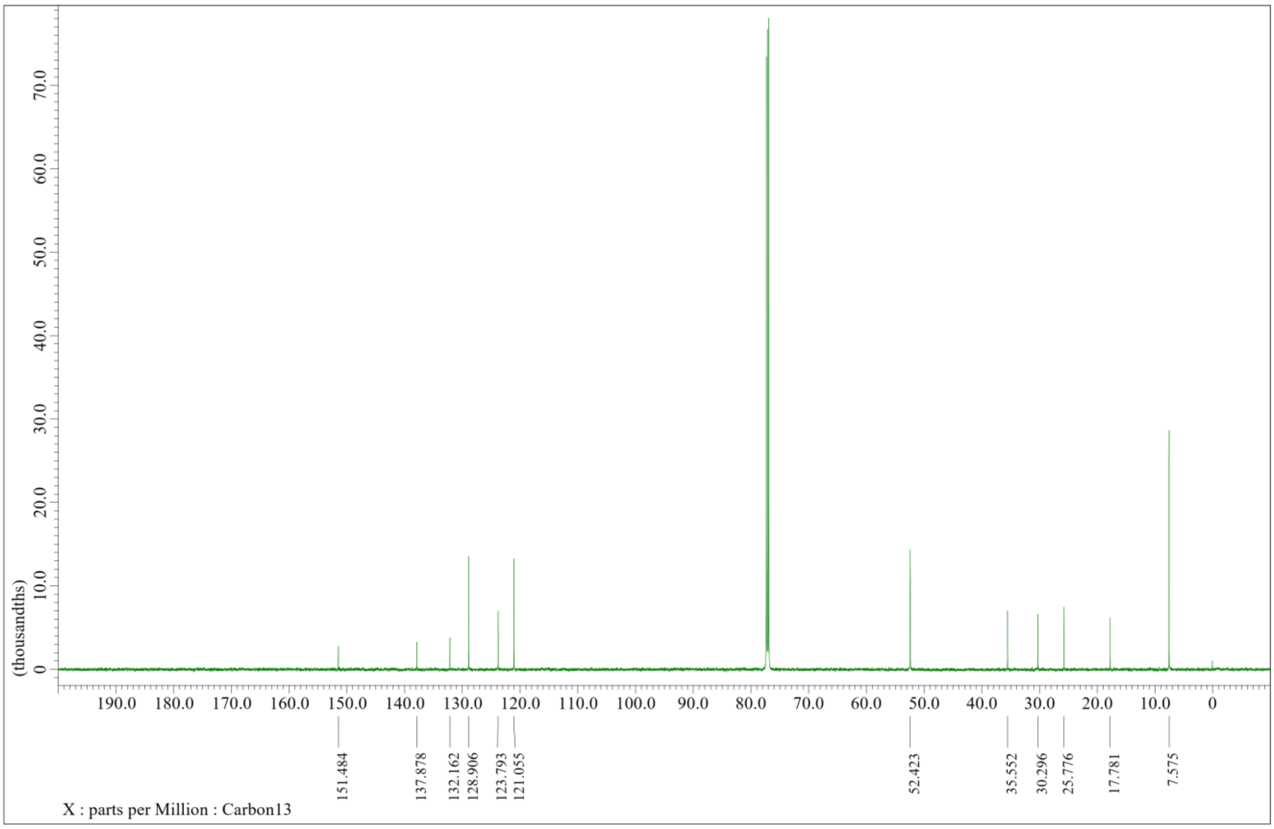

600 MHz, $CDCl_3$

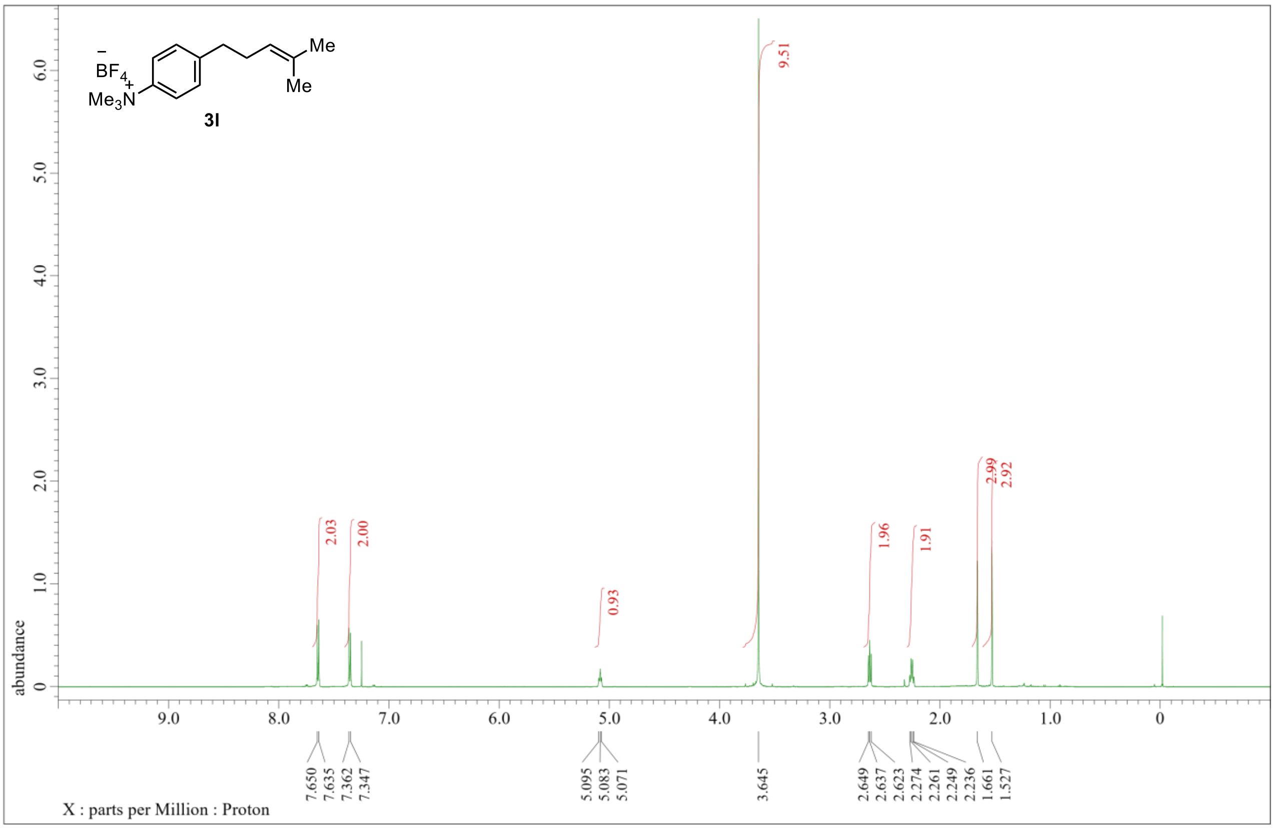

151 MHz, $CDCl_3$

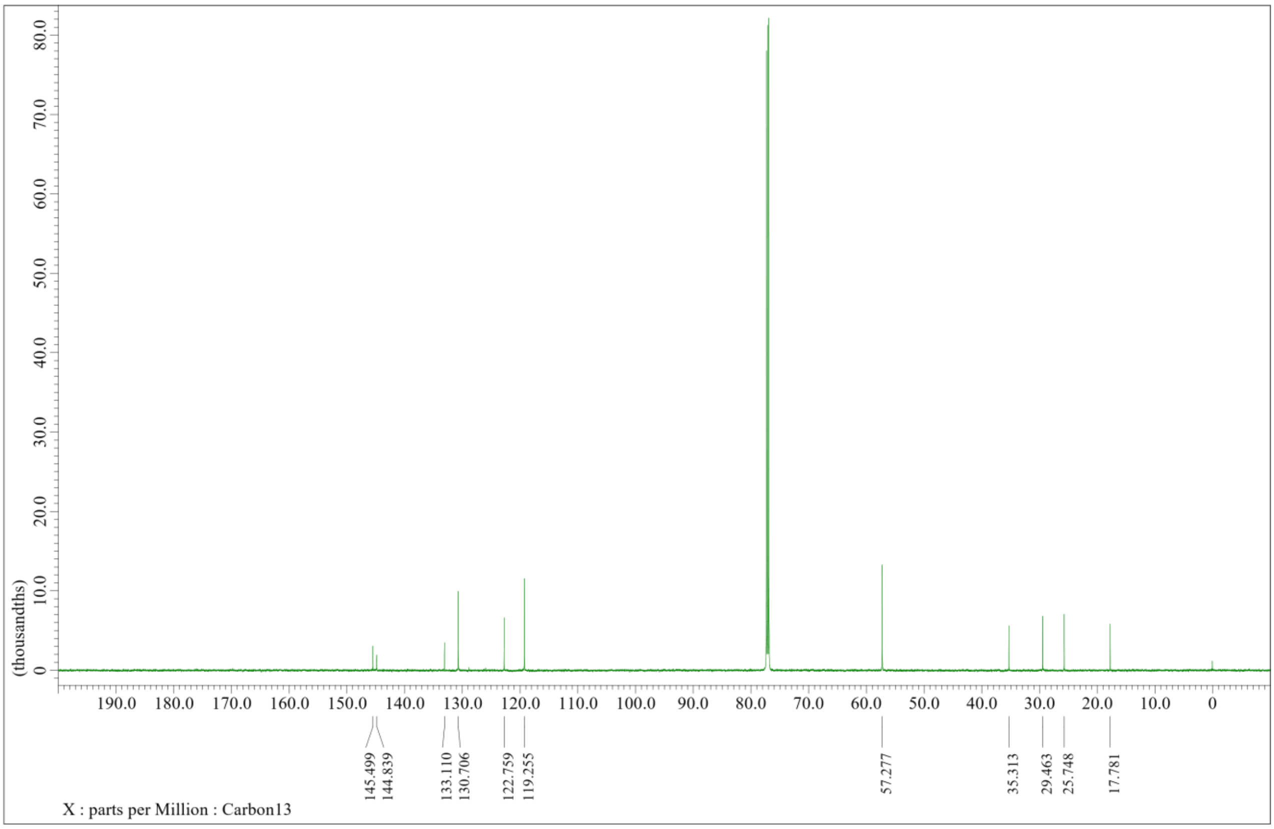

^{19}F NMR: 471 MHz, $CDCl_3$

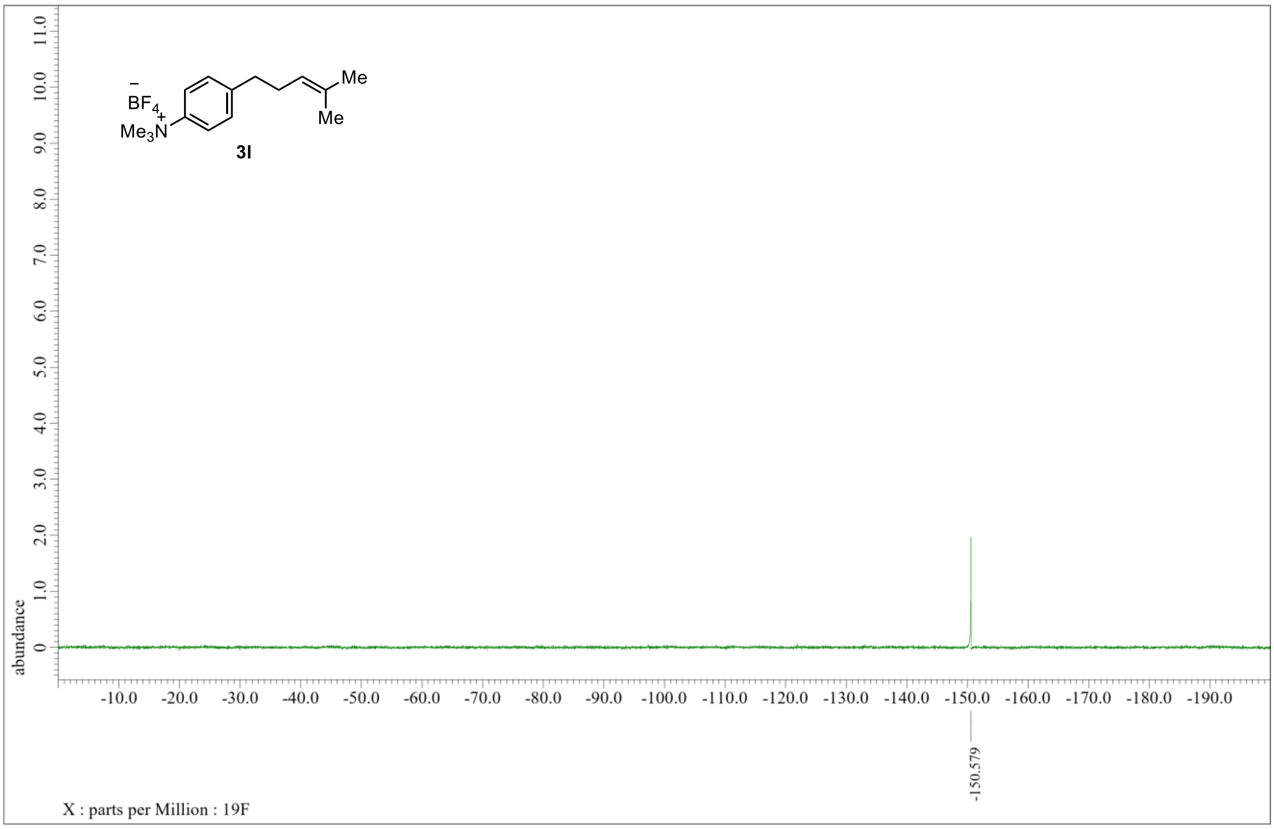

600 MHz, $CDCl_3$

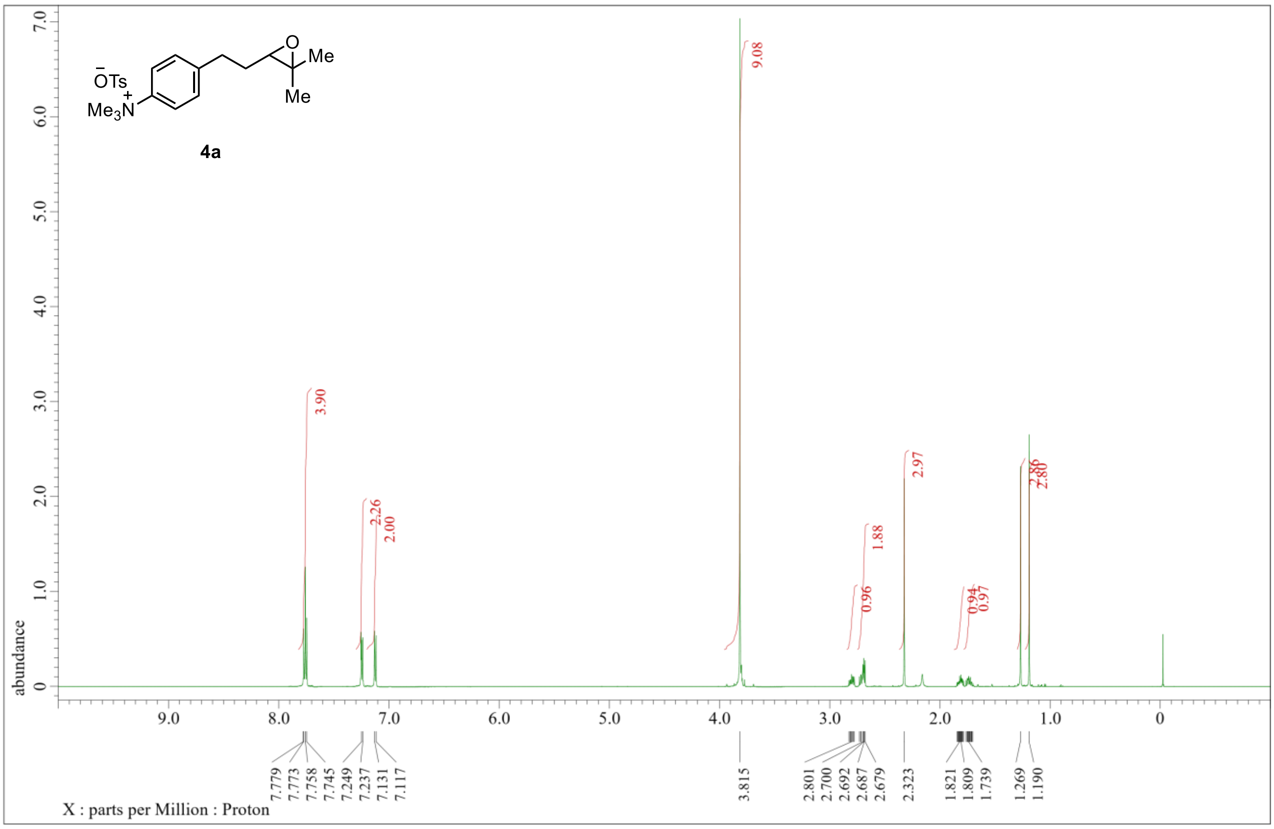

151 MHz, $CDCl_3$

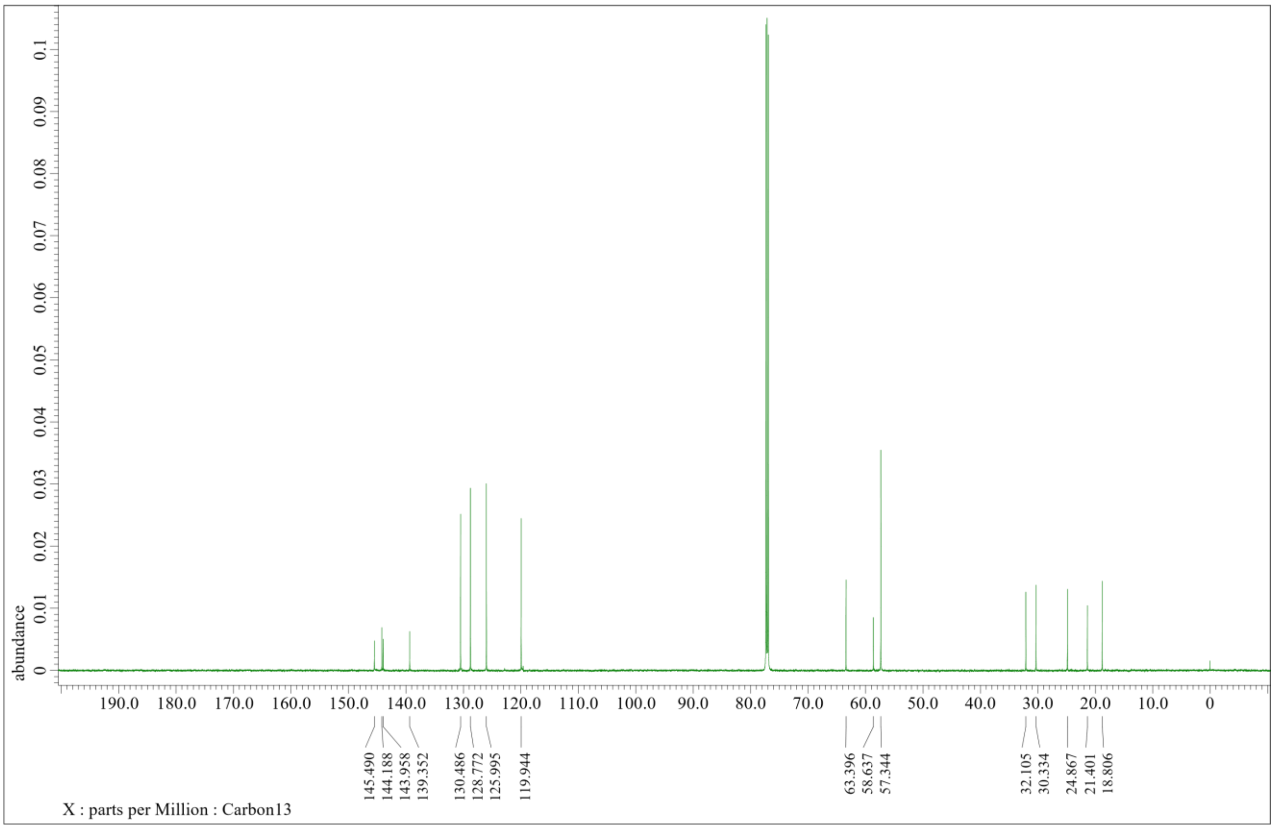

600 MHz, $CDCl_3$

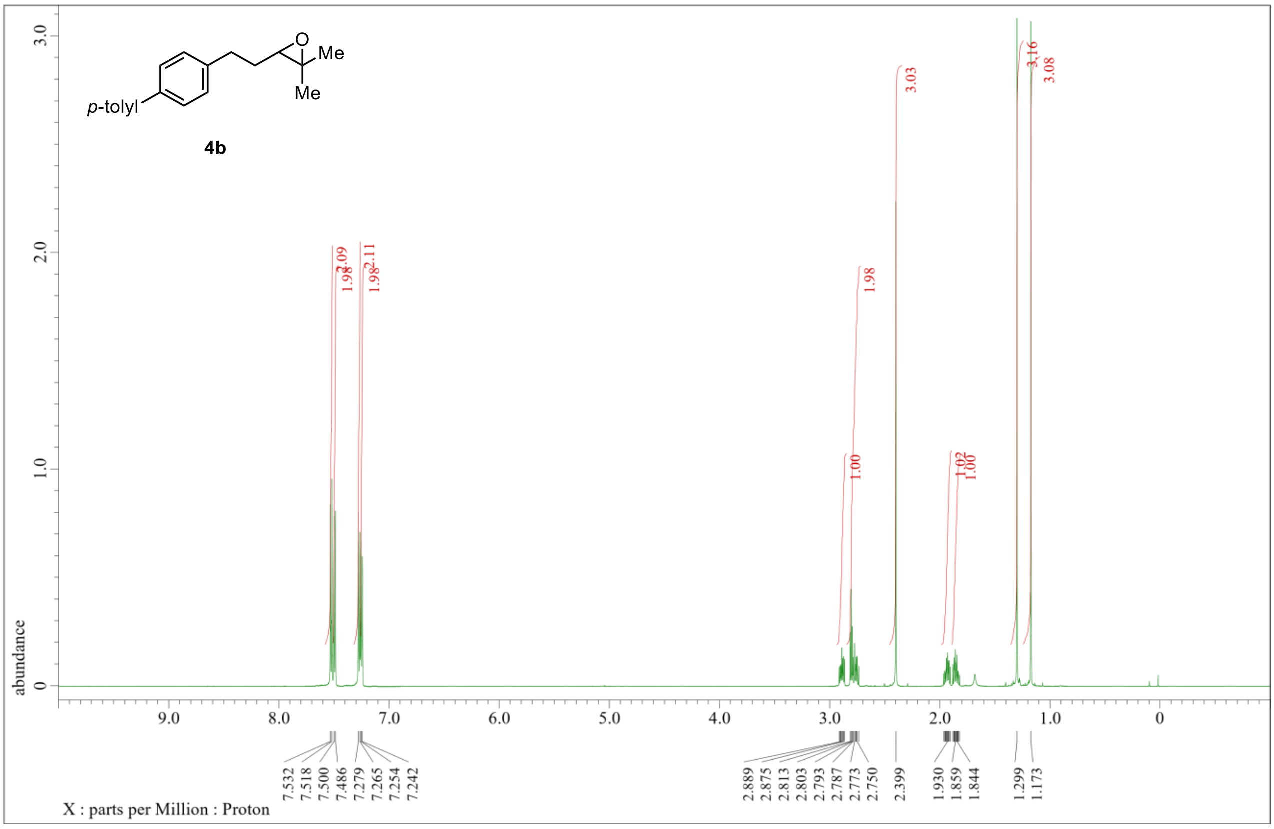

151 MHz, $CDCl_3$

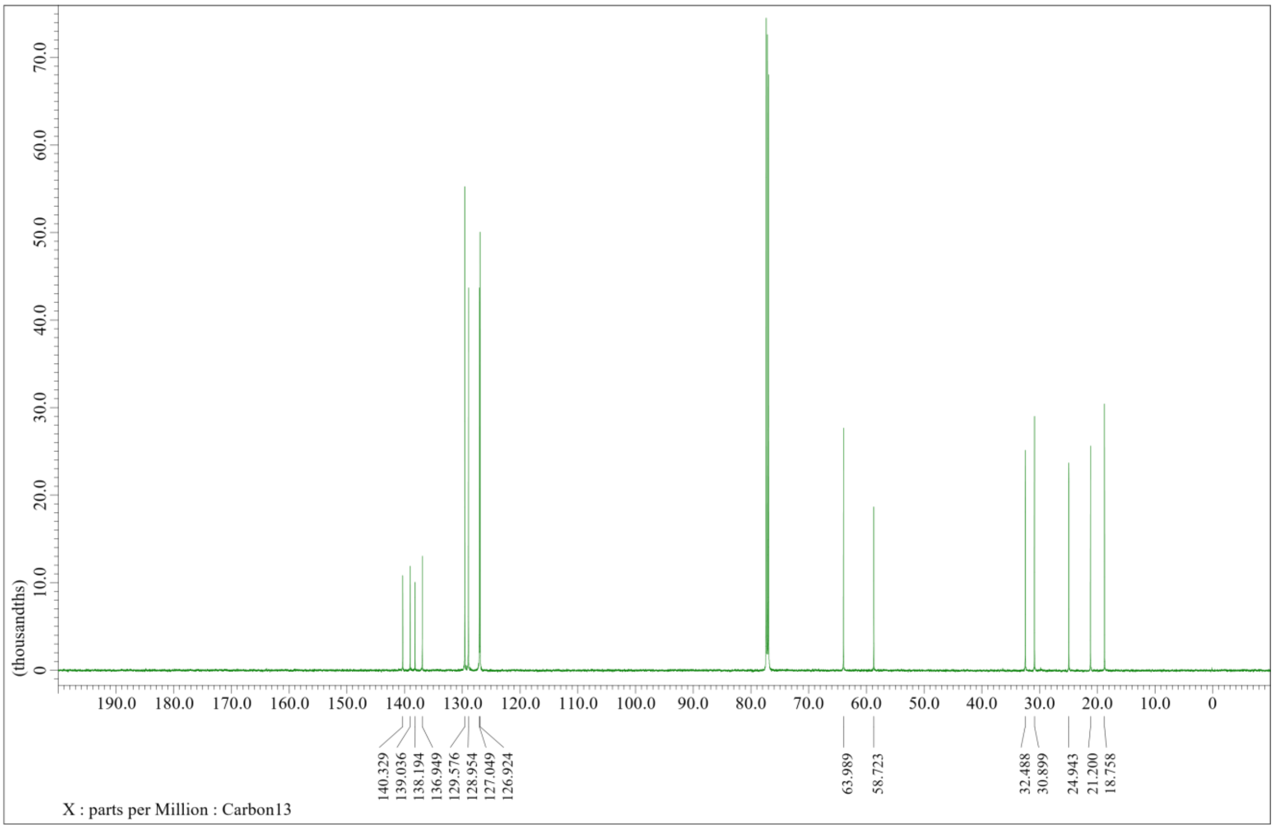

500 MHz, $CDCl_3$

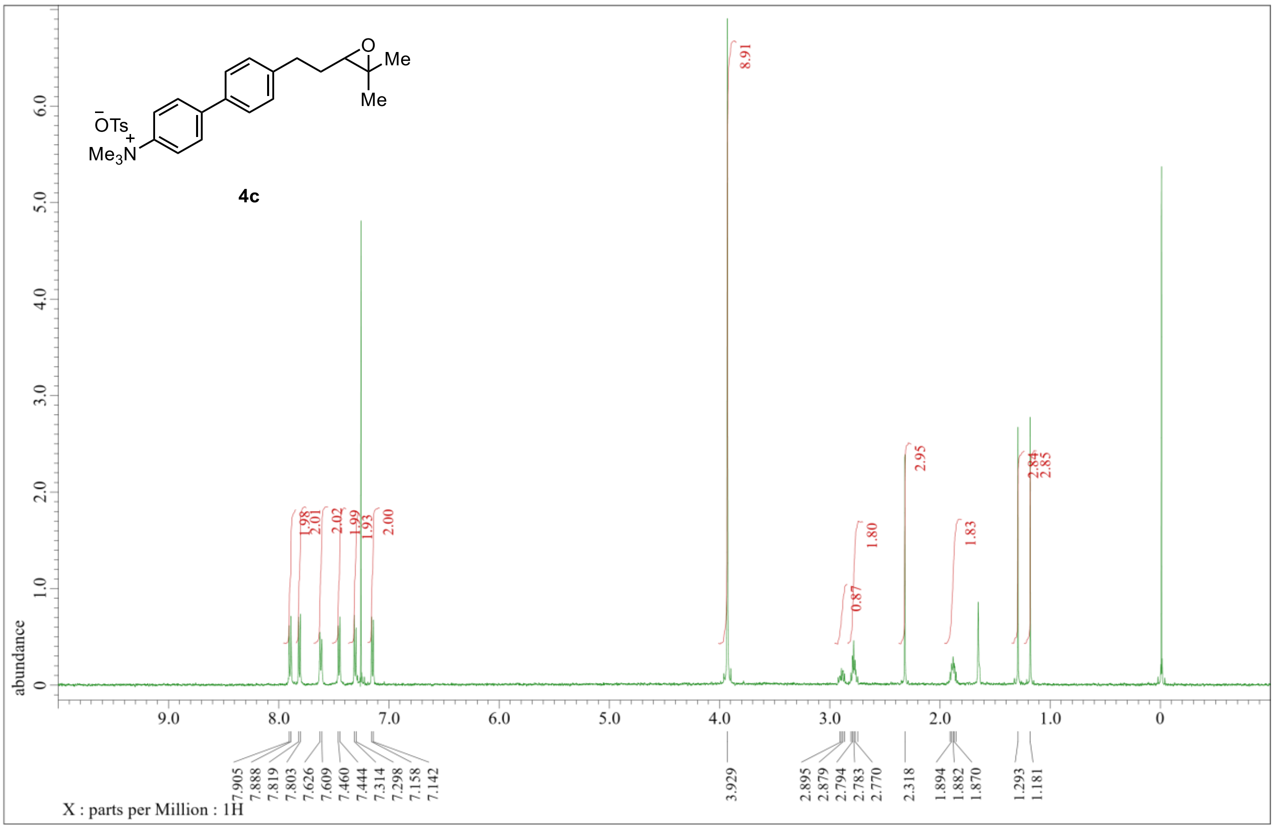

151 MHz, $CDCl_3$

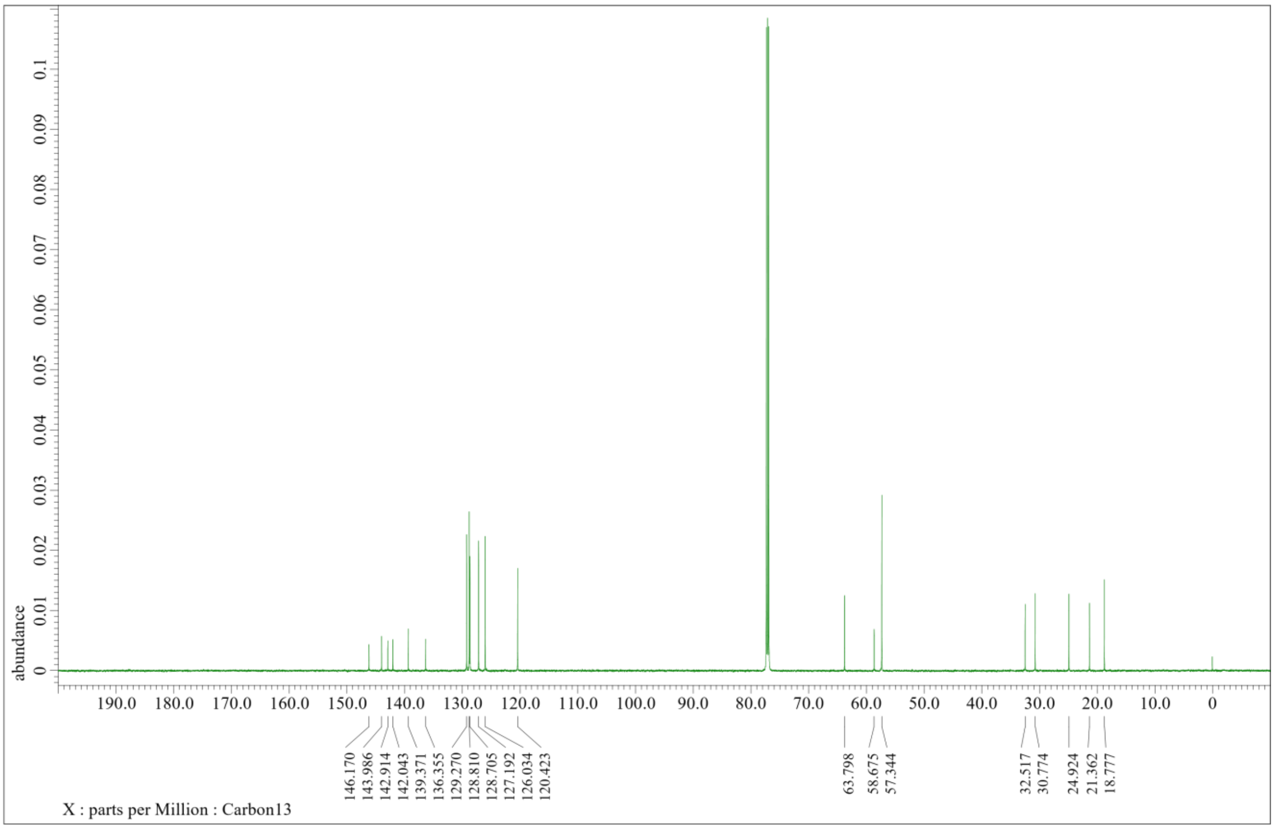

500 MHz, CDCl3

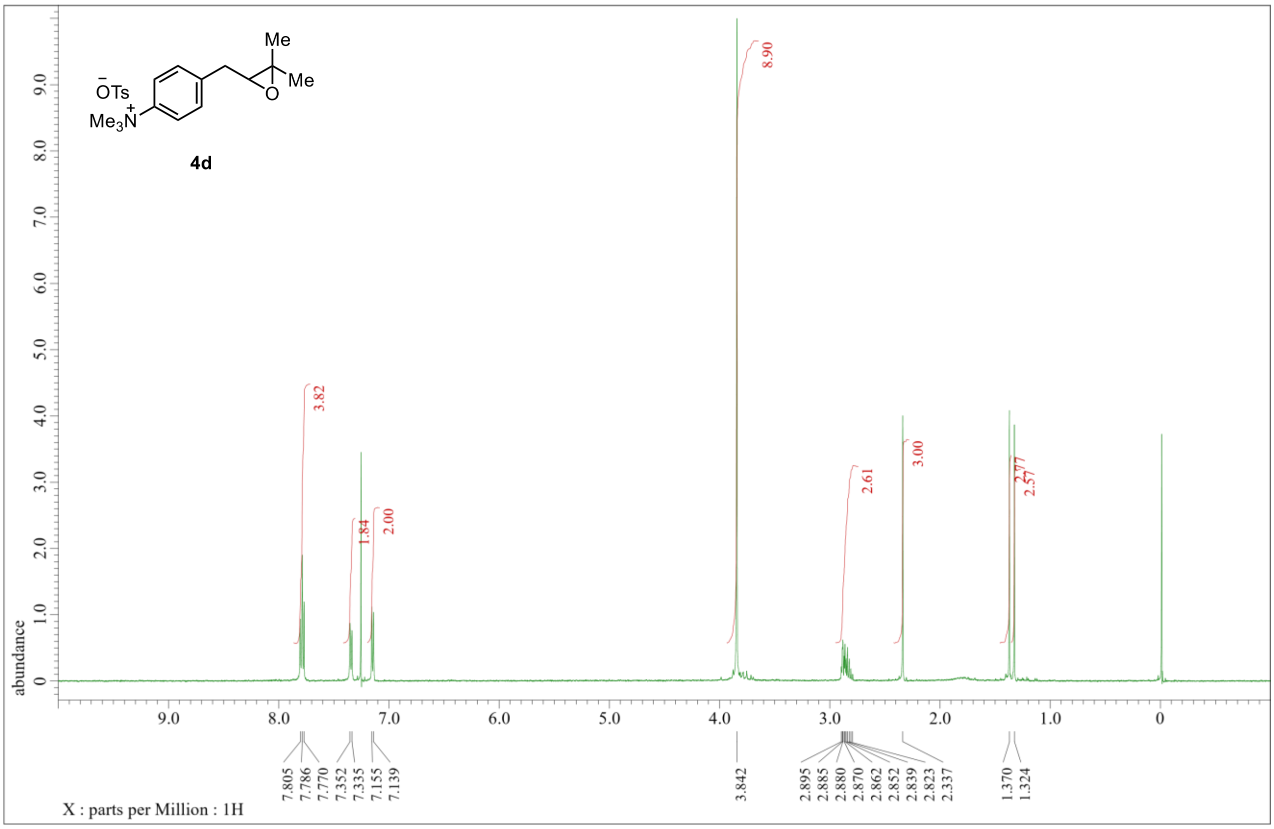

151 MHz, CDCl3

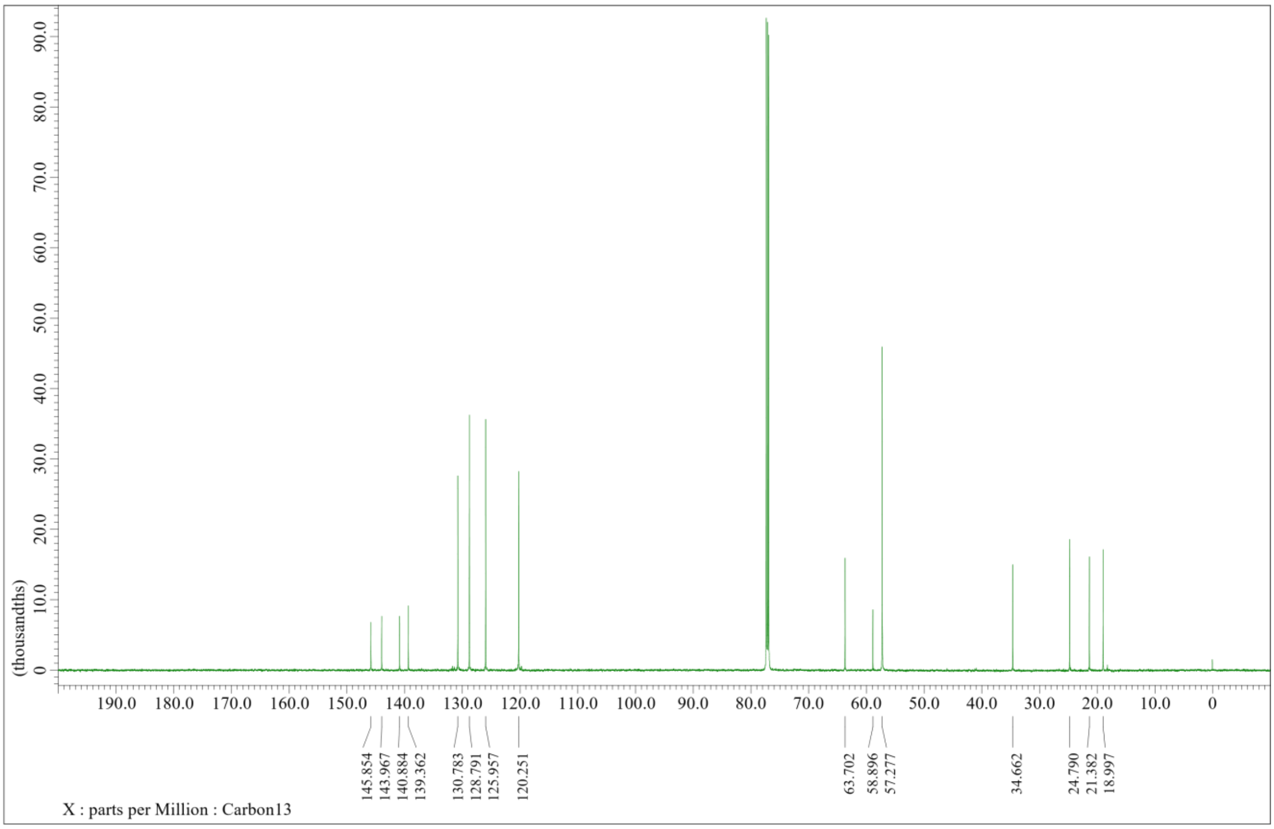

500 MHz, $CDCl_3$

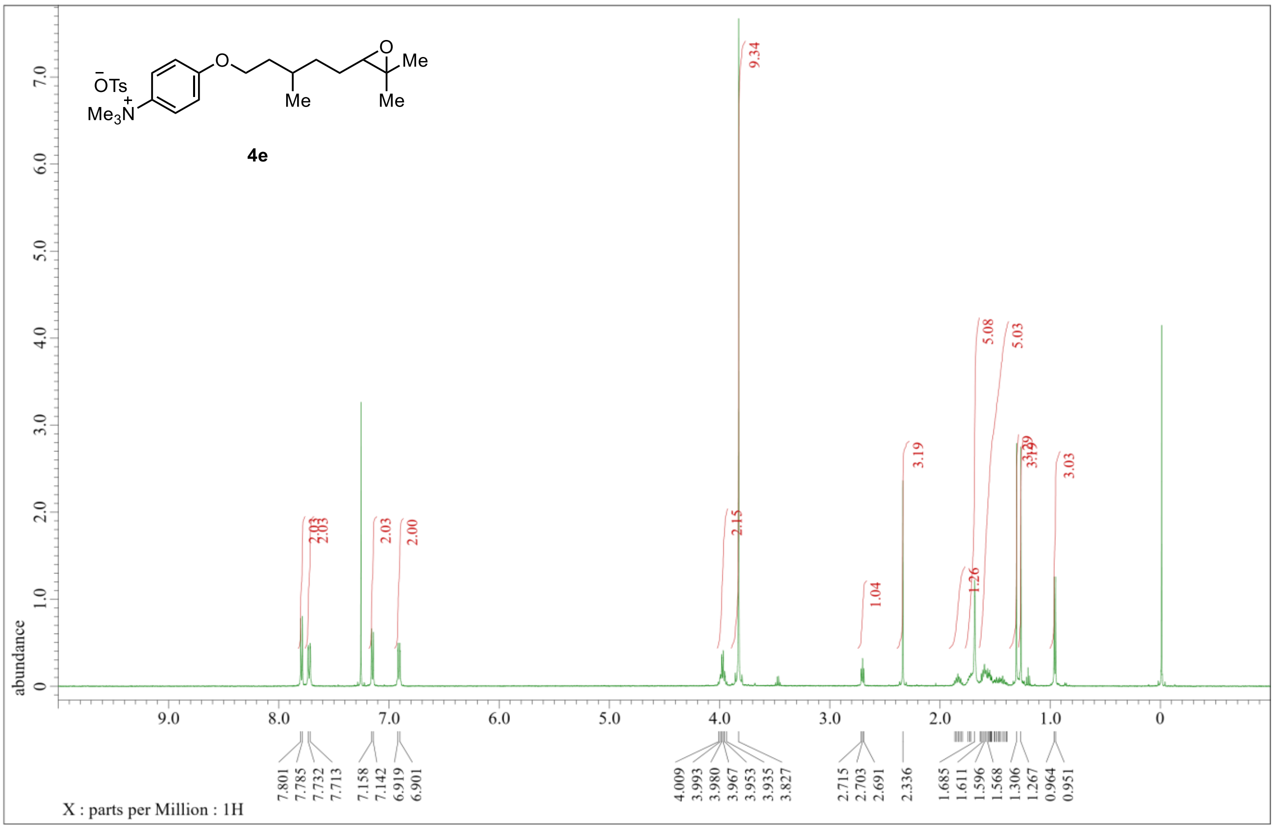

151 MHz, $CDCl_3$

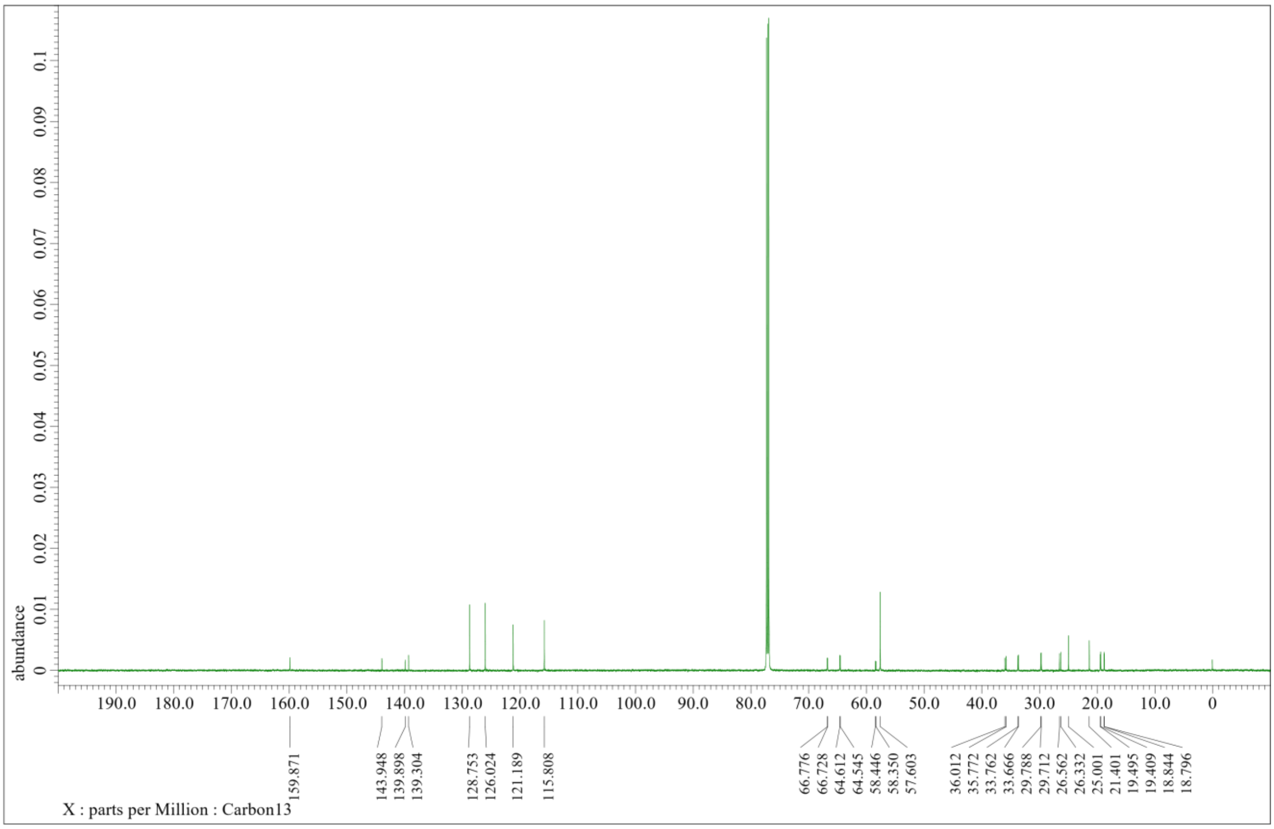

600 MHz, $CDCl_3$

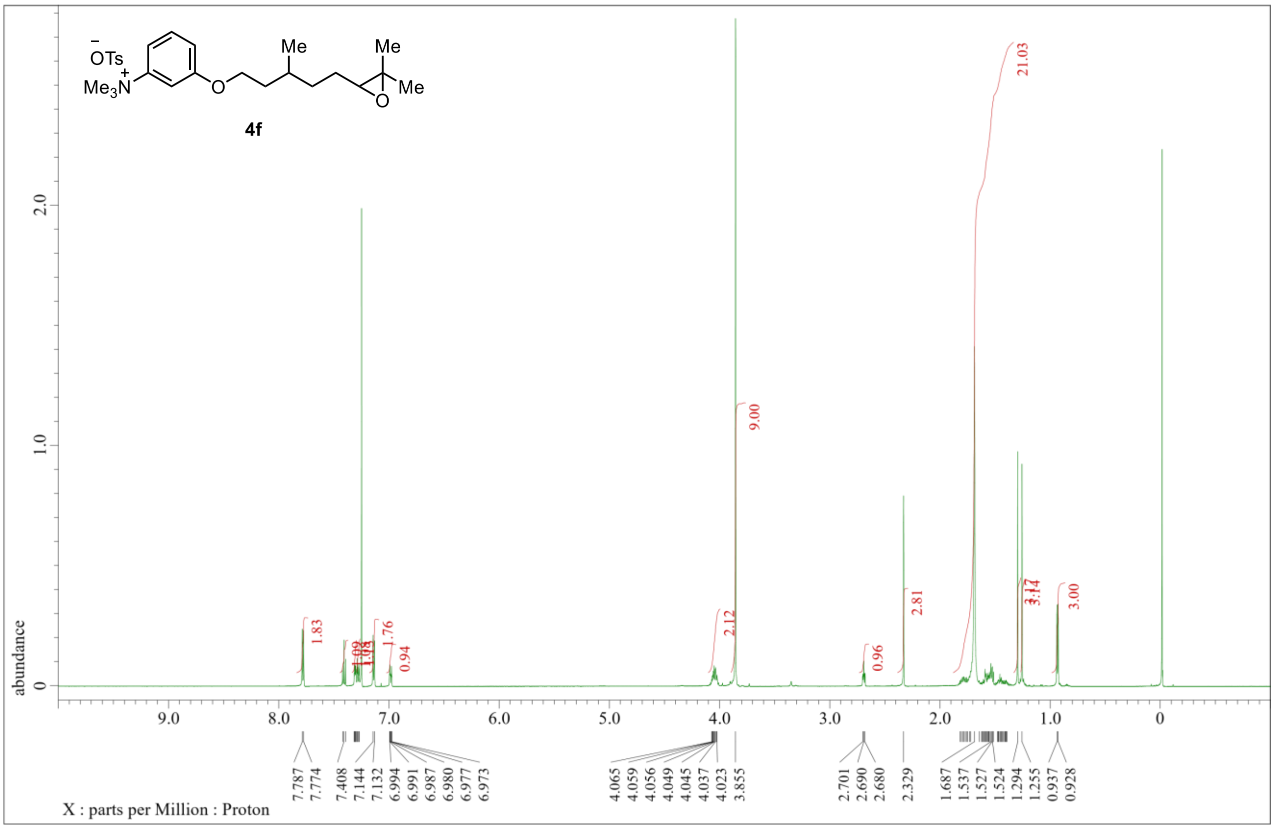

151 MHz, $CDCl_3$

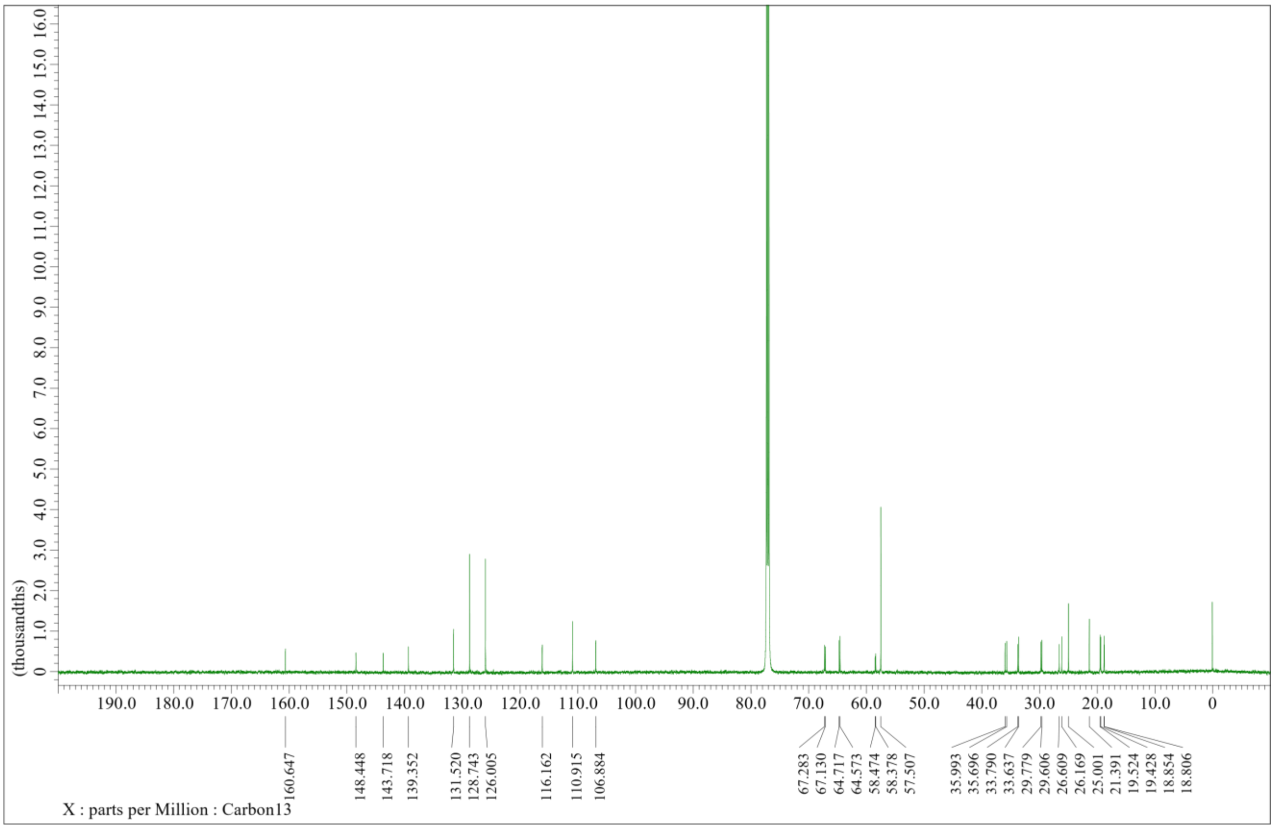

600 MHz, $CDCl_3$

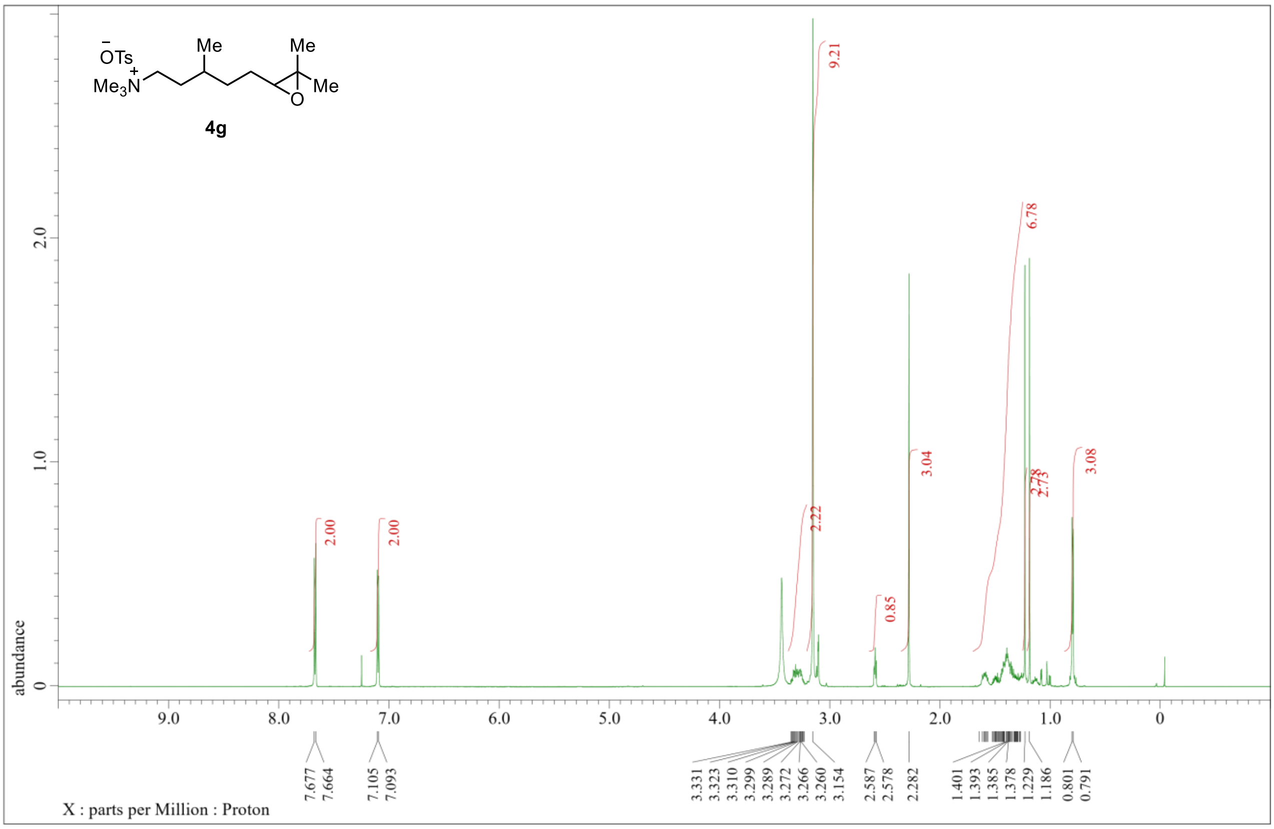

151 MHz, $CDCl_3$

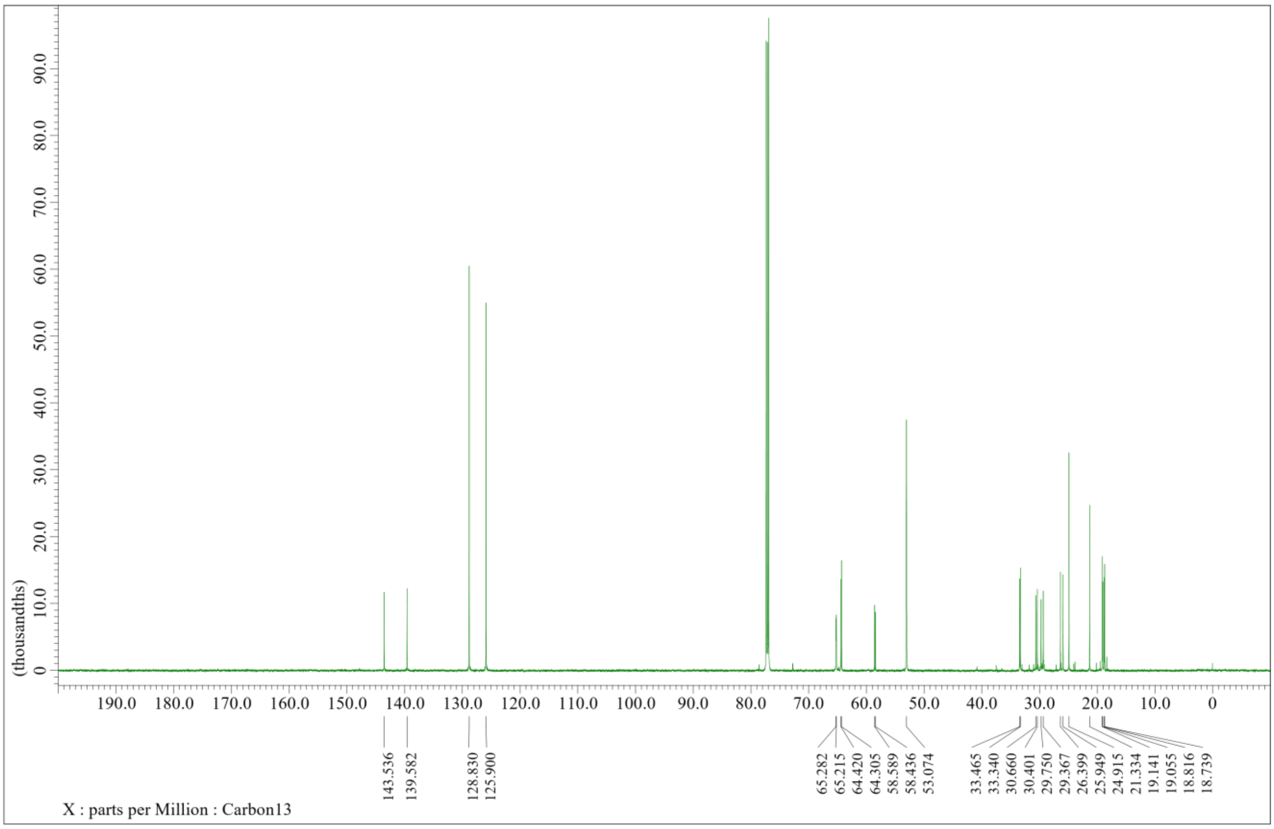

500 MHz, CDCl$_3$

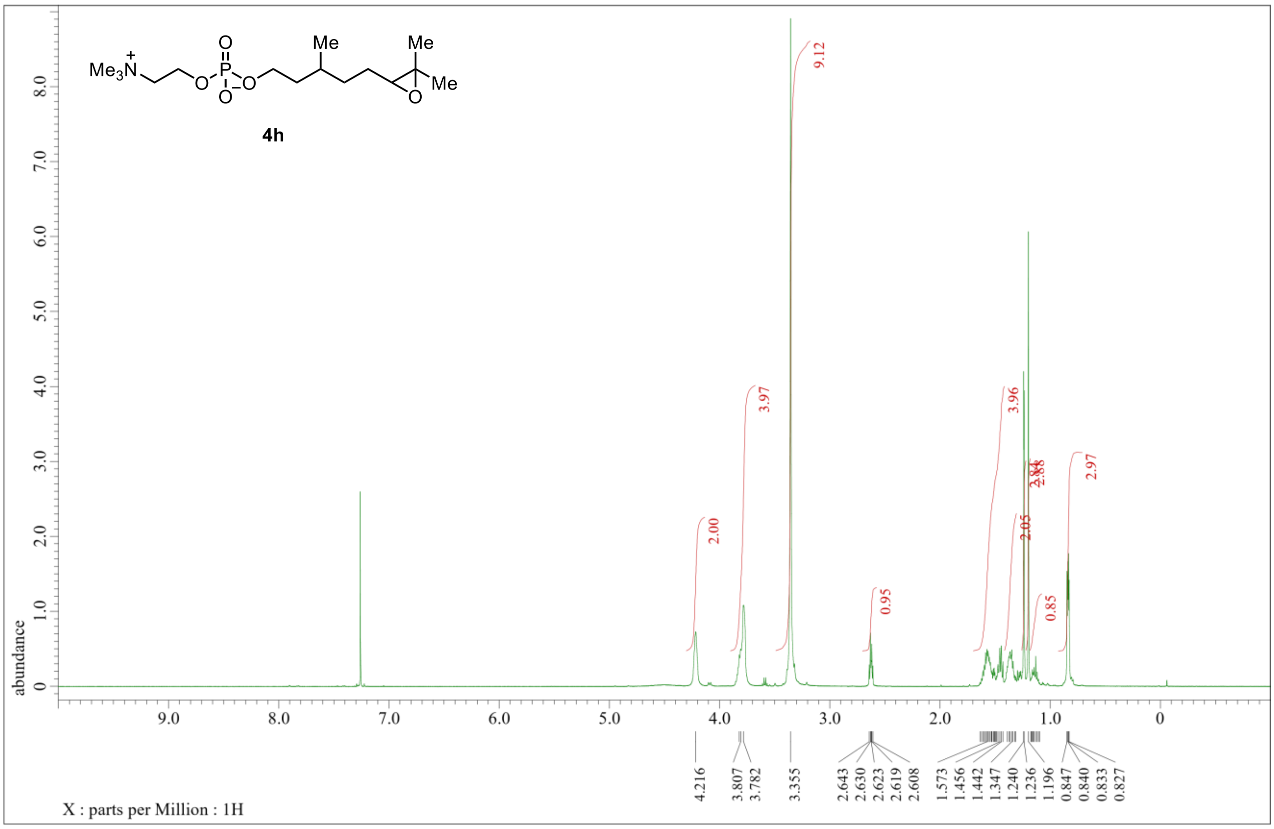

151 MHz, CDCl$_3$

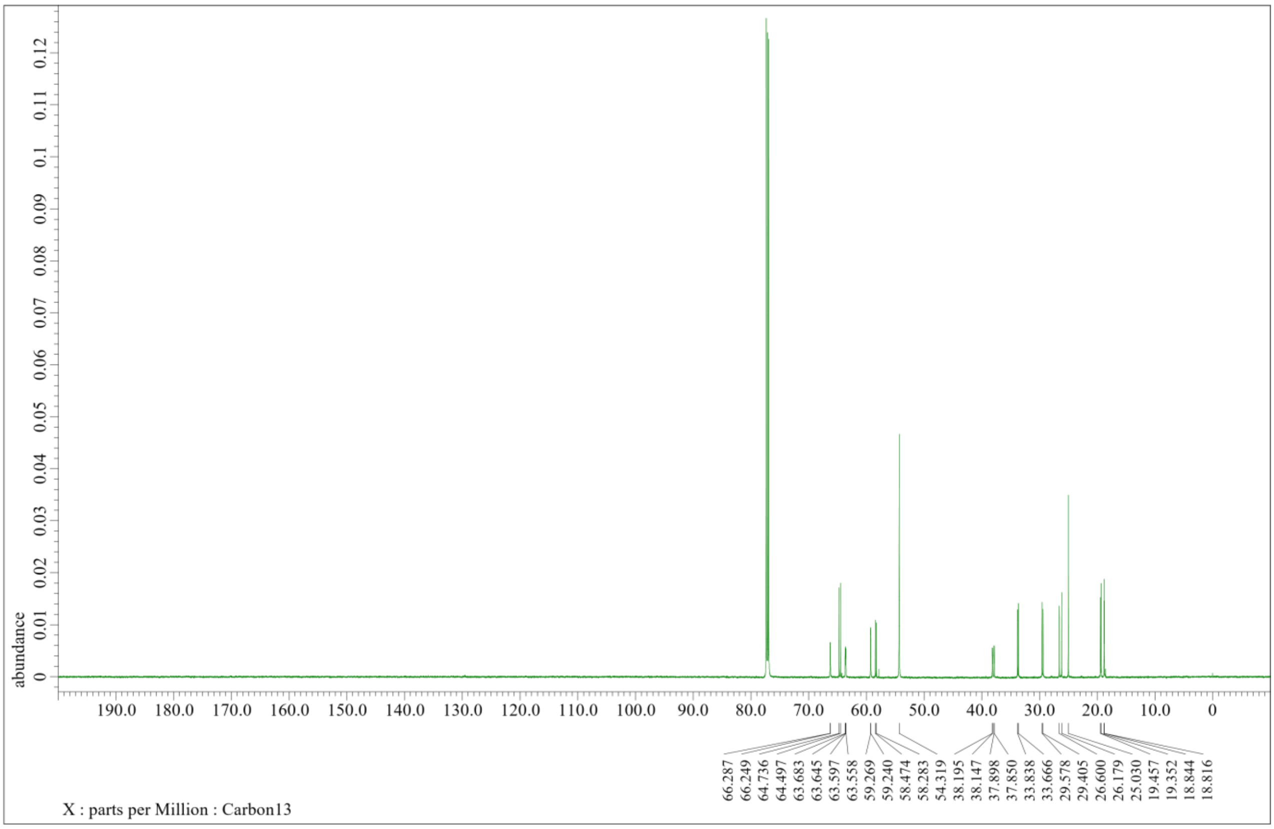

^{31}P NMR: 243 MHz, $CDCl_3$

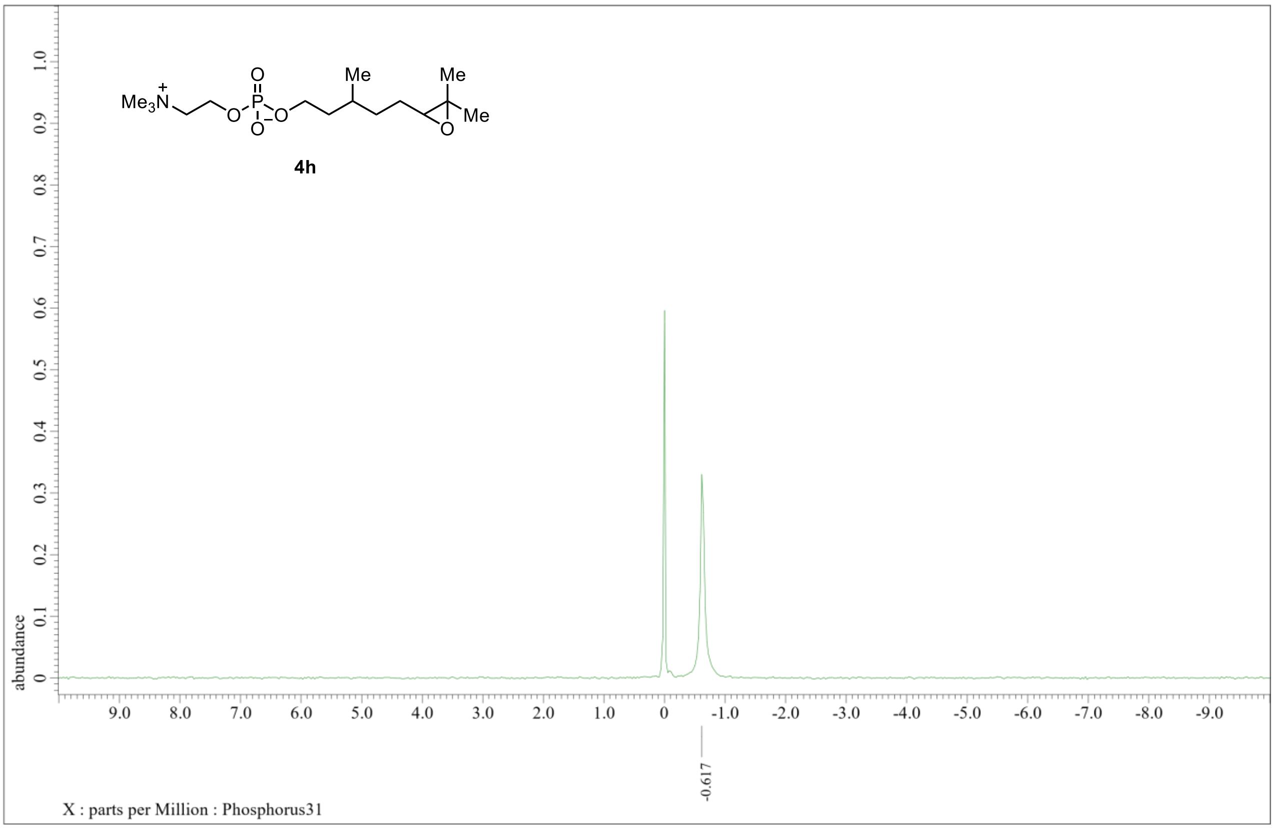

500 MHz, $CDCl_3$

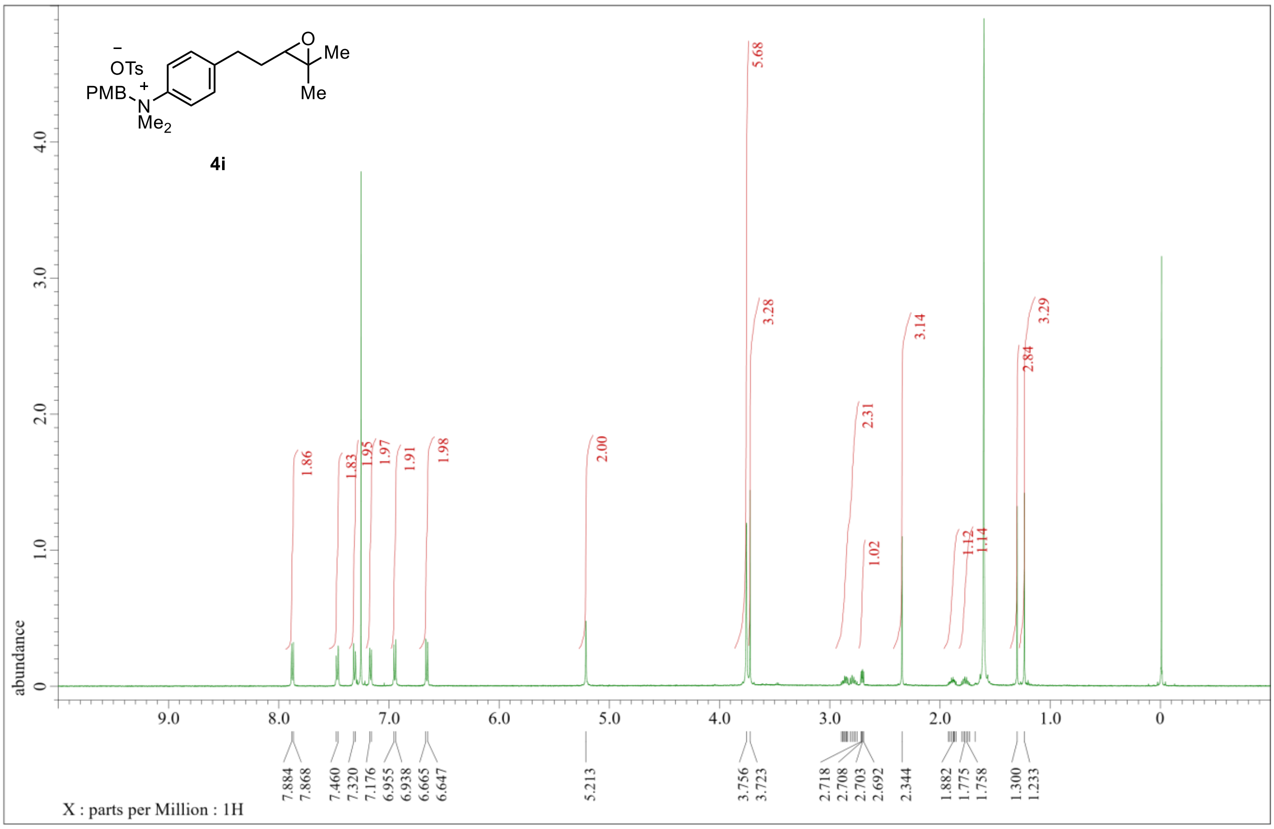

151 MHz, $CDCl_3$

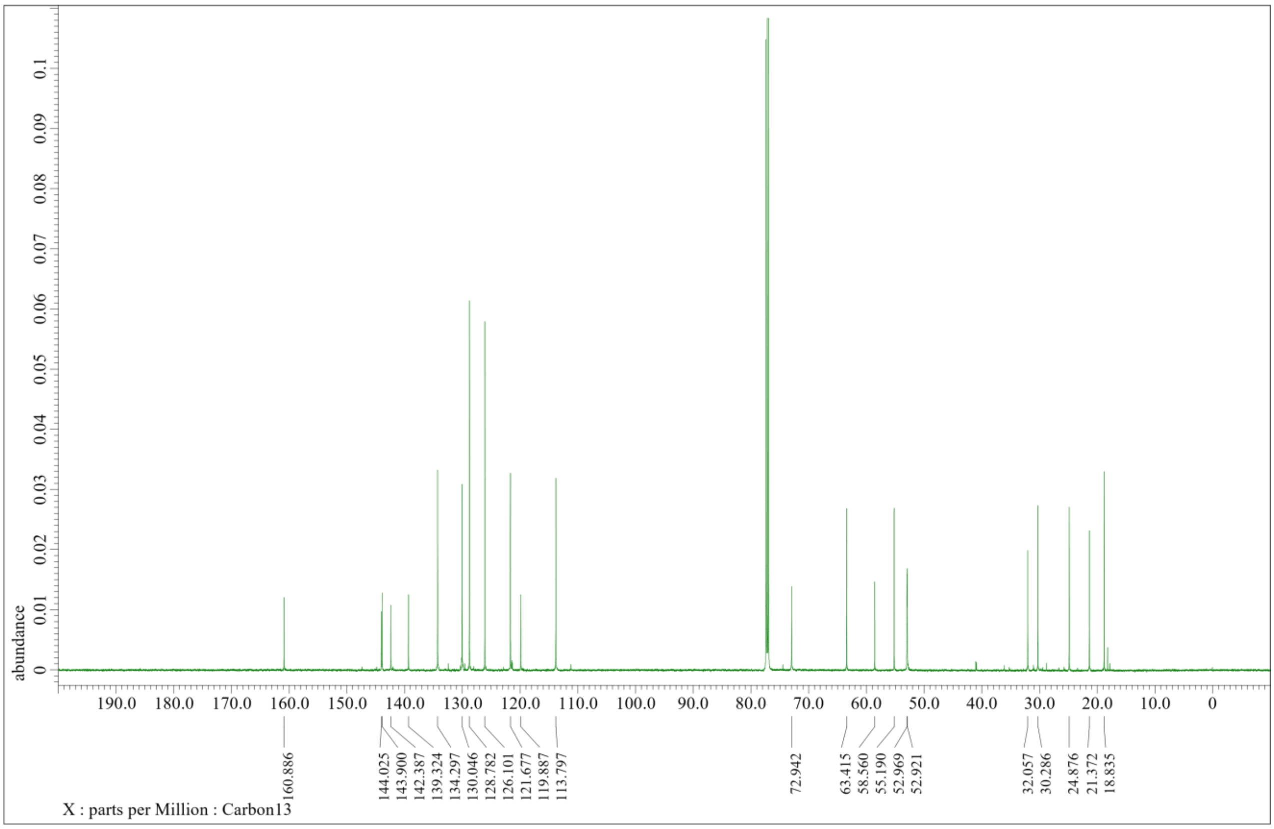

500 MHz, $CDCl_3$

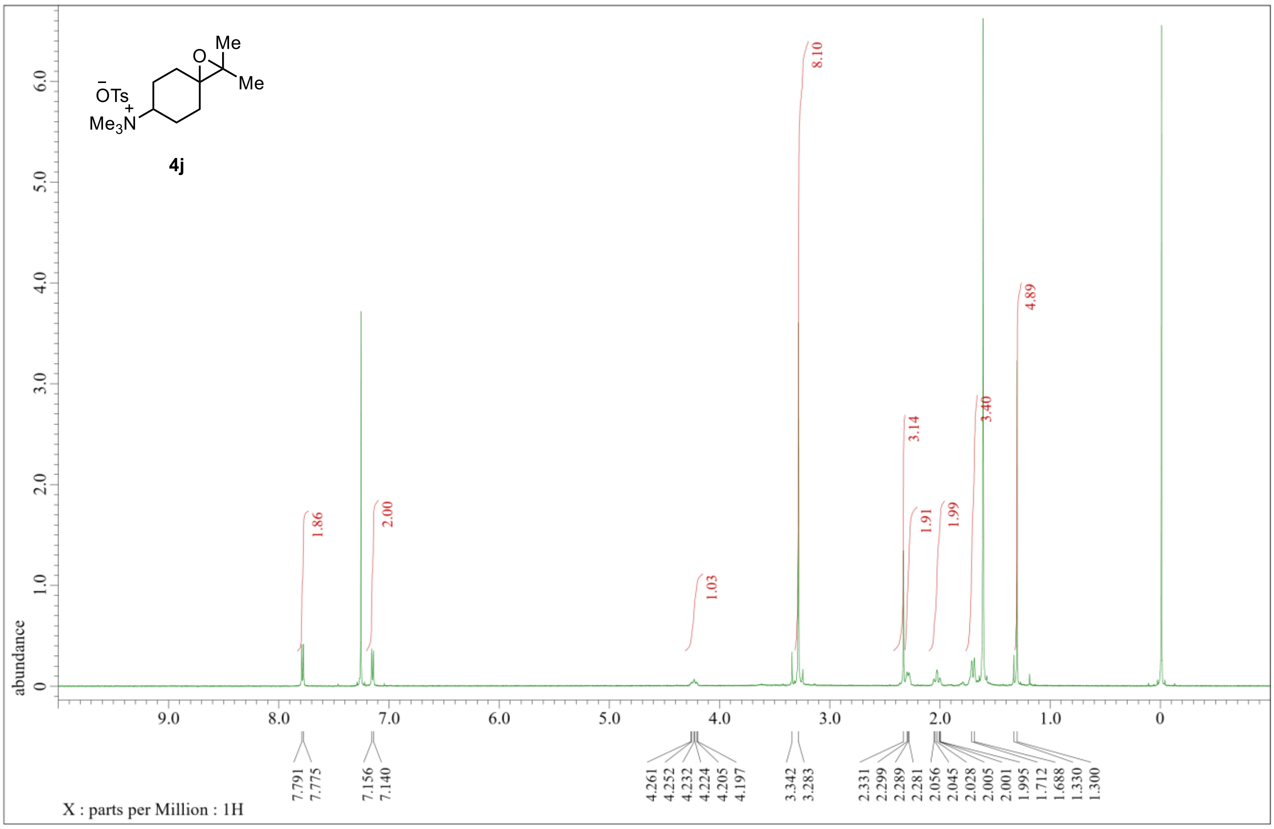

151 MHz, $CDCl_3$

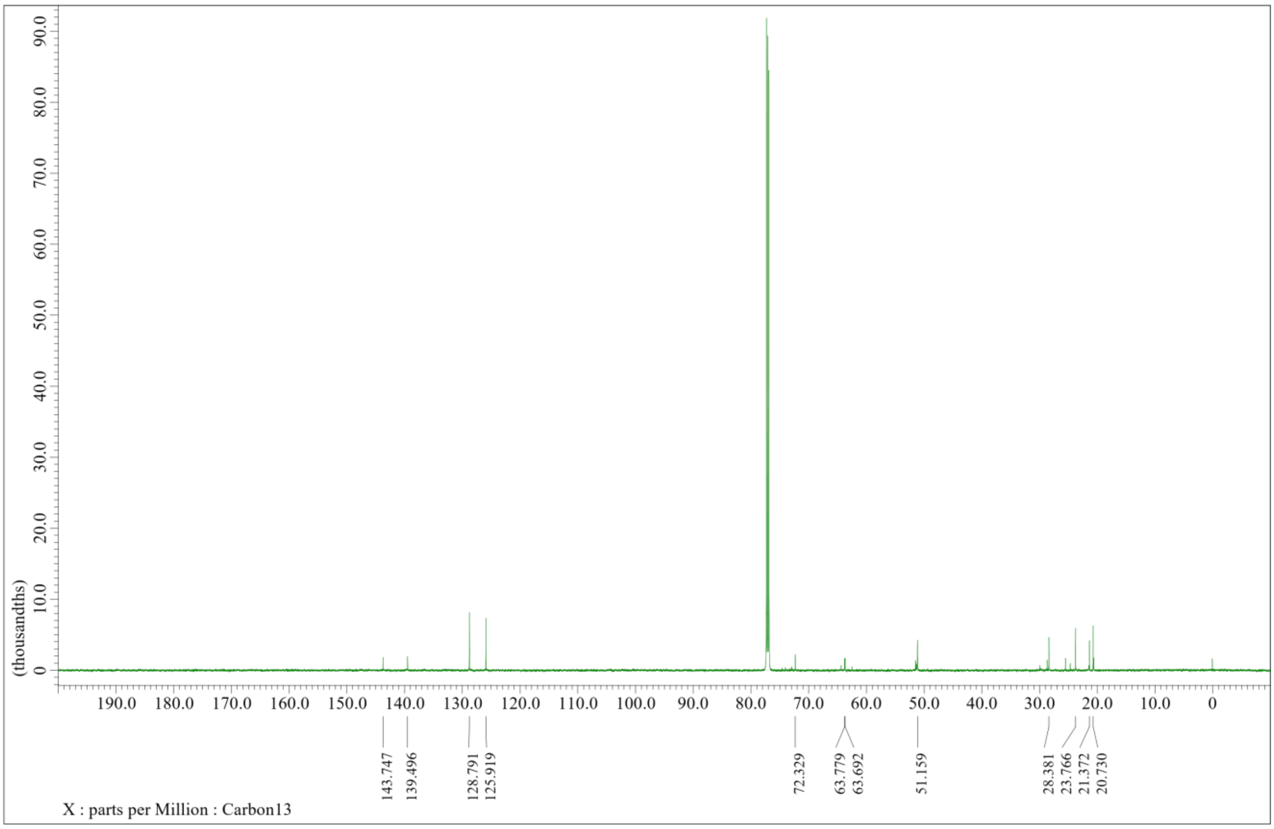

600 MHz, CDCl3

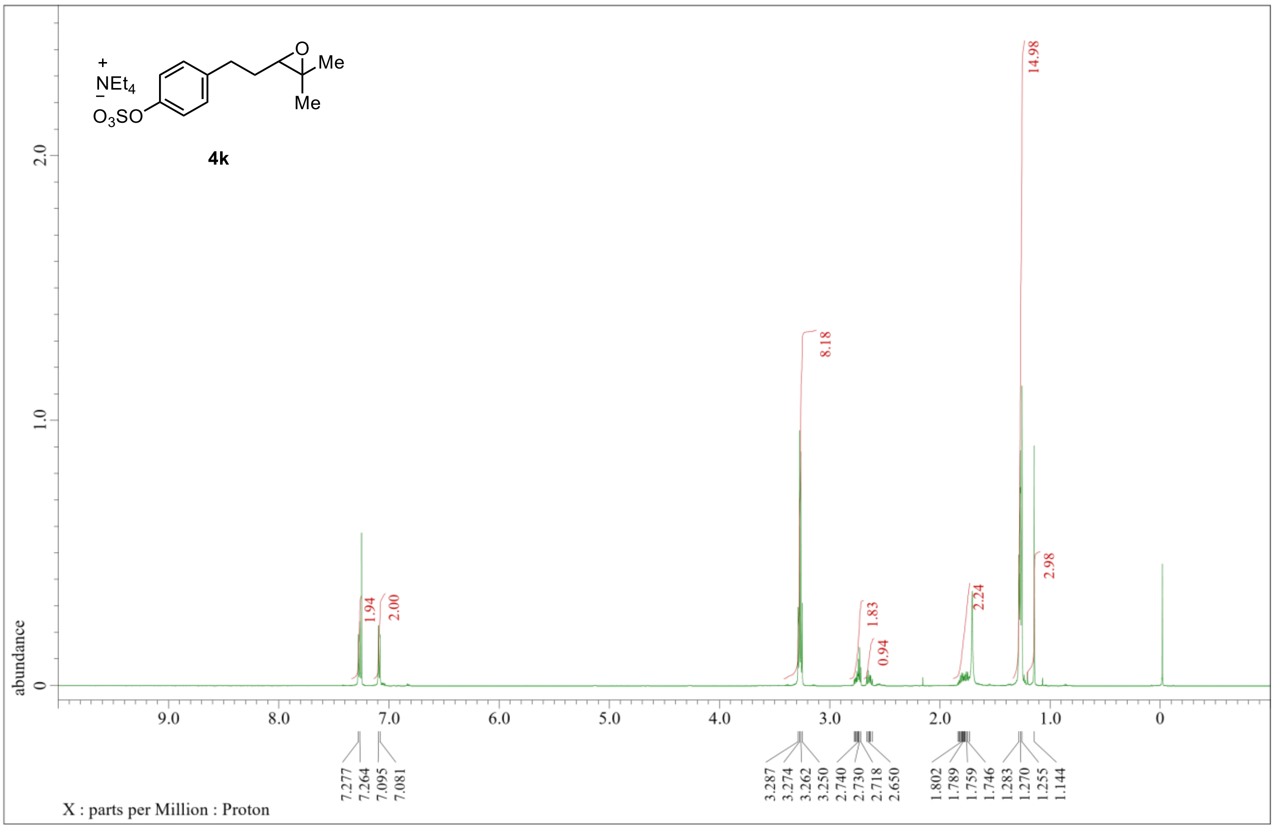

151 MHz, CDCl3

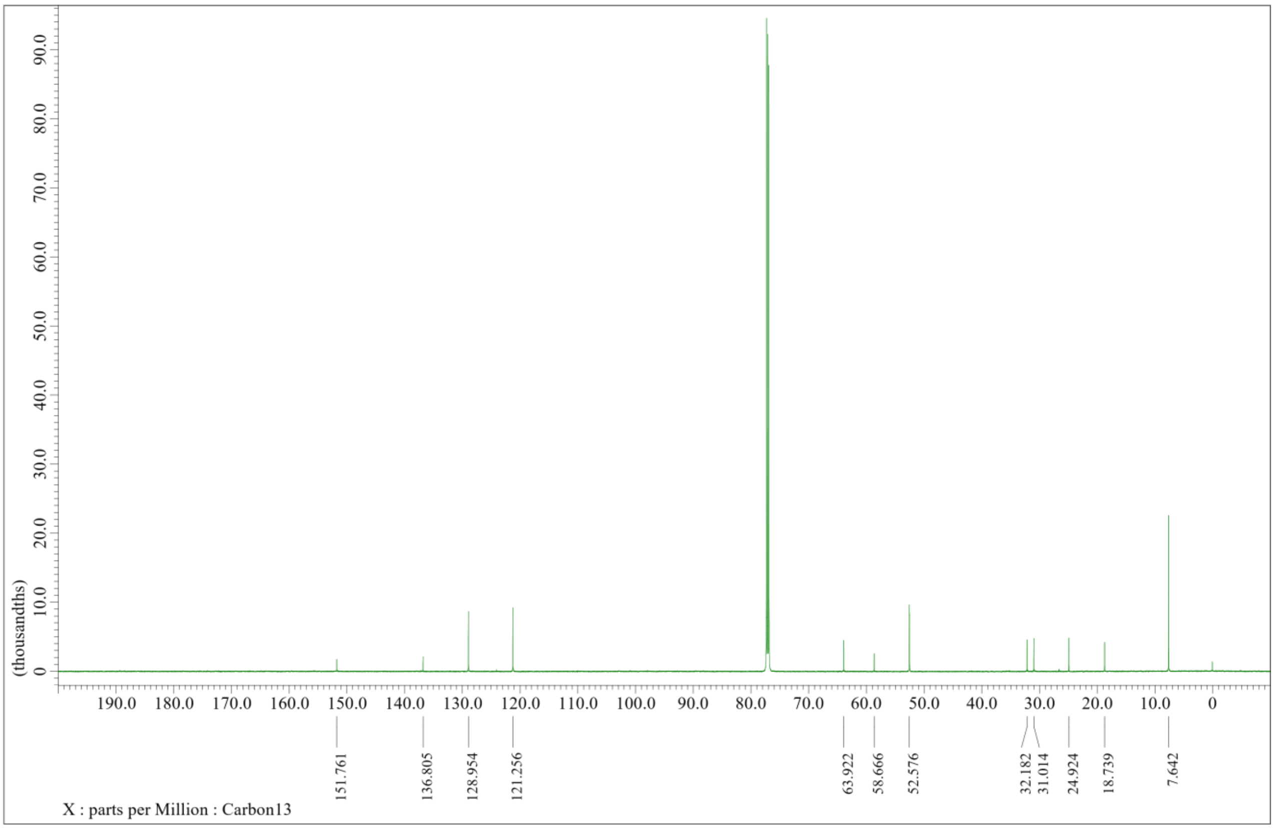

500 MHz, $CDCl_3$

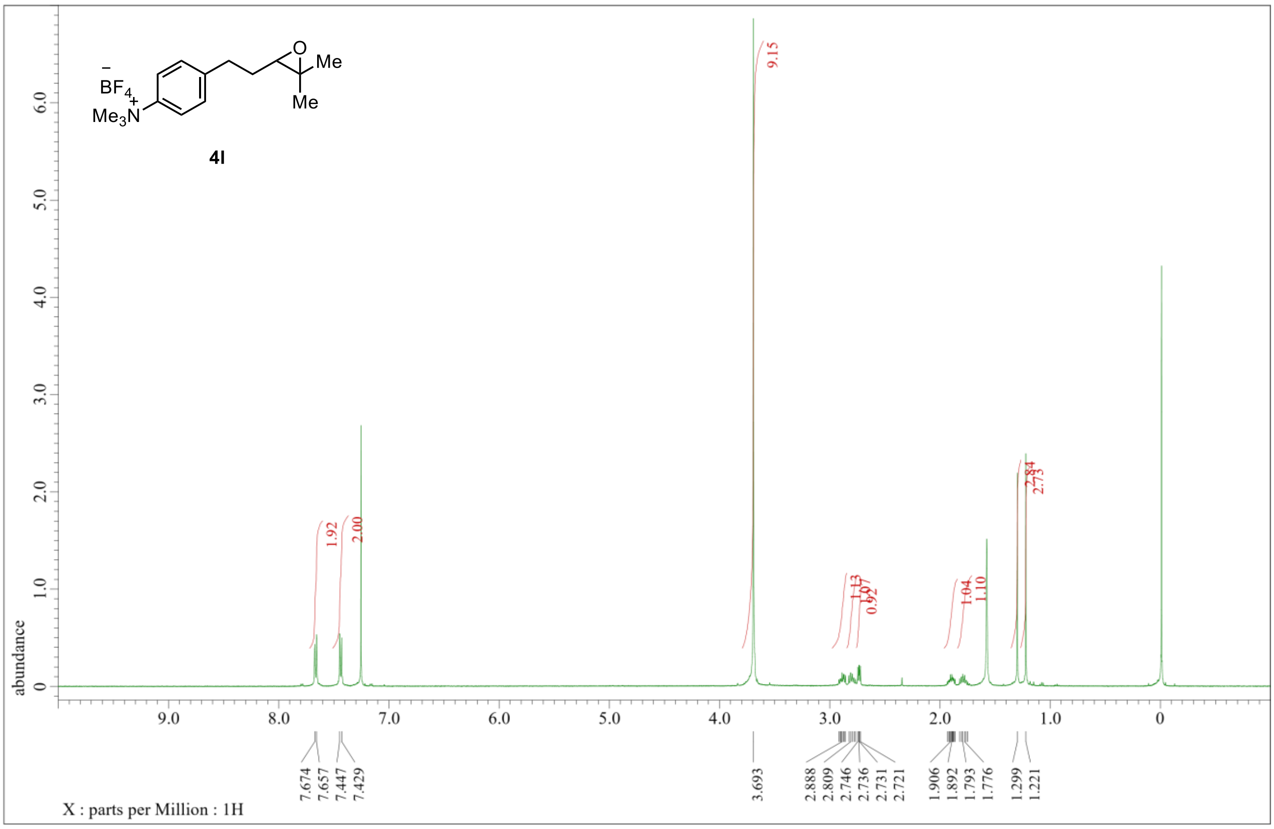

151 MHz, $CDCl_3$

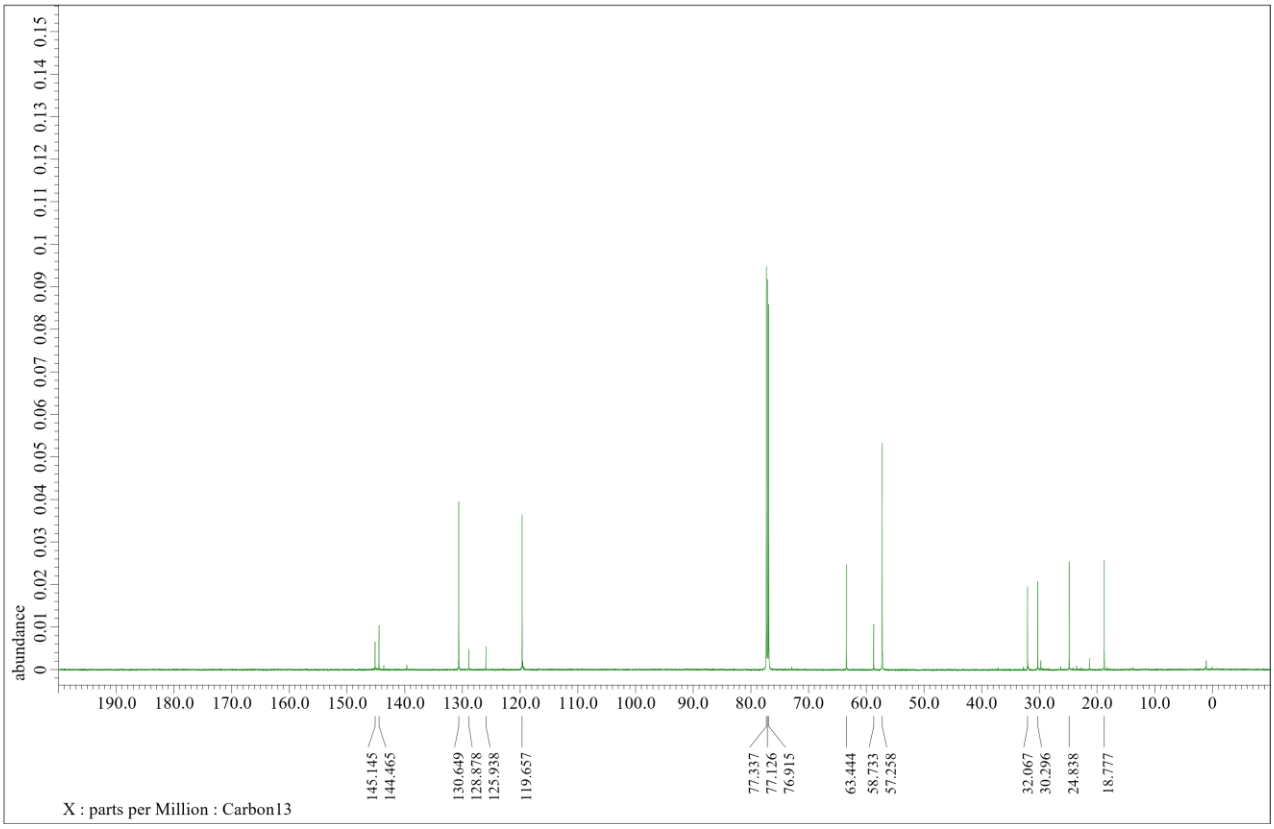

^{19}F NMR: 471 MHz, $CDCl_3$

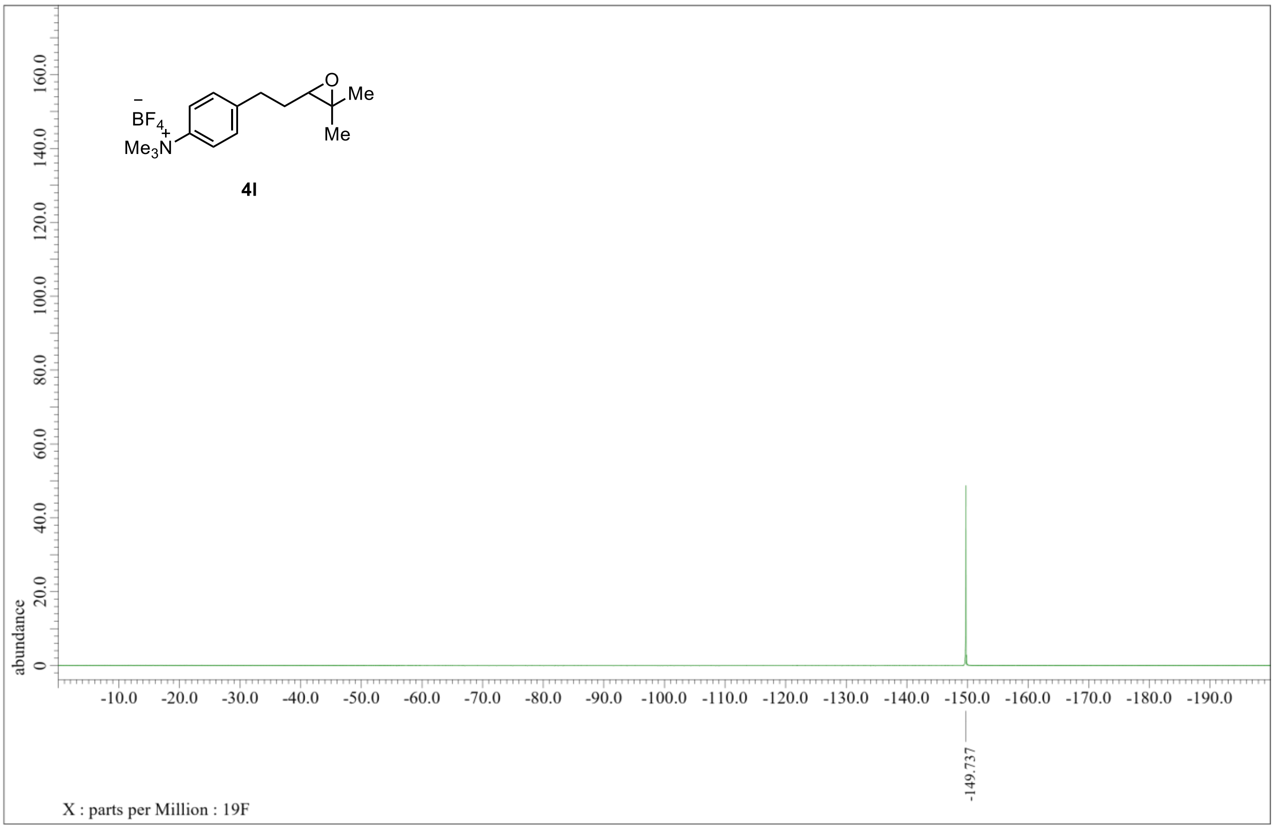

600 MHz, CD_3OD

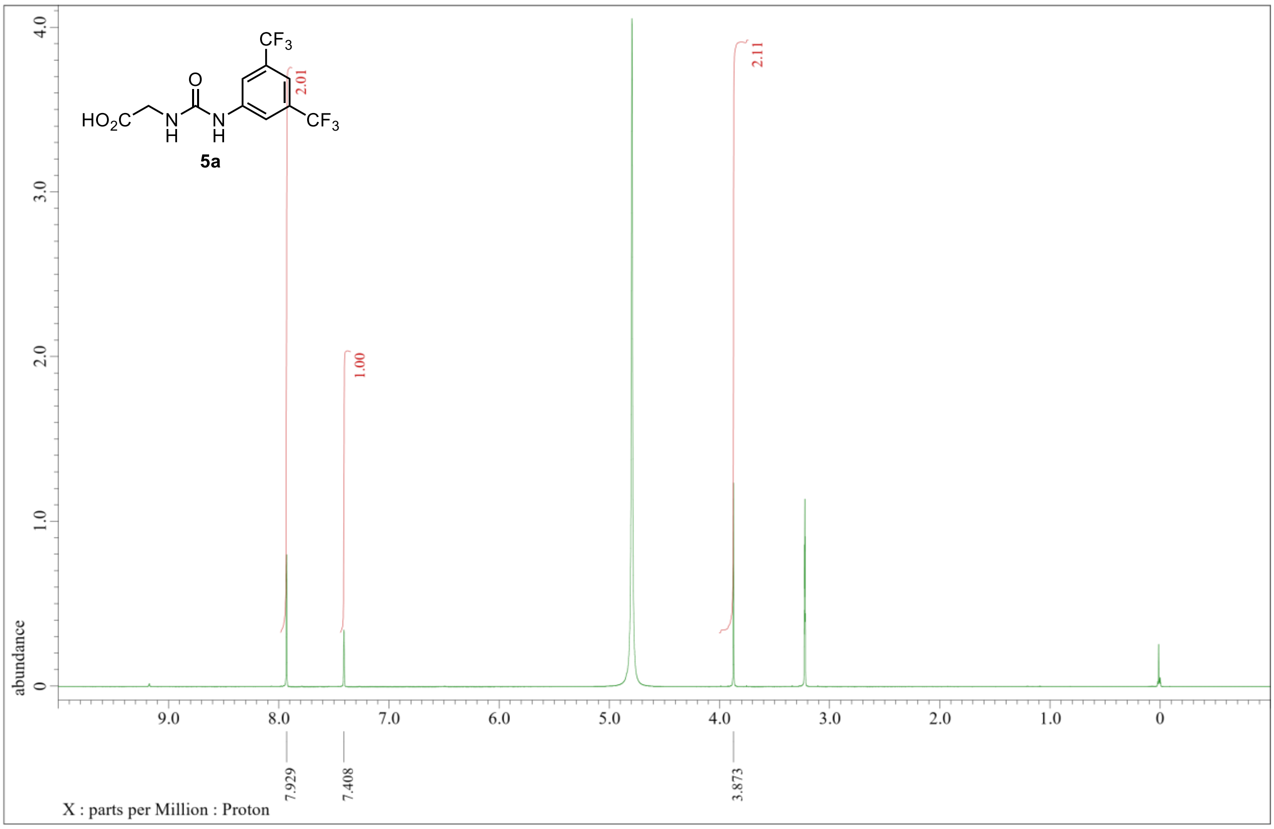

151 MHz, CD_3OD

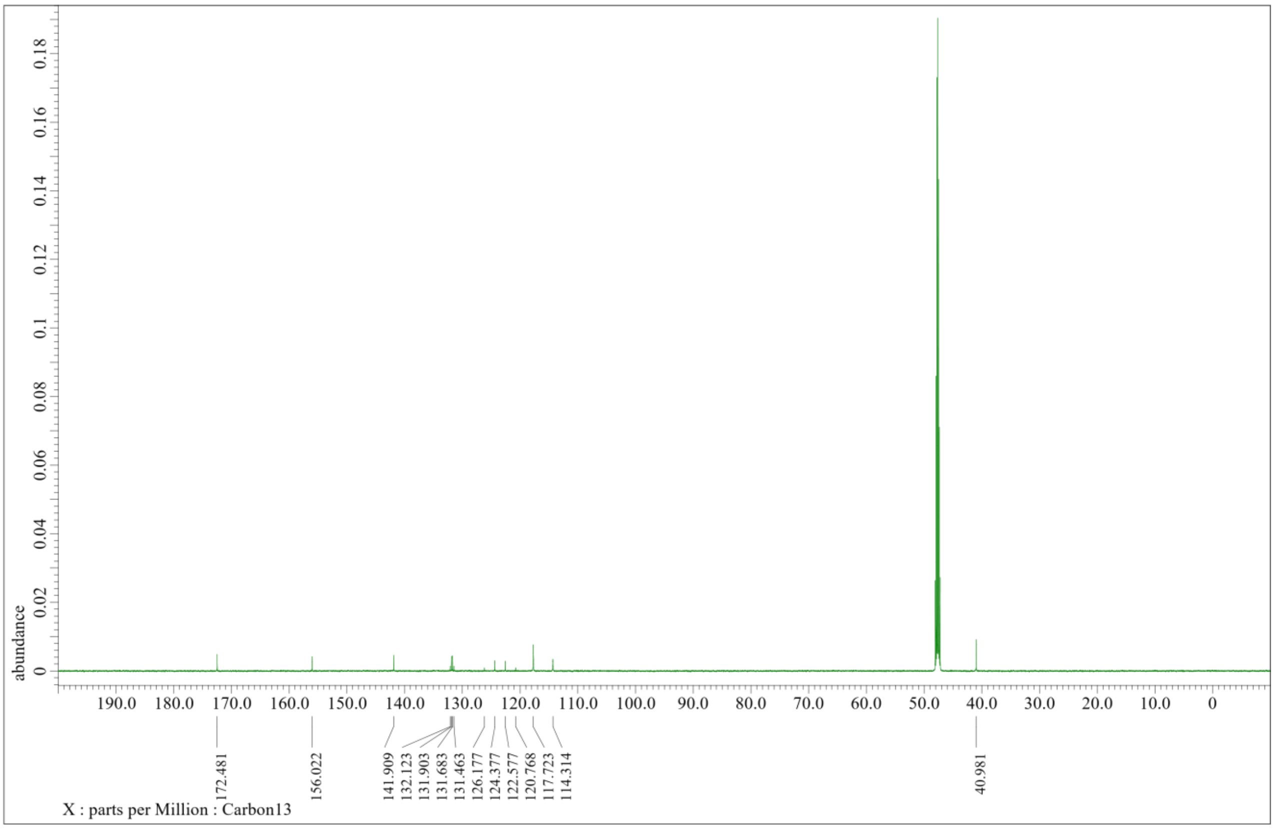

^{19}F NMR: 471 MHz, $CDCl_3$

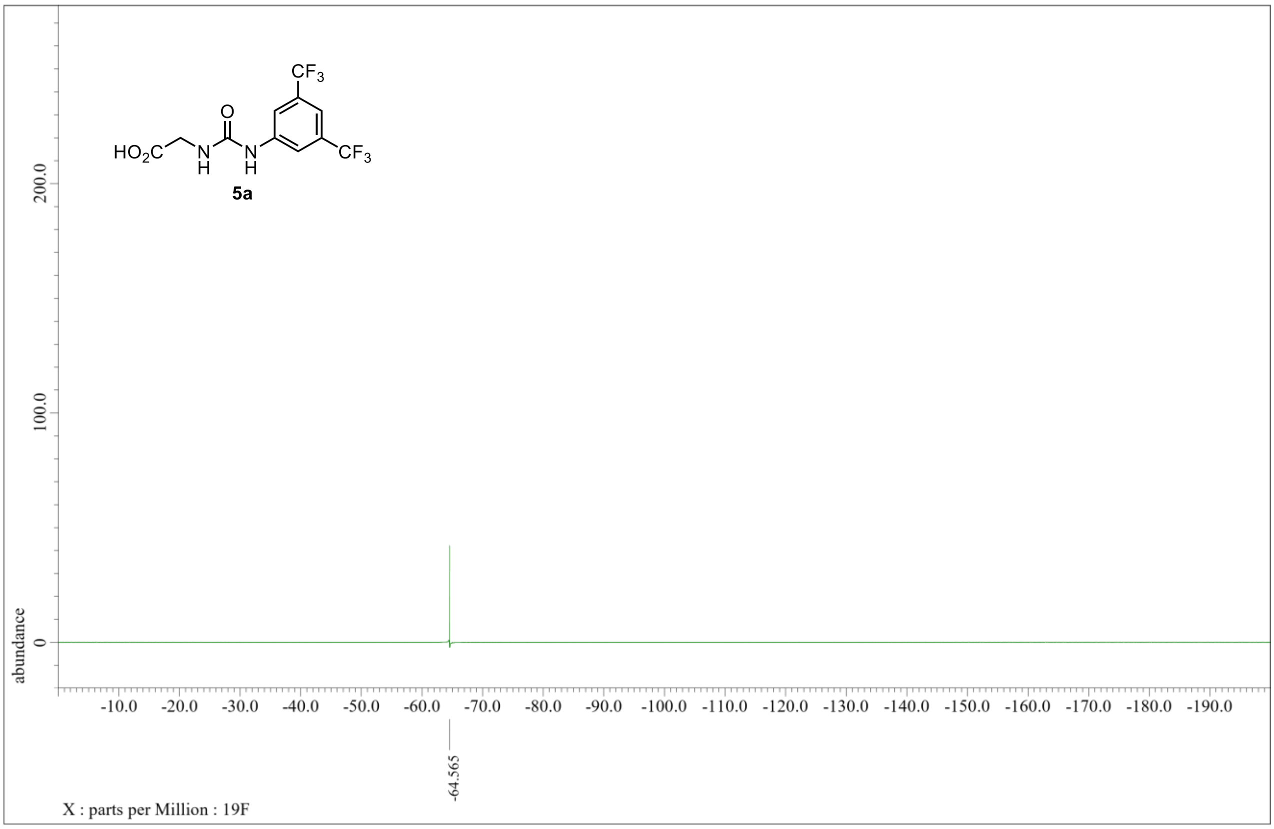

600 MHz, CD_3OD

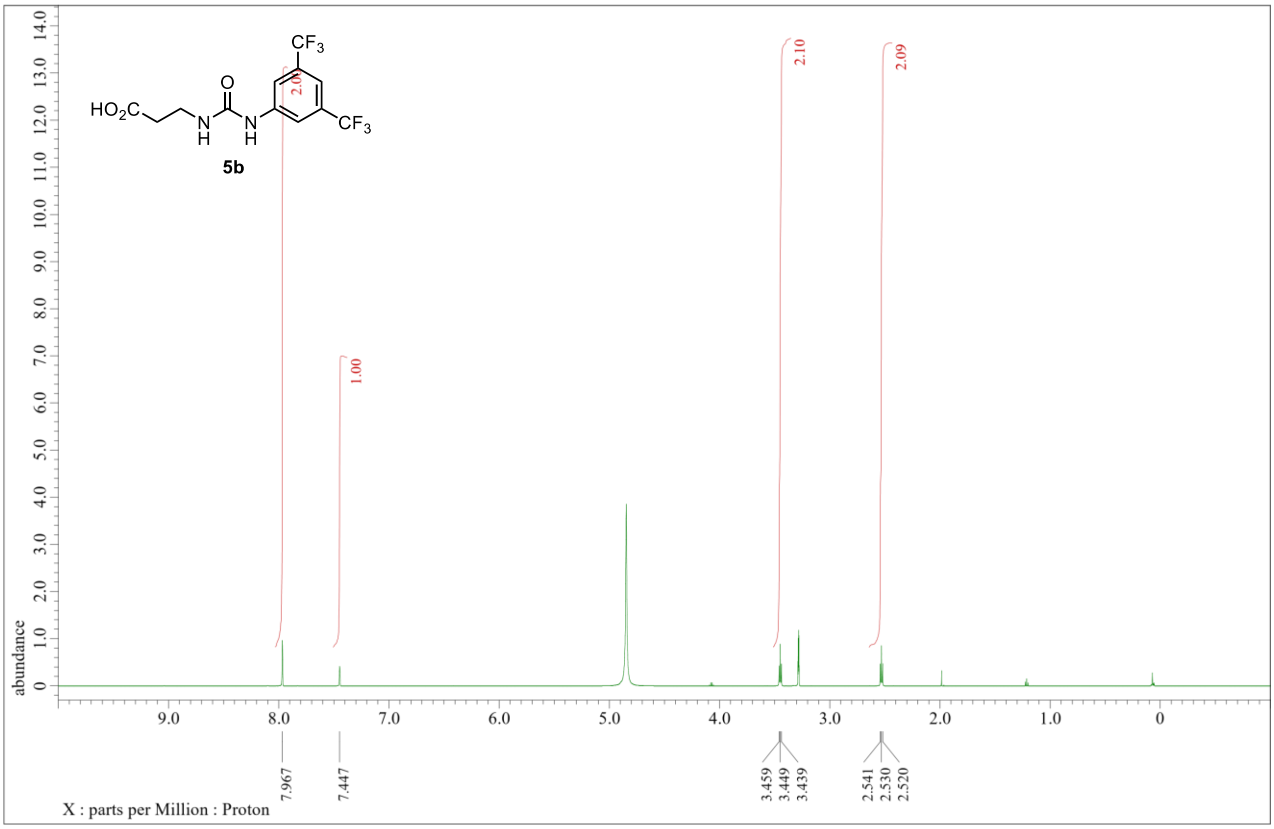

151 MHz, CD_3OD

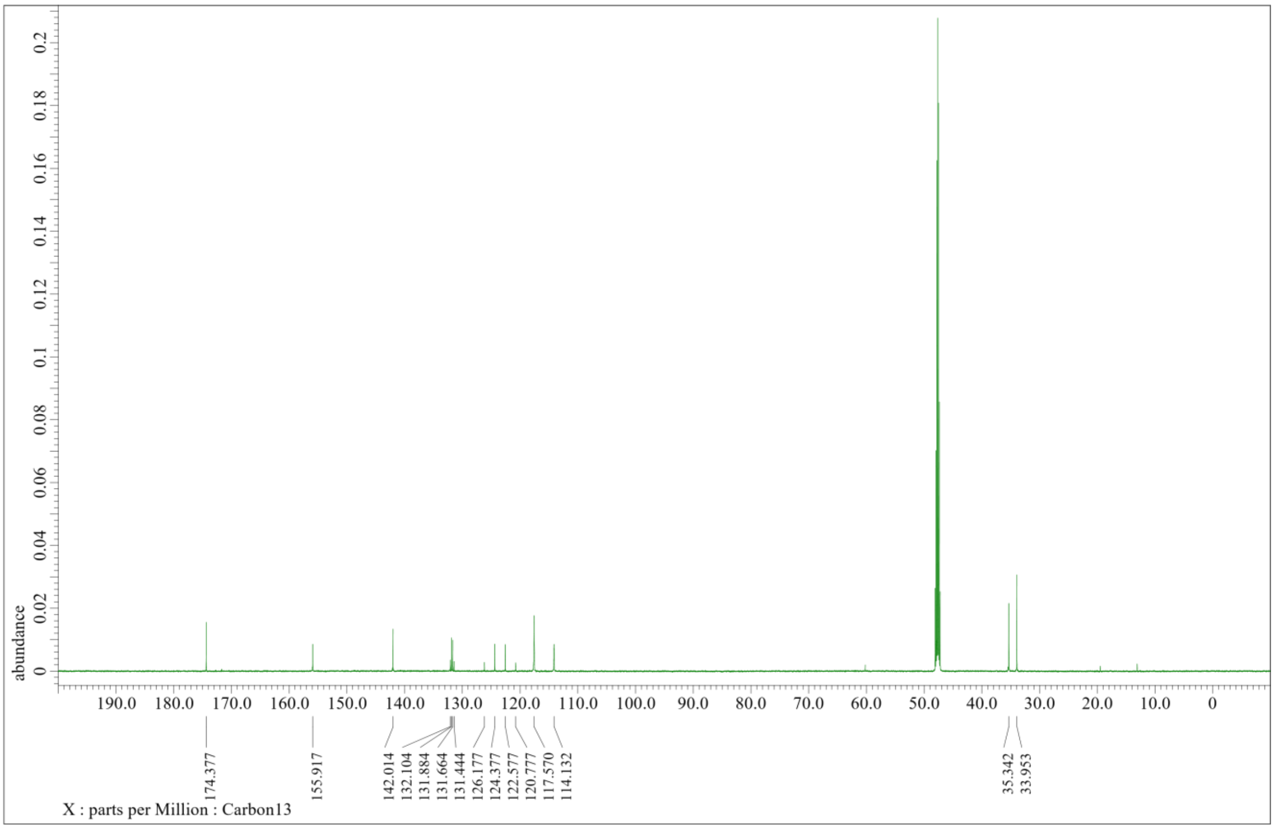

^{19}F NMR: 471 MHz, CD_3OD

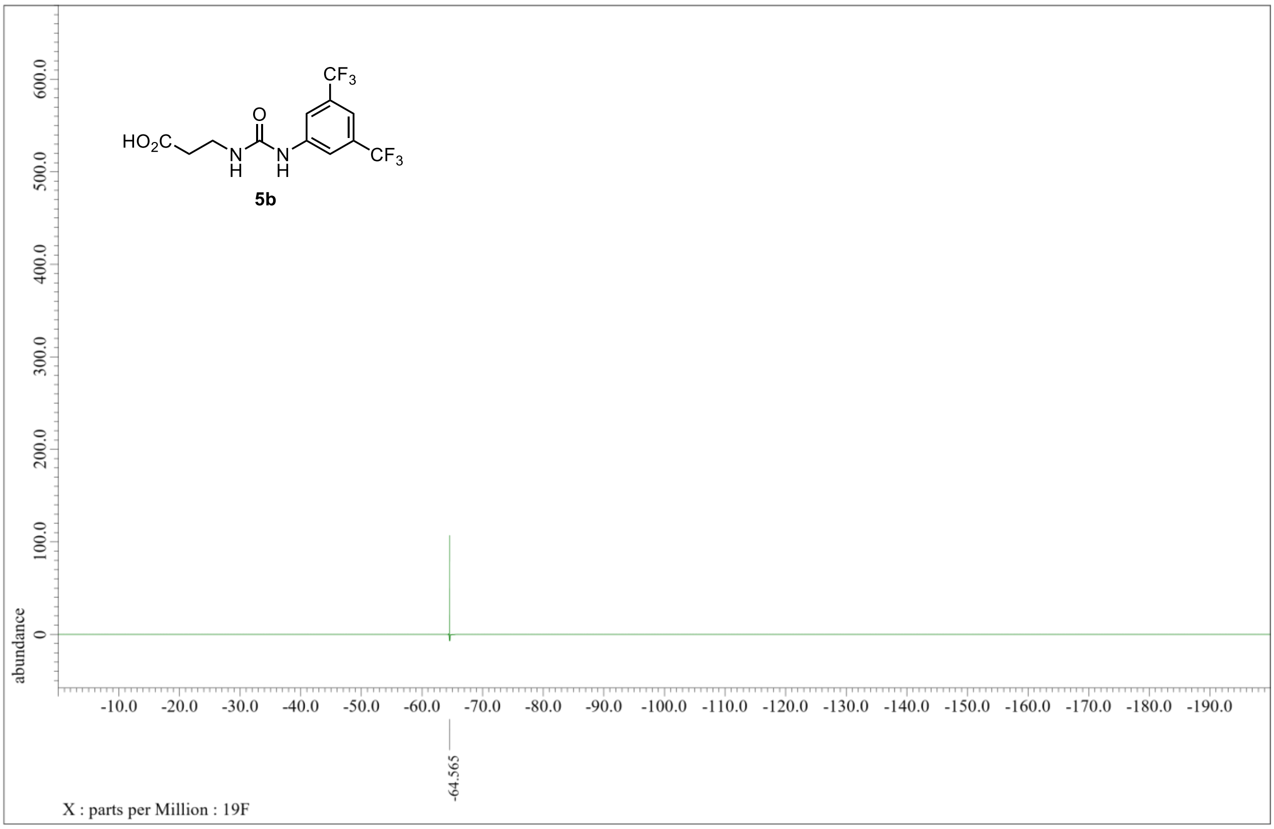

600 MHz, CD_3OD

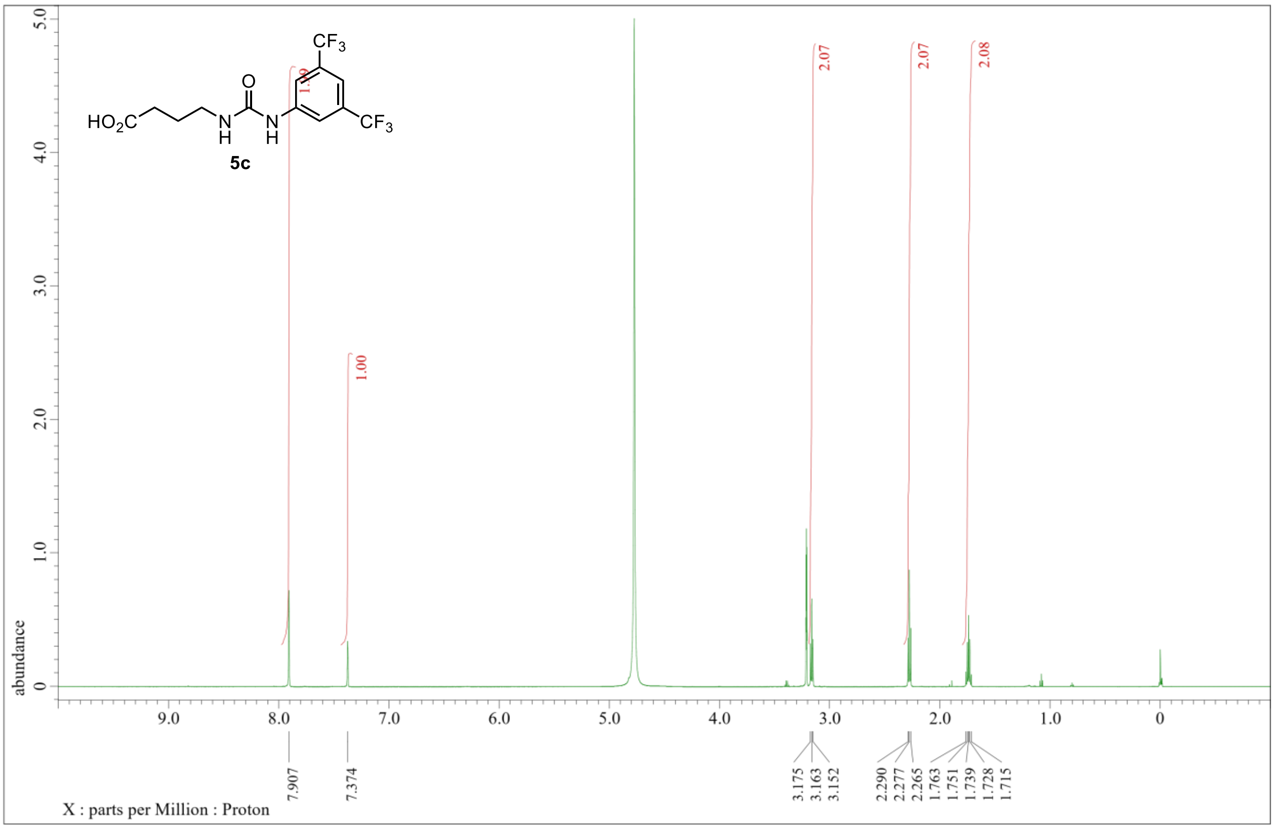

151 MHz, CD_3OD

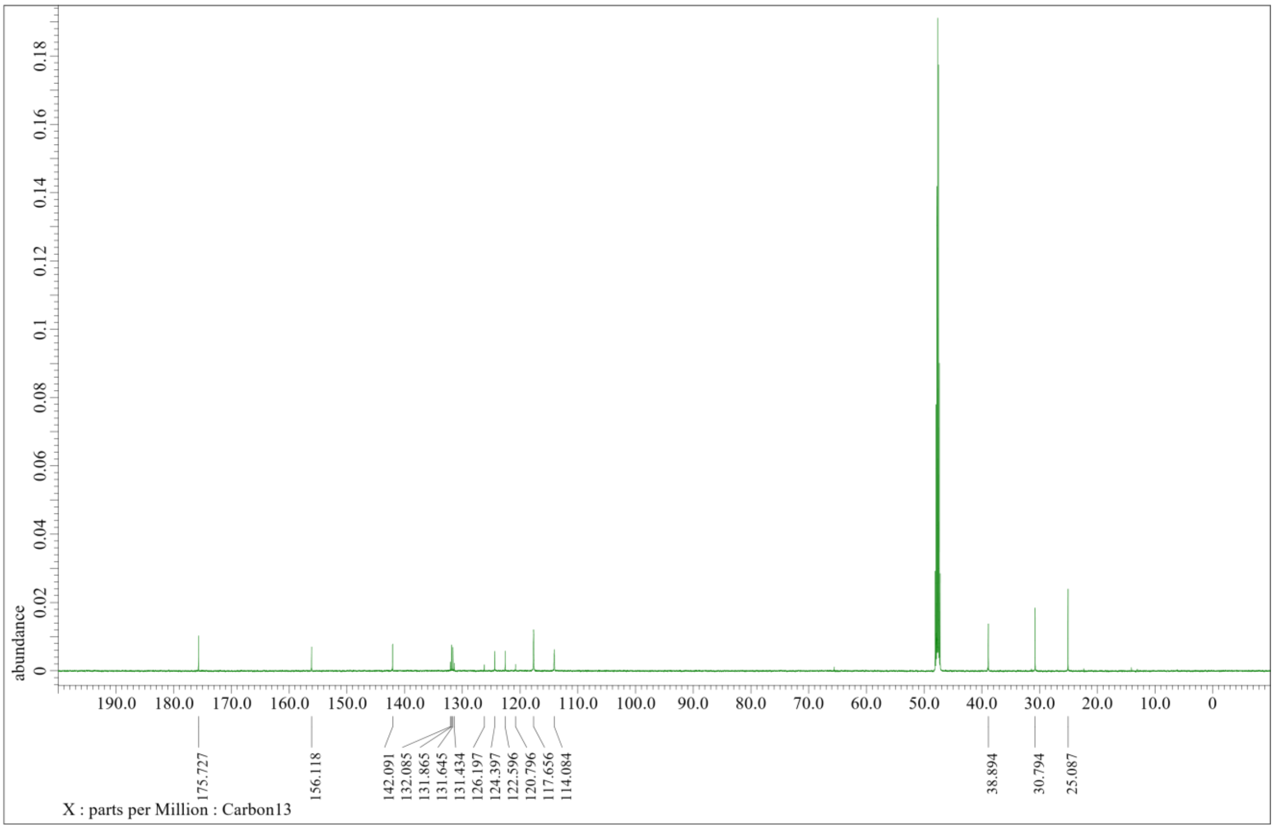

^{19}F NMR: 471 MHz, CD_3OD

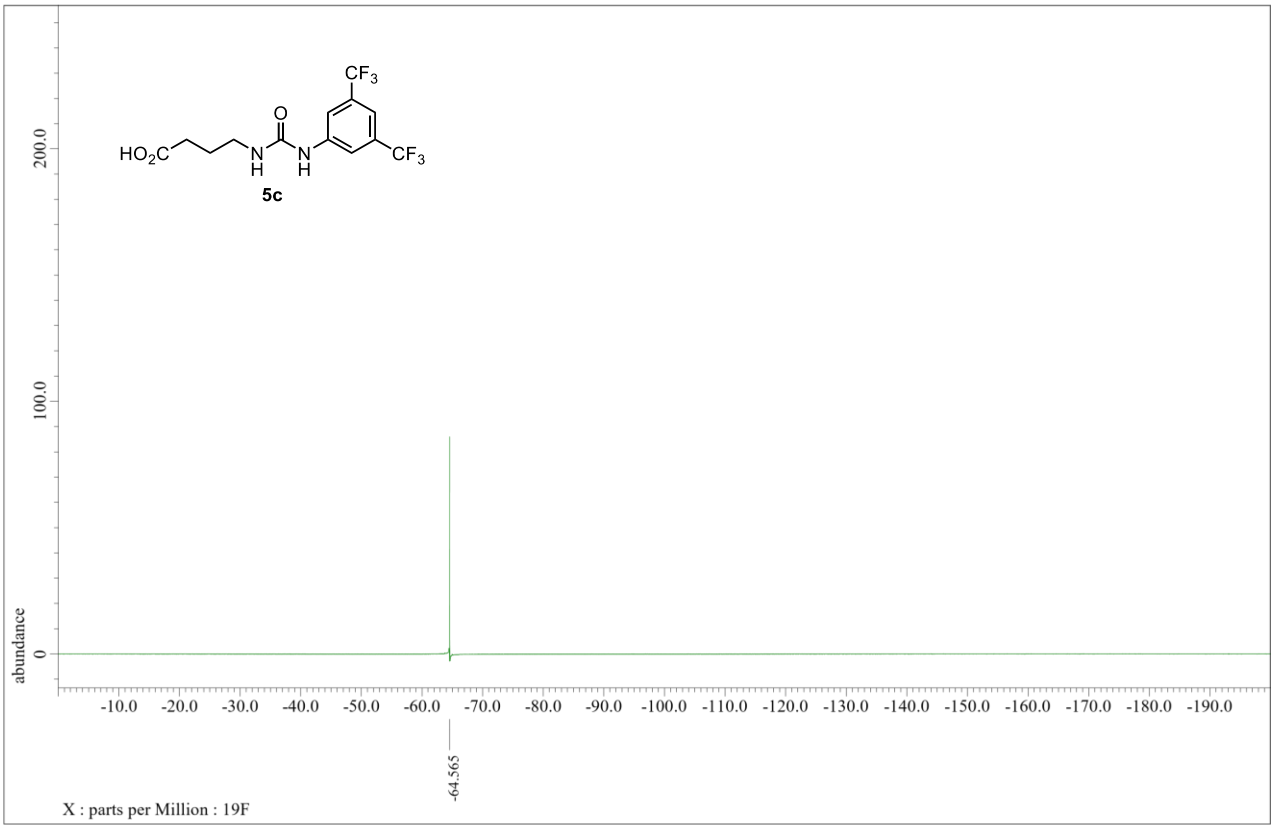

600 MHz, CD3OD

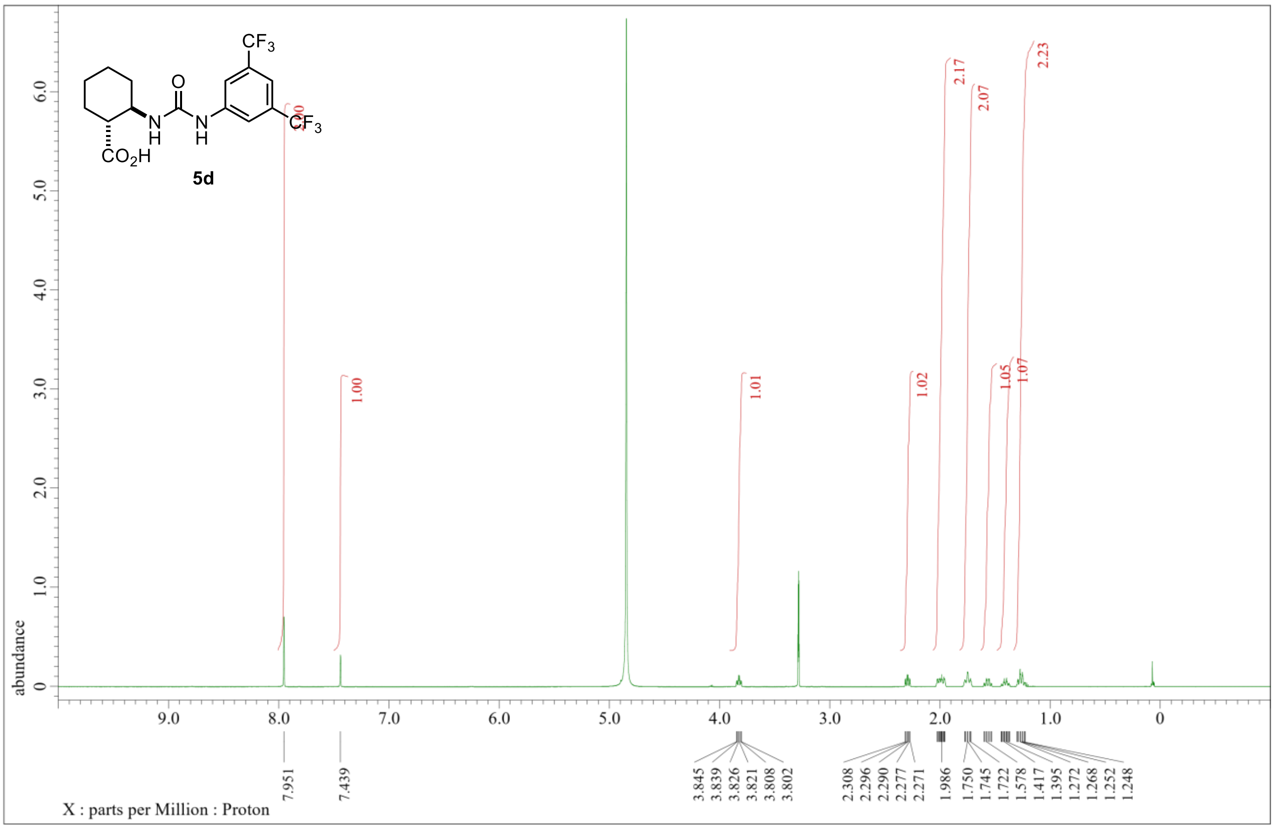

151 MHz, CD3OD

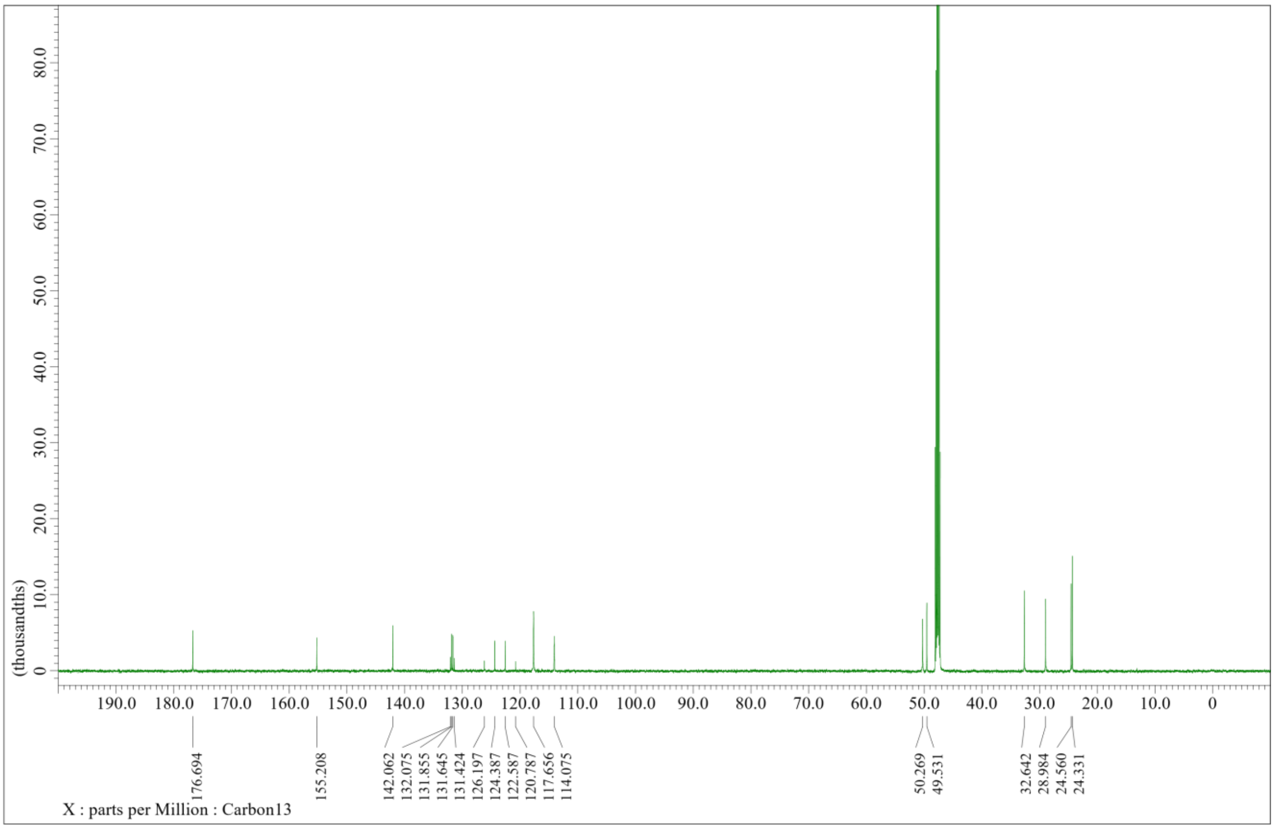

^{19}F NMR: 471 MHz, CD_3OD

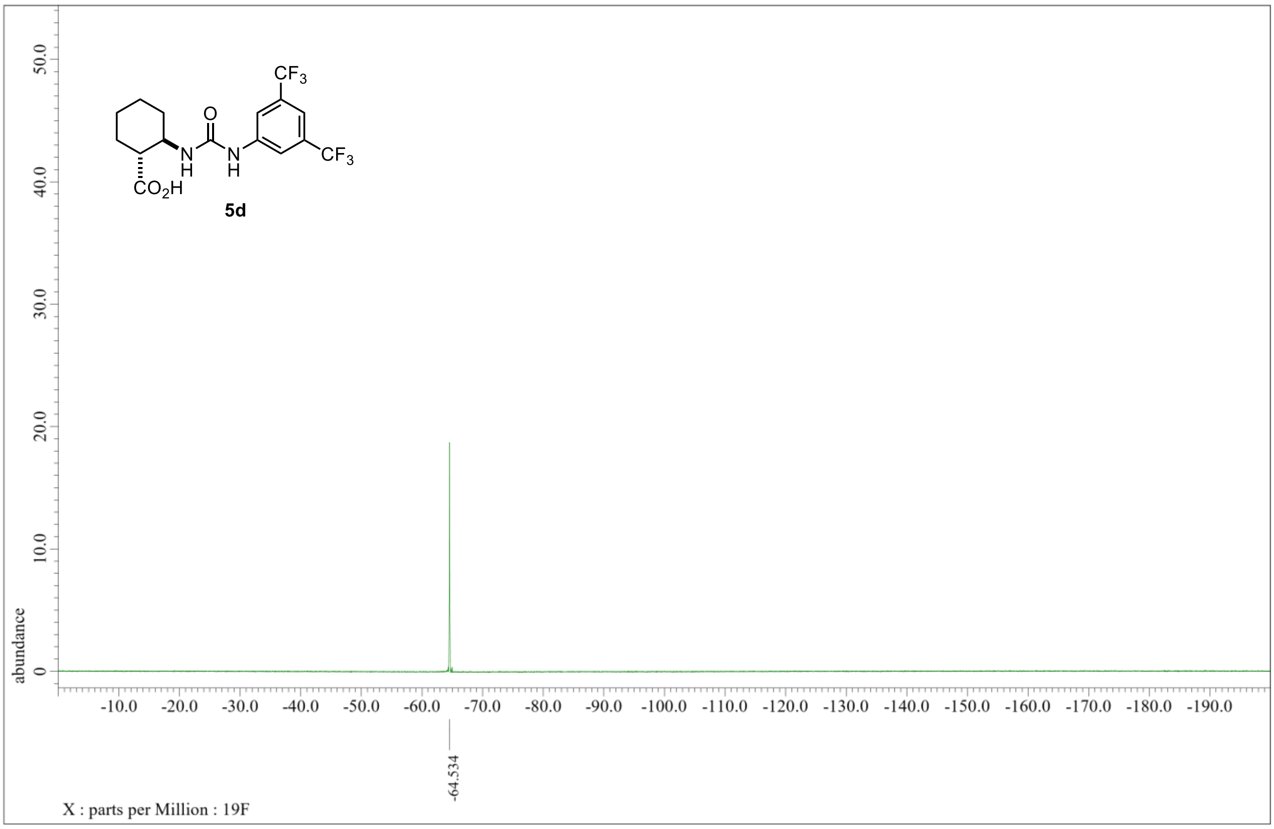

500 MHz, CD_3OD

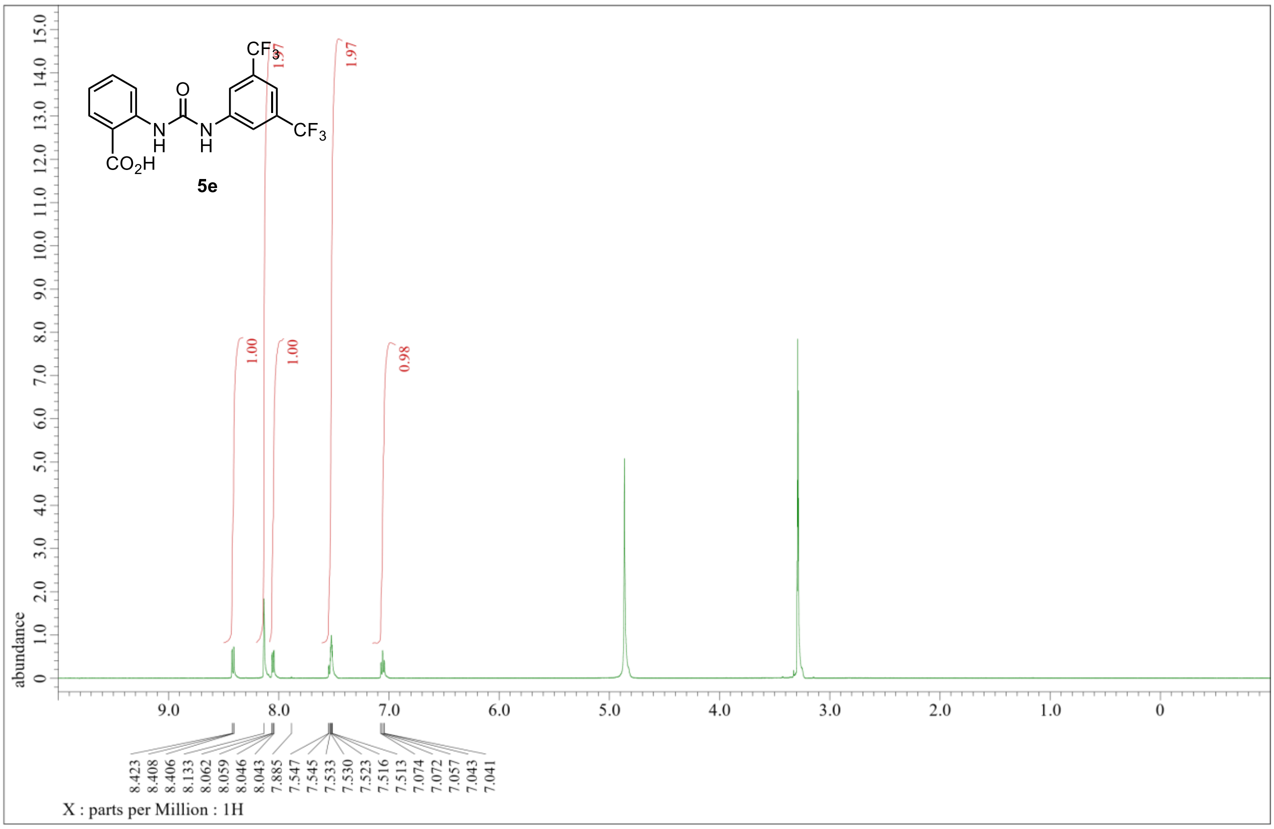

126 MHz, CD_3OD

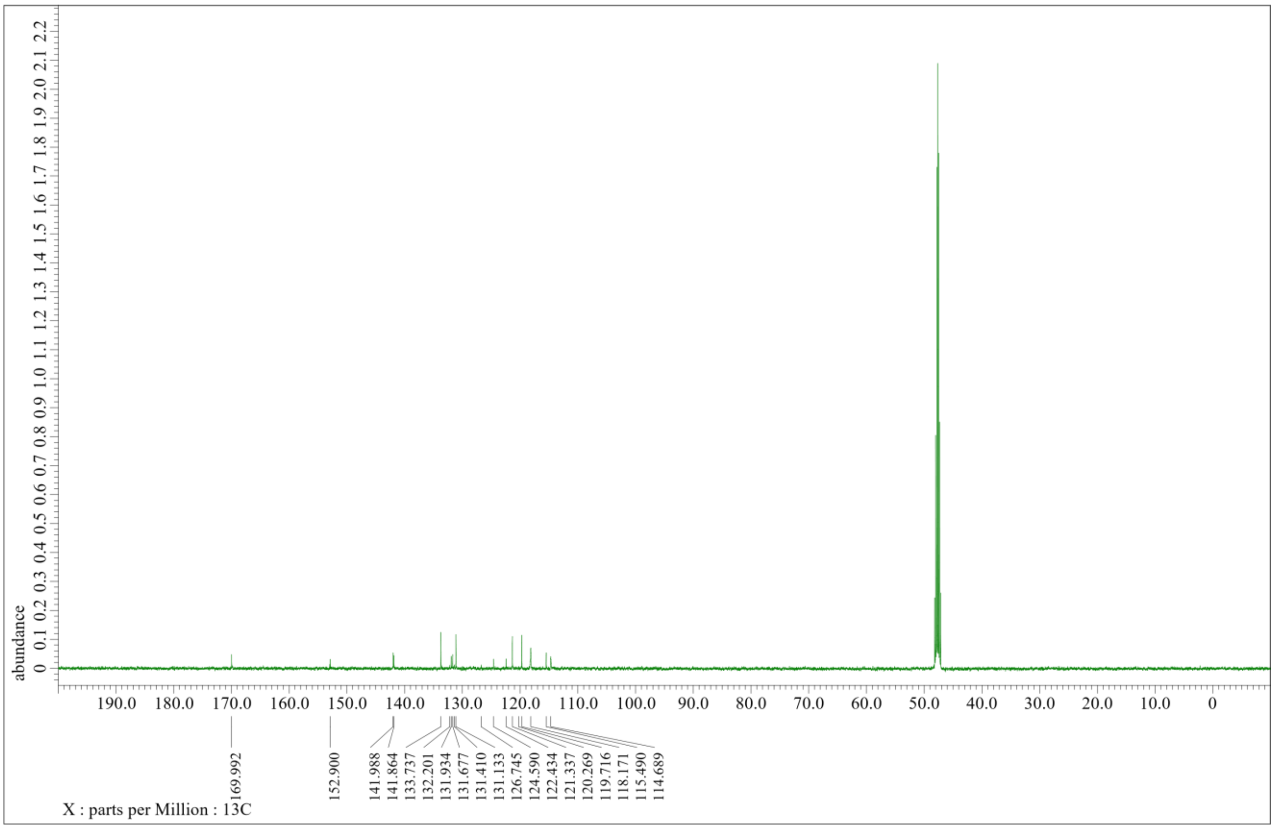

^{19}F NMR: 471 MHz, CD_3OD

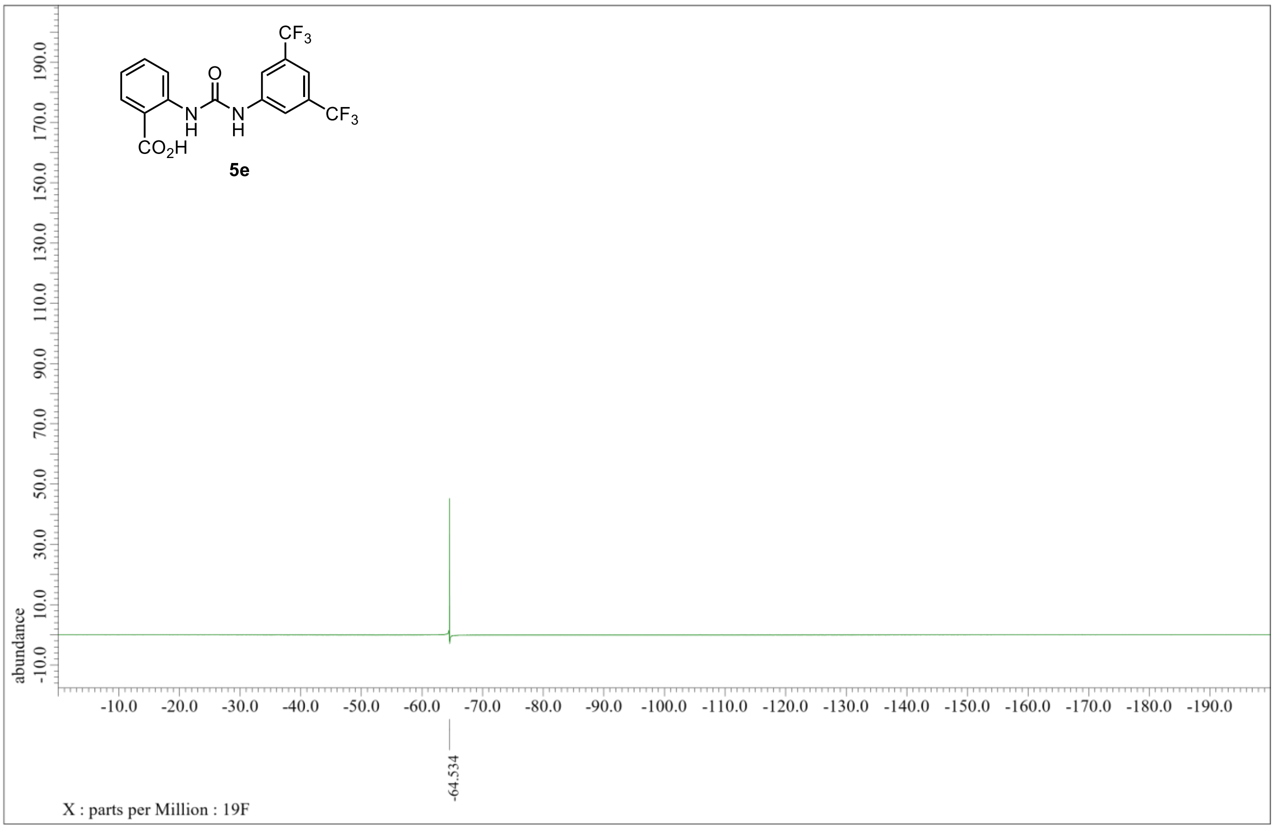

Supplement: Supplementary file 1 — The authors have cited additional references within the Supporting Information [67–76]. [file CHEM-32-e71082-s001.pdf]
